# Single-cell RNAseq reveals seven classes of colonic sensory neuron

**DOI:** 10.1136/gutjnl-2017-315631

**Published:** 2018-02-26

**Authors:** James R F Hockley, Toni S Taylor, Gerard Callejo, Anna L Wilbrey, Alex Gutteridge, Karsten Bach, Wendy J Winchester, David C Bulmer, Gordon McMurray, Ewan St John Smith

**Affiliations:** 1 Department of Pharmacology, University of Cambridge, Cambridge, UK; 2 Neuroscience and Pain Research Unit, Pfizer, Cambridge, UK

**Keywords:** colon, gastrointestinal, visceral sensory neurones, single-cell RNA sequencing

## Abstract

**Objective:**

Integration of nutritional, microbial and inflammatory events along the gut-brain axis can alter bowel physiology and organism behaviour. Colonic sensory neurons activate reflex pathways and give rise to conscious sensation, but the diversity and division of function within these neurons is poorly understood. The identification of signalling pathways contributing to visceral sensation is constrained by a paucity of molecular markers. Here we address this by comprehensive transcriptomic profiling and unsupervised clustering of individual mouse colonic sensory neurons.

**Design:**

Unbiased single-cell RNA-sequencing was performed on retrogradely traced mouse colonic sensory neurons isolated from both thoracolumbar (TL) and lumbosacral (LS) dorsal root ganglia associated with lumbar splanchnic and pelvic spinal pathways, respectively. Identified neuronal subtypes were validated by single-cell qRT-PCR, immunohistochemistry (IHC) and Ca^2+^-imaging.

**Results:**

Transcriptomic profiling and unsupervised clustering of 314 colonic sensory neurons revealed seven neuronal subtypes. Of these, five neuronal subtypes accounted for 99% of TL neurons, with LS neurons almost exclusively populating the remaining two subtypes. We identify and classify neurons based on novel subtype-specific marker genes using single-cell qRT-PCR and IHC to validate subtypes derived from RNA-sequencing. Lastly, functional Ca^2+^-imaging was conducted on colonic sensory neurons to demonstrate subtype-selective differential agonist activation.

**Conclusions:**

We identify seven subtypes of colonic sensory neurons using unbiased single-cell RNA-sequencing and confirm translation of patterning to protein expression, describing sensory diversity encompassing all modalities of colonic neuronal sensitivity. These results provide a pathway to molecular interrogation of colonic sensory innervation in health and disease, together with identifying novel targets for drug development.

**Significance of this study**

**What is already known on this subject?**

- Sensory dysfunction is implicated in the pathogenesis of GI disease, and the modulation of sensory pathways are an important site of action for existing pharmacotherapies.
- Colonic sensory neurons that control reflex pathways and give rise to sensations of urge, discomfort and pain are currently classified by differences in their sensitivity to mechanical stimuli and anatomical structure of their nerve endings.
- Existing schema propose five major subpopulations of colorectal afferents, while traditional molecular criteria describe a relatively homogenous population of peptidergic, voltage-gated sodium channel Na_V_1.8-expressing neurons; however, these do not fully encapsulate all sensory modalities present in the gut.

**What are the new findings?**

- Using single-cell RNA sequencing of mouse colonic sensory neurons, we describe a comprehensive framework for the molecular basis of visceral sensation from the colorectum.
- We identify seven distinct colonic sensory neuron subtypes with nerve-specific subtype diversity between the lumbar splanchnic nerve and pelvic nerve.
- We characterise a range of novel molecular markers for mouse colonic neuron subtypes facilitating interrogation of these pathways to GI function in health and disease.

**How might it impact on clinical practice in the foreseeable future?**

- Molecular fingerprinting of colonic sensory neurons will facilitate drug development in poorly treated conditions, such as IBS.
- Differential subtype expression of certain receptors likely explains the side effects of currently used medications.
- Identification of molecular markers for mouse colonic sensory neurons will enable targeted approaches exploiting transgenic models to accelerate our understanding of sensory physiology within the gut and the molecular mechanisms of clinically relevant pathologies, such as IBS.

## Introduction

The gastrointestinal (GI) tract is a complex set of organs responsible for the ingestion and digestion of food, absorption of nutrients and evacuation of waste. In addition to autonomic pathways, the GI tract can evoke behavioural changes by providing conscious awareness of damage, such as during injury, inflammation and infection, through sensory pathways to the central nervous system (CNS).^1^ In the colorectum, sensory innervation is organised into two main pathways: thoracolumbar (TL) spinal afferents projecting via the lumbar splanchnic nerve (LSN) and lumbosacral (LS) spinal afferents projecting via the pelvic nerve (PN) that are responsible for transducing conscious sensations of fullness, discomfort, urgency and pain, in addition to reflex actions.^2^

Visceral sensory afferents act to maintain many aspects of GI physiology, such as continence and evacuatory function and contribute to local regulation of motility, secretory processes and blood flow.^2^ Locally, peripheral visceral afferent fibres interact with enteroendocrine cells in the mucosa and immune cells in the gut wall to initiate and modulate sensory input.^3^ Sensory pathways are a major interface between luminal contents, including gut microbiota, the immune system and the CNS, providing the capacity to modulate mood and sensation.^4^ The importance of a healthy visceral sensory nervous system is demonstrated when these sensory pathways are dysregulated or maladapted, such as following spinal cord injury and in inflammatory bowel disease, where loss of effective bowel function and long-term chronic pain leads to significant morbidity.^5 6^ Indeed, in patients with IBS, chronic dysregulation of afferent signalling is thought to drive visceral hypersensitivity.^7^

Significant efforts have been taken to characterise the diversity of visceral afferent fibres innervating the colon in order to fully understand their function in both normal GI physiology and disease. Visceral afferents have been classified by morphological characteristics (eg, peripheral ending structure and location within the gut wall), by expression of ion channels, receptors and neurotransmitters and by functional criteria (eg, basal firing rate, mechanical activation threshold and stimulus-response functions). Consequently, a diverse range of visceral nerve classifications exist, which vary according to the particular pathway and species investigated, as well as the investigator and experimental protocols used. While recent schema to integrate this myriad of classifications proposes the presence of five major structural types of endings (mucosal, intraganglionic laminar, muscular-mucosal, intramuscular and vascular afferents^2^), traditional molecular criteria describe a relatively homogeneous population of peptidergic,^8^ voltage-gated sodium channel 1.8-expressing ‘nociceptors’.^9 10^

These classifications are constrained by the availability of discriminative markers to segregate neuronal subtypes, therefore limiting the application of advanced lineage-specific genetically encoded tools and are dependent on functional, often electrophysiological, categorisation to predominantly mechanical stimuli. Lastly, this schema does not enhance our understanding of the sensory contribution to the detection of non-mechanical stimuli and local effector function (eg, neurogenic inflammation).

Here, we conduct deep single-cell RNA sequencing of colon-projecting sensory neurons from the TL and LS spinal pathways in mouse and describe seven molecularly distinct subtypes of neuron. The identification of discriminative marker genes paves the way for targeted subtype-specific labelling, ablation and chemo/optogenetic and pharmacological interventions to elucidate the contribution of these sensory subtypes to GI physiology, including pain, in health and disease.

## Material and methods

For comprehensive descriptions of the methodologies used, see the online supplementary information.

10.1136/gutjnl-2017-315631.supp1 Supplementary data

### Isolation of mouse colonic sensory neurons

TL and LS dorsal root ganglia (DRG) were collected from healthy mice 3–10 days after retrograde tracing from the colon by Fast Blue (FB) injection using previously described methods.^11 12^ Individual colonic sensory neurons were manually picked by pulled glass pipette on an adapted inverted microscope after enzymatic dissociation of the DRG and were used in subsequent single-cell RNAseq (scRNAseq) and qRT-PCR analysis. Prior to picking, cells were photographed for cell size analysis.^10^ For immunohistochemistry (IHC), FB-labelled mice were perfused-fixed and whole TL and LS DRG processed.

### Single-cell RNAseq and clustering

Full-length cDNA from polyadenylated RNA of 399 colonic sensory neurons were generated using Smart-seq2 protocols^13^ and multiplexed into six pooled libraries using Illumina Nextera XT DNA Sample Kit before 75 bp paired-end sequencing on an Illumina NextSeq500 (read depth ~4.9M reads/cell). Sample-specific reads were aligned to the mouse reference genome (GRCm38.p3; Ensembl V.80) and genomic features determined using featureCounts. Low-quality cells were filtered (online supplementary figure 1) resulting in normalised data from 325 cells and 34 769 genes^14 15^ being passed onto downstream clustering analyses. Latent technical effects^14^ and contaminating satellite glia were conservatively removed^16^ (online supplementary figure 2) before clustering the remaining 314 cells based on gene expression profiles using single-cell consensus clustering (SC3)^17^ achieving greatest stability with *k*=7. Cluster robustness was investigated using bootstrapping of a downsampled dataset (online supplementary figure 3). Using SC3, we identified 709 marker genes capable of distinguishing individual clusters (area under receiver operator characteristic (AUROC)≥0.85 and P<0.01; online supplementary table 1).^17^ Data were visualised using R and the ggplot2 package.^18^ Sequence data that support the findings of this study have been deposited in Gene Expression Omnibus under accession code GSE102962. The expression profile of a given gene within colonic neuronal subtypes is provided at http://hockley.shinyapps.io/ColonicRNAseq.

10.1136/gutjnl-2017-315631.supp2 Supplementary data

10.1136/gutjnl-2017-315631.supp3 Supplementary data

10.1136/gutjnl-2017-315631.supp4 Supplementary data

10.1136/gutjnl-2017-315631.supp7 Supplementary data

### Single-cell qRT-PCR

Colonic sensory neuron populations determined by clustering of scRNAseq data were first validated using scqRT-PCR. Expression of mRNA transcripts for subpopulation-specific markers (*Cbln2*, *Mrgprd*, *Fam19a1*, *Smr2*, *Trpa1*, *Hpse* and *Ntm*) were evaluated in individual colonic sensory neurons by qRT-PCR as previously described.^10 12^ A z-score distribution of marker gene expression was determined after normalisation to *Gapdh* expression. Based on the relative expression of the seven markers genes, 168 neurons were assigned to colonic sensory neuronal subpopulations. In a subset of 83 neurons, the expression of *F2r*, *F2rl1*, *F2rl2* and *F2rl3* was also determined.

### Immunohistochemistry

In FB-labelled TL and LS DRG sections from perfused-fixed mice, specific antibodies against TrkC, Gfrα2, Gfrα3, Nrp1 and Spp1 were used to determine the relative expression of these proteins in colonic sensory neurons and further validate clusters derived from scRNAseq data. No labelling was observed where primary antibodies were excluded. Species-specific secondary antibodies conjugated to fluorophores (AlexaFluor-488/AlexaFluor-568) were used to visualise labelling. Relative intensity of immunostaining reaction products were determined for all colon-labelled DRG neurons with visible nuclei from 2 DRG per spinal segment from two to three animals.

### Ca^2+^ imaging and postfunctional classification

Ca^2+^ imaging of FB-labelled colonic sensory neuronal primary cultures was performed on an inverted Nikon Eclipse T*i* microscope following incubation with Fluo4-AM, a Ca^2+^-sensitive dye. Images were captured every second using a Zyla sCMOS camera for 60 s (10 s baseline, 15 s of compound application and 35 s washout). Subsequent compounds were applied at 5 min intervals in random order. KCl (50 mM) was applied at the end of the recording as a depolarising stimulus. FB-labelled neurons that responded to KCl were harvested by glass pipette and processed for scqRT-PCR to enable postfunctional classification based on the relative expression of the seven marker genes (*Cbln2*, *Mrgprd*, *Fam19a1*, *Smr2*, *Trpa1*, *Hpse* and *Ntm*).

## Results

### Identification of colonic sensory neuronal heterogeneity

**Figure 1:**
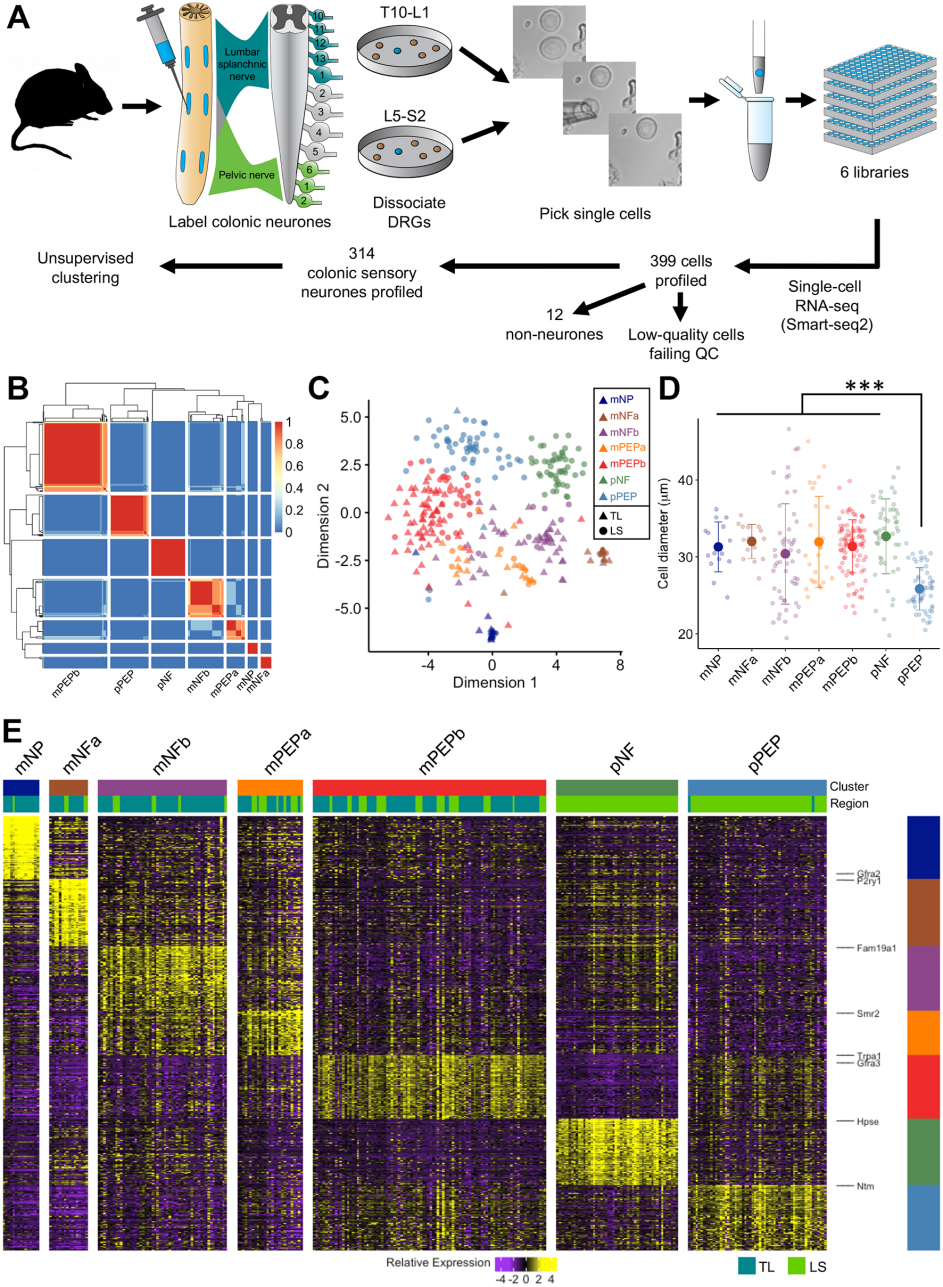
scRNAseq of sensory neurons innervating the colon reveals seven molecularly distinct cellular subtypes. (A) Flowchart depicting the experimental procedure. Sensory neurons innervating the colon *via* the LSN and PN were retrogradely labelled using FB. Individual labelled neurons were harvested from primary cultures of TL (T10-L1) and LS (L5-S2) DRG before processing for scRNAseq and unsupervised clustering. Images of before, during and after picking of a colonic sensory neuron are shown. (B) Similarity matrix indicating how often each pair of neurons is assigned to that cluster based on the clustering parameters. (C) t-SNE analysis of colonic sensory neurons (n=314). Numbers of profiled neurons per cluster: mNP (n=15), mNFa (n=16), mNFb (n=53), mPEPa (n=27), mPEPb (n=96), pNF (n=50), pPEP (n=57). Colours indicate unsupervised neuronal classification and shape indicates spinal segment from which the neuron was isolated (triangle, TL; circle, LS). Each dot represents an individual neuron. (D) Soma size analysis of colonic neuronal subtypes. Individual diameters of isolated neurons analysed by scRNAseq is overlaid with mean±SD for that subtype (***P<0.0001, one-way ANOVA with Tukey posthoc correction for multiple comparisons). (E) Heat map of scaled expression [log(TPM)] of marker genes for each of the colonic sensory neuronal subtypes as defined in B. Of the total 709 markers genes with AUROC≥0.85, only the top 100 genes for each cluster are displayed. Colour represents a *z*-score distribution from negative (purple) to positive (yellow). The spinal segmental region (TL or LS) from which an individual cell was isolated is shown below the subtype colour block. ANOVA, analysis of variance; AUROC, area under receiver operator characteristic; DRG, dorsal root ganglia; FB, Fast Blue; LS, lumbosacral; LSN, lumbar splanchnic nerve; mNFa; mNeuroFilament-a; mNP; mNonPeptidergic; mPEPa; mPeptidergic-a; PN, pelvic nerve; scRNAseq, single-cell RNAseq; TL, thoracolumbar.

**Figure 2:**
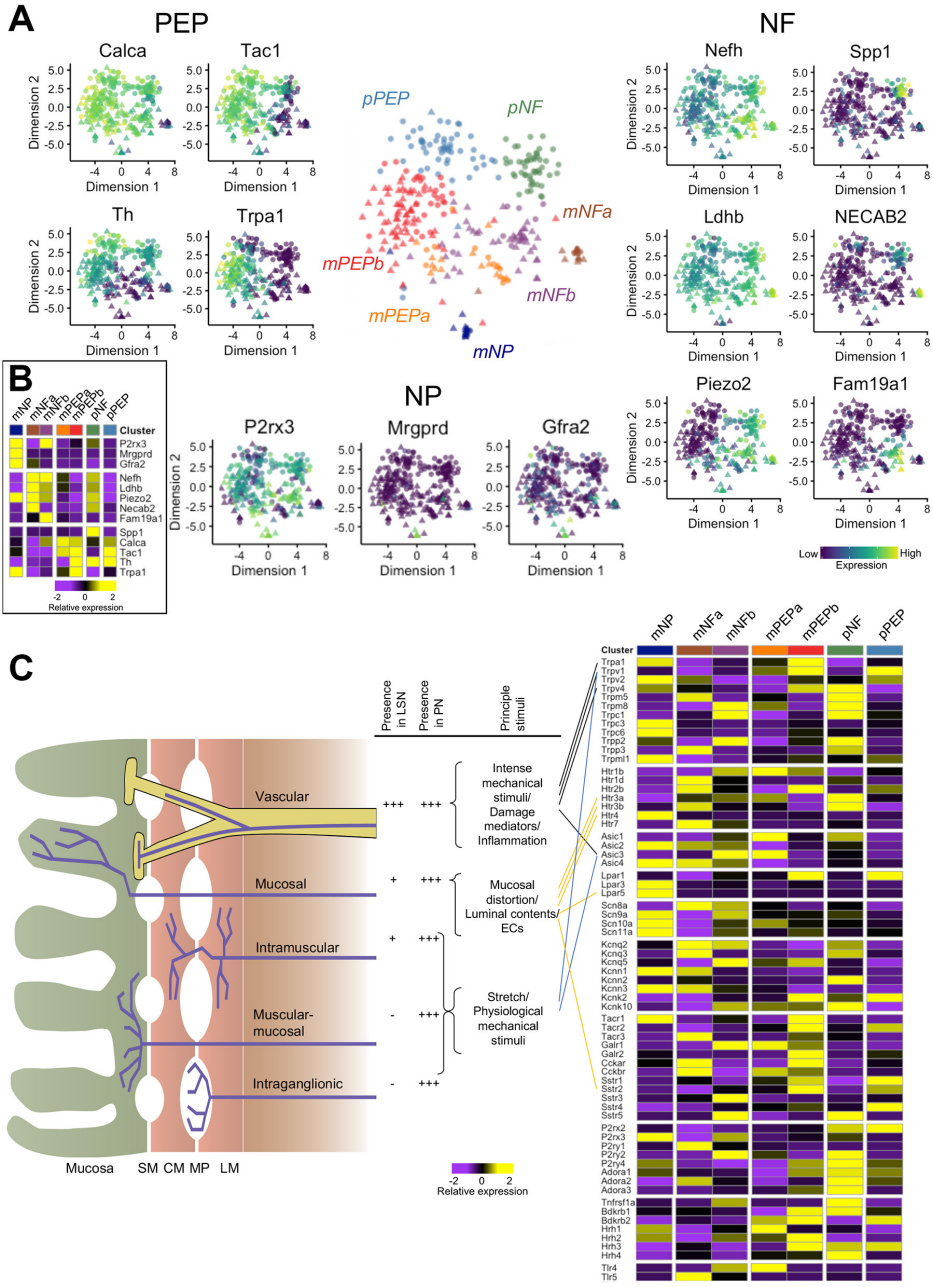
Definition of colonic neuronal subtypes identified by scRNAseq analysis. (A) t-SNE plot of colonic sensory neurons coloured by subtype. For different populations (‘peptidergic’, PEP ‘non-peptidergic’, NP and ‘neurofilament containing’, NF), relative expression of example genes are displayed (PEP: *Calca*, *Tac1*, *Th and Trpa1*; NP: *P2rx3*, *Mrgprd* and *Gfra2*; NF: *Nefh*, *Ldhb*, *Piezo2*, *NECAB2*, *Fam19a1* and *Spp1*). Colour scheme represents expression level [log(TPM)]. (B) Colonic sensory neuron cluster mean expression for the representative genes displayed in A. (C) Diagrammatic cross-section of the bowel wall showing existing classification schema for colonic afferents innervating the GI tract as defined by functional (eg, sensitivity to mechanical stimuli) and anatomical experiments.^2^ The frequency of colonic afferent classes differs in the two nerves innervating the colon of mouse and so does the principal stimulus to which a class is sensitive: these are displayed in the adjacent table (***high abundance, *low abundance and –absence). Next to this, mean subtype expression as defined by scRNAseq is shown for key ion channels/receptors and linked to colonic afferent classes where genetic ablation of ion channels and receptors has been shown to impair sensory function in a class-selective manner. *Calca* encodes CGRP; *Tac1*, Substance P; *Th*, tyrosine hydroxylase; *Trpa1*, Transient receptor potential cation channel member A1; *P2rx3*, P2X3; *Mrgprd*, MRGPRD; *Gfra2*, GDNF family receptor alpha-2; *Nefh*, neurofilament heavy chain; *Ldhb*, lactate dehydrogenase B; *Piezo2*, PIEZO2; *NECAB2*, N-terminal EF-hand Ca^2+^ binding protein 2; *Fam19a1*, Family with sequence similarity 19 member A1; *Spp1*, secreted phosphoprotein 1. CGRP, calcitonin gene-related peptide; CM, circular muscle; ECs, enterochromaffin cells; GDNF, glial-derived neurotrophic factor; LM, longitudinal muscle; MP, myenteric plexus; scRNAseq, single-cell RNAseq; SM, submucosa.

We performed deep scRNAseq using a modified Smart-seq2 protocol on 399 colonic sensory neurons innervating the mouse colon comprising almost equal proportions of cells originating from TL (T10-L1) and LS (L5-S2) DRG. Neurons were labelled by retrograde tracer (FB) injection into regions of the colon wall innervated by both nerves (figure 1A). In total, 1.136 billion paired-end reads were aligned to the mouse reference genome and used to determine individual gene expression levels. Of these, both low-quality cells and those with genes from potentially confounding satellite glia were excluded (see online supplementary methods and figures 1 and 2). This resulted in 314 colonic sensory neurons with an average 3.4 million reads mapping to 9675±1796 unique genes per cell being taken forward into subsequent clustering analyses.

We defined seven clusters in an unbiased fashion by *k*-means-based consensus clustering (figure 1B).^17^ We observed spinal column regional differences in the presence of subtypes identified. With the exception of two cells, colonic sensory neurons isolated from TL DRG (n=159) were grouped into five subtypes based on gene expression profiles, while those neurons isolated from LS DRG (n=155) were split into seven subtypes with two almost exclusively LS in composition (98%) and 32% of neurons showing similarity to the main five TL subtypes (figure 1C and online supplementary figure 4A). Of the five ‘mixed’ subtypes (populated by both TL and LS neurons and identified with the prefix ‘m’), we identify two subtypes (mNeuroFilament-a (mNFa) and mNeuroFilament-b (mNFb)) comprising 16 and 53 neurons, respectively (figure 2A). These two subtypes have greater expression of genes typically associated with myelinated DRG neurons including neurofilament heavy chain (*Nefh*) and lactate dehydrogenase B (*Ldhb*),^19^ but can be distinguished by N-terminal EF-hand Ca^2+^ binding protein 2 (*NECAB2*) and family with sequence similarity 19 member A1 (*Fam19a1*) expression, respectively. The third subtype, mNonPeptidergic (mNP), made up of 15 cells showed expression of purinergic receptor P2X3 (*P2rx3*), MAS-related GPR, member D (*Mrgprd*) and glial-derived neurotrophic factor family receptor α2 (*Gfra2*), all previously associated with non-peptidergic nociceptors^20^ and predicted to account for very few colonic sensory neurons based on previous literature reporting the prominence of peptidergic neurons innervating the colon.^8^ Collectively, mNP and mNF subtypes also all express the mechanotransducer Piezo2 (figure 2A and B). The final two mixed subtypes (mPeptidergic-a (mPEPa), 27 cells and mPEPb, 96 cells) express calcitonin gene-related peptide (CGRP; *Calca*), substance P (*Tac1*) and TrkA (*Ntrk1*) and represent peptidergic nociceptors; mPEPb expresses transient receptor potential cation channel A1 (*Trpa1*), while mPEPa also expresses acid-sensing ion channel 3 (*Asic3*) and Toll-like receptor 4 (*Tlr4*, figure 2A and online supplementary figure 4B). The two additional subtypes identified, which were populated almost exclusively by LS colonic neurons, are given the preface ‘pelvic’ (‘p’) due to LS DRG cell bodies innervating the colon *via* the PN. The sixth, the pNF subtype, was made up of 50 cells, which like the mNF subtypes expresses *Nefh*, *Ldhb* and *Piezo2*, but additionally expresses TrkB (*Ntrk2*), *Asic1* and secreted phosphoprotein 1 (*Spp1*) suggesting a molecular and spinal region, distinct population to mNFa and mNFb (figure 2A, B and online supplementary figure 4B). The seventh subtype, pPEP (57 cells), was peptidergic (expressing *Calca* and *Tac1*), but differed from mPEPa/mPEPb by high expression of tyrosine hydroxylase (*Th*) and the absence of *Trpa1* (figure 2B). These pelvic subtypes showed some similarity to subtypes mNFa/mNFb and mPEPa/mPEPb, respectively, but they possess distinct expression patterns suggesting functional disparity between classes of neuron innervating the colon *via* different nerves. Our analysis supports the hypothesis that a restricted diversity of sensory neurons innervate visceral organs,^21^ but that this diversity is far richer than previously recognised from functional/anatomical analyses.

10.1136/gutjnl-2017-315631.supp5 Supplementary data

The mean soma diameter of TL neurons profiled was 31.3±0.4 µm and, in agreement with previous studies, was significantly greater than for LS neuron soma diameter (29.6±0.4 µm; n=152–157, P=0.006, 95% CI 0.60 to 2.80, two-sided unpaired t-test).^8 10^ Determining mean soma diameter for each subtype revealed that pPEP neurons were significantly smaller than any other subtype (P<0.0001, one-way analysis of variance with Tukey posthoc correction for multiple comparisons; figure 1D), supporting the hypothesis based on transcript expression that these pelvic sensory neurons are distinct from other peptidergic subtypes (mPEPa/mPEPb) identified. We identified 709 genes (AUROC≥0.85 and P<0.01) that best classified colonic sensory neurons into these seven subtypes (figure 1E and online supplementary table 1; a violin plot of any gene can be visualised at http://hockley.shinyapps.io/ColonicRNAseq/).

We next investigated the expression profiles of ion channels and receptors known to contribute to sensory (mainly mechanosensitive) function in the GI tract. Vascular afferents are the principal functional class innervating the gut via the LSN, while the PN is more evenly populated by all afferent classes (figure 2C).^2^ Selective deficits in these classes have been observed following genetic ablation of transducer channels (such as *Trpa1*,^22^ *Trpv1*,^23^ *Trpv4*^24^ and *Asic3*^23^). In order to understand how our analysis of colonic sensory neurons fits with existing schema for classifying gut afferents, we assessed the relative expression of key ion channels and receptors within our seven subtypes (figure 2C and online supplementary figure 4B). Compared with other subgroups, *Trpa1*, *Trpv1* and *Trpv4* are most highly expressed in mPEPb neurons suggesting that these cells may represent (a portion of) the functionally defined vascular afferent class. By contrast, *Asic3* showed greatest expression in mNFb and mPEPa subgroups. Protease-activated receptor 2 (PAR2; encoded by *F2rl1*), an important modulator of visceral pain,^25^ is broadly not detected, while PAR1 (*F2r*) is most prevalent in mNFb and pNF groups, PAR3 (*F2rl2*) is found fairly ubiquitously and PAR4 (*F2rl3*) in a small number of cells in each subtype (online supplementary figure 5).

10.1136/gutjnl-2017-315631.supp6 Supplementary data

Enterochromaffin cells (ECs) residing in mucosal villi release 5-hydroxytryptamine (5-HT) and directly couple sensory nerves via 5-HT_3_ receptors, thus acting as important gut chemosensors.^26^ We find abundance of 5-HT_3_ receptor subunits (both *Htr3a* and *Htr3b*) in NF colonic neuronal subtypes (figure 2C). By contrast, the 5-HT_4_ receptor subunit (*Htr4*, figure 2C) is only present on mNP neurons. Intriguingly, lysophosphatidic acid receptor 5 (encoded by *Lpar5*), a specific GPCR chemosensor activated by partially digested dietary protein (peptone) and present in afferents terminating in the mucosa,^27^ is coexpressed by mNP neurons. Therefore, our data support the conclusion that both NF and mNP neurons may represent subtypes of mucosal afferent tuned to respond to serotonergic signalling and act to transduce luminal contents.

What is clear from the analysis is that multiple mechanosensitive channels exist within the same colonic neuronal subtype (eg, *Trpv1* and *Trpa1* in mPEPb) and that redundancy also exists in channel expression across multiple subtypes (eg, *Trpa1* in mNP, mPEPb and to a lesser extent mPEPa and pPEP subtypes), and this indicates that direct mapping of current functional classifications to identified scRNAseq subtypes is not feasible and that existing schema inadequately describe sensory diversity innervating the colon.

### Predicted colonic sensory neuronal subtypes can be recapitulated by single-cell qRT-PCR using seven marker genes

**Figure 3:**
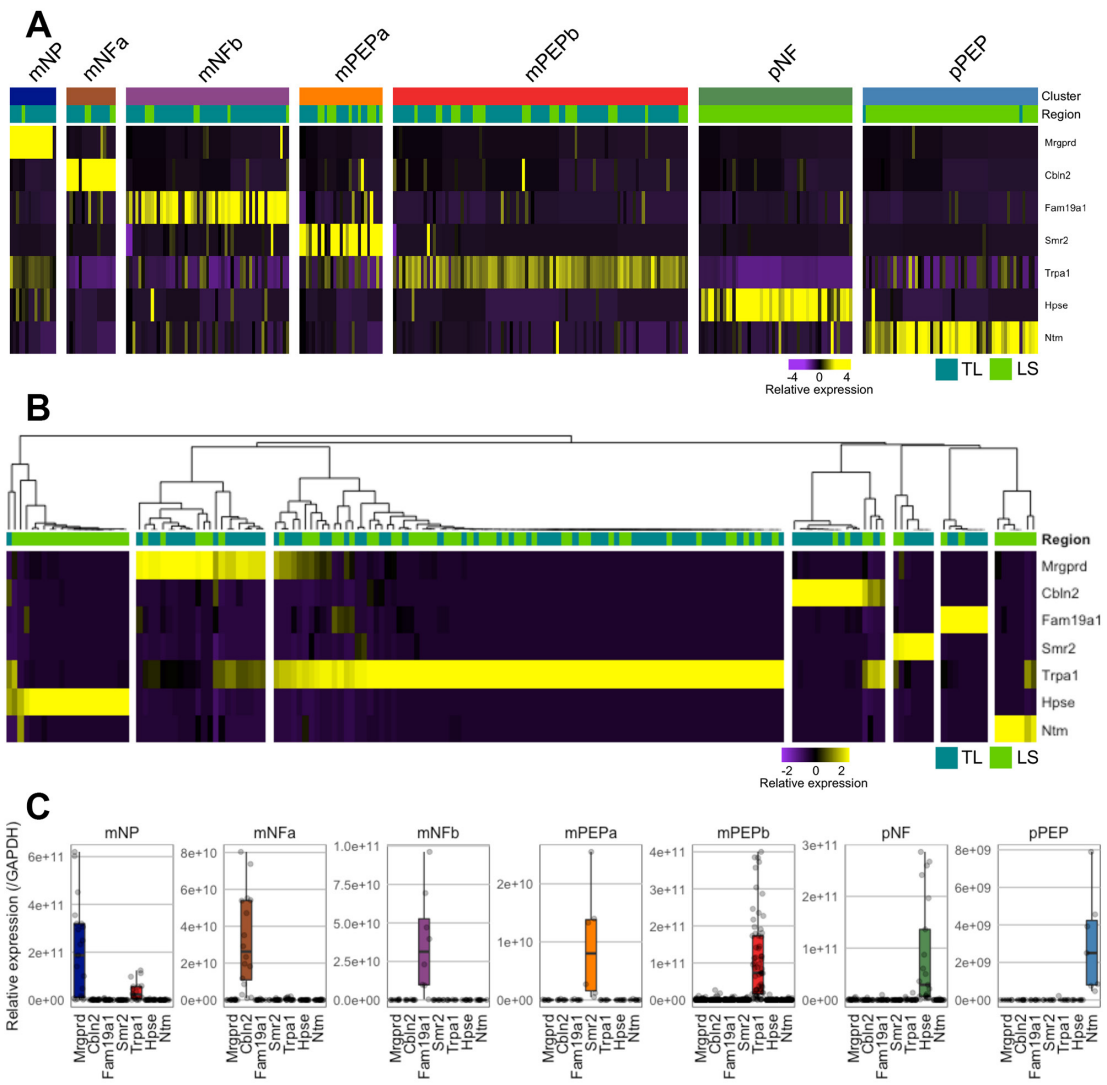
Validation of colonic sensory subtypes by scqRT-PCR. (A) Heat map scaled expression of seven representative marker genes from scRNAseq analysis, one for each colonic neuronal subtype. Colour represents a *z*-score distribution from negative (purple) to positive (yellow). (B) Heat map and hierarchical clustering of colonic sensory neurons from secondary cohort (n=168) based on scqRT-PCR expression of the seven representative marker genes (*Mrgprd*, *Cbln2*, *Fam19a1*, *Smr2*, *Trpa1*, *Hpse* and *Ntm*). Based on this hierarchical clustering, neurons were assigned to one of the seven subtypes (mNP, 22/168 (13%); mNFa, 16/168 (10%); mNFb, 8/168 (5%); mPEPa, 7/168 (4%); mPEPb, 87/168 (52%); pNF, 21/168 (13%) and pPEP, 7/168 (4%)). With one exception, only neurons isolated from LS DRG possessed high expression by scqRT-PCR of markers for pNF (ie, *Hpse*) and pPEP (ie, *Ntm*) subtypes as predicted by scRNAseq. (C) Box and whisker plot overlaid with individual data points showing expression (centre line, median; box limits, 25th and 75th percentiles; whiskers, 1.5x IQR; outliers are not displayed) of the marker genes within each group of neurons assigned to a specific subtype based on the clustering from B. DRG, dorsal root ganglia; LS, lumbosacral; mNFa; mNeuroFilament-a; mNP; mNonPeptidergic; mPEPa; mPeptidergic-a; scRNAseq, single-cell RNAseq.

In order to validate the seven subtypes identified by scRNAseq, we evaluated the expression of seven marker genes predicted to be uniquely present within one of each of the subtypes. Specifically, we examined the expression of *Mrgprd* (mNP selective marker), cerebellin 2 precursor protein (*Cbln2*; mNFa selective marker), submaxillary gland androgen regulated protein 2 (*Smr2*; mPEPa selective marker), family with sequence similarity 19, member A1 (*Fam19a1*; mNFb selective marker), *Trpa1* (mPEPb selective marker), heparanase (*Hpse*; pNF selective marker) and neurotrimin (*Ntm*; pPEP selective marker) in both colonic TL and LS neurons by single-cell qRT-PCR (scqRT-PCR; figure 3A shows scRNAseq expression profiles of these genes). Of the 168 neurons sampled from 6 mice, TL neurons had a mean soma diameter of 32.3±0.6 µm (n=84) compared with 30.3±0.5 µm for LS neurons (n=83; size data for one neuron was not collected). Individual colonic neurons tended to only express one of the seven marker genes (*Mrgprd*, *Cbln2*, *Fam19a1*, *Smr2*, *Trpa1*, *Hpse* and *Ntm*; figure 3B) to a high degree and not the other six, validating the mutual exclusivity of the markers predicted by the scRNAseq clustering and their utility in allocating neurons into one of the seven subtypes. As such, based on the scqRT-PCR expression profiles, neurons were assigned as mNP (n=22), mNFa (n=16), mNFb (n=8), mPEPa (n=7), mPEPb (n=87), pNF (n=21) and pPEP (n=7; figure 3B) subtypes. While neurons from all seven subtypes were represented in this experiment, the low frequency of some groups (particularly mPEPa and pPEP) did not allow investigation of soma diameter as a function of classification. In further confirmation of the scRNAseq characterisation, with the exception of a single neuron, only neurons originating from LS DRG expressed the pelvic cluster markers (*Hpse* and *Ntm*) and were grouped into one of the two pelvic subtypes (pNF and pPEP). Although each marker gene was the predominant gene expressed by cells within that subtype, the presence of other marker genes was observed, but to a far lesser extent (notably, in agreement with RNAseq analysis, *Trpa1* expression was observed in some mNP neurons; figure 3C). Additionally, in a subset of 83 neurons, we validate PAR1-4 expression by scqRT-PCR, showing comparable proportions to that obtained by scRNAseq, that is, almost complete absence of PAR2, broad expression of PAR3 and more discrete expression of PAR1 and PAR4 (online supplementary figure 5).

### Validating colonic sensory neuronal groups by immunohistochemistry

**Figure 4:**
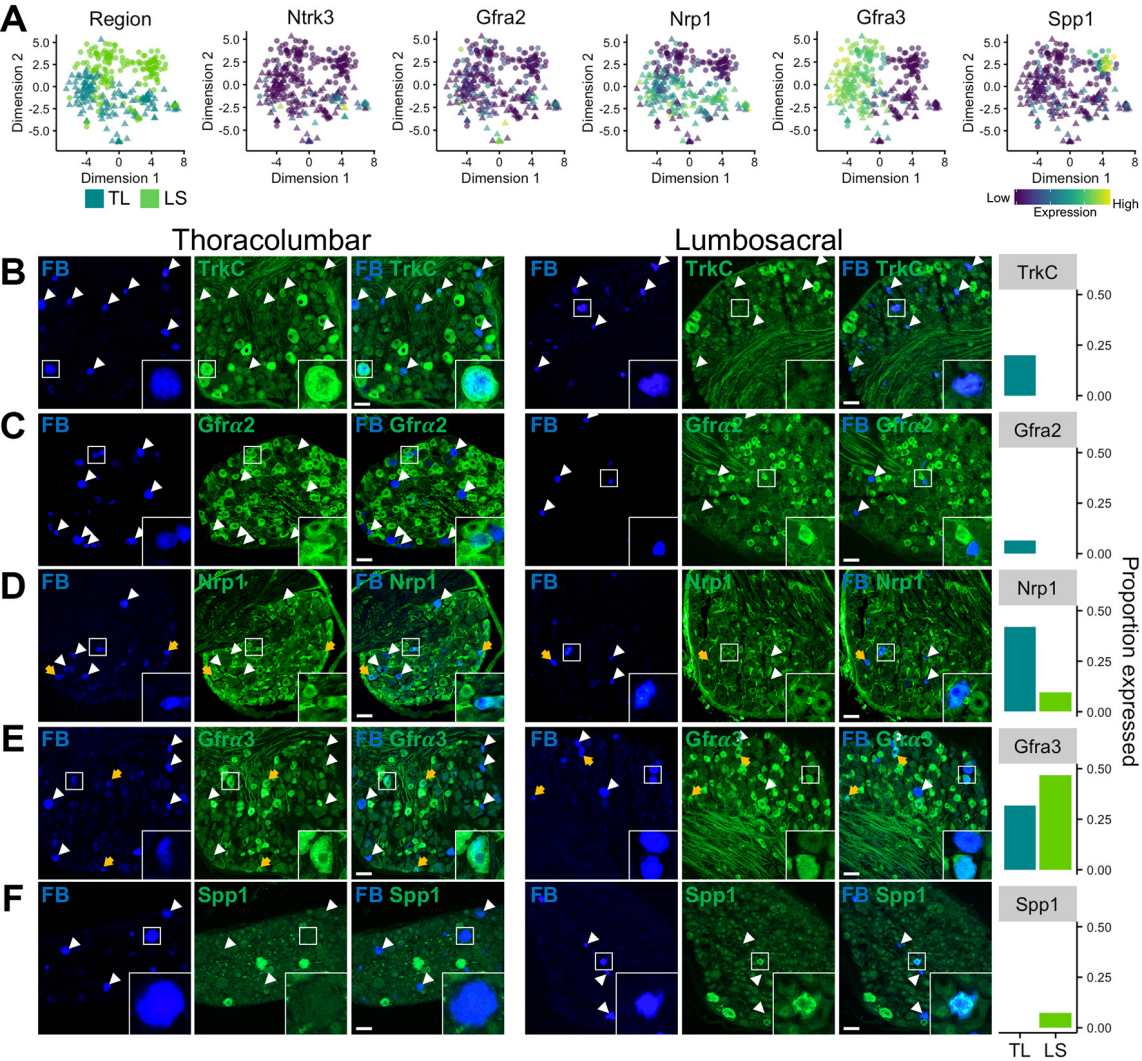
*In vivo* validation of colonic sensory neuronal expression profiles. (A) t-SNE plots of scRNAseq analysis overlaid with colour depicting either spinal segmental region (eg, TL or LS) the neuron was isolated from or expression for genes (*Ntrk3* encodes TrkC; *Gfra2*, GDNF family receptor α 2; *Nrp1*, neuropilin 1; *Gfra3*, GDNF family receptor α 3 and *Spp1*, secreted phosphoprotein 1 used for *in vivo* validation). Colour scheme represents expression level [log(TPM)]. Immunohistochemical investigation of markers in colonic sensory neurons labelled with FB in sections of TL (left) and LS (right) DRG. (B) In agreement with scRNAseq expression profiles, FB+/TrkC+ neurons were only observed in TL, but not LS, DRG. *TL inset*, FB+/TrkC+ neuron. *LS inset*, FB+/TrkC- neuron. (C) FB+/Gfrα2+ neurons were only observed in TL, but not LS, DRG. *TL inset*, example of both FB+/Gfrα2+ and FB+/Gfrα2– neurons. *LS inset*, example of FB+/Gfrα2– and FB–/Gfrα2+ neurons. (D) FB+/Nrp1+ neurons were present in TL DRG and to a lesser extent LS DRG. *TL inset*, FB+/Nrp1+ and FB–/Nrp1+ neurons. *LS inset*, FB+/Nrp1+ and FB–/Nrp1+ neurons. (E) FB+/Gfrα3+ neurons were present in significant proportions of both TL and LS DRG. *TL inset*, FB+/Gfrα3+ neuron. *LS inset*, examples of FB+/Gfrα3+ and FB+/Gfrα3– neurons. (F) FB+/Spp1+ neurons were only observed in LS, but not TL, DRG. *TL inset*, FB+/Spp1– neuron. *LS inset*, FB+/Spp1+ neuron. An example for each marker from both TL and LS DRG is shown with FB labelling, marker immunostaining and a combined overlay from the same field of view. Yellow arrows represent FB+ neurons showing expression of the marker of interest. White arrowheads represent FB+ neurons that do not express the marker of interest. *Right*, proportion of FB+ neurons expressing the marker of interest in TL and LS DRG from two mice (2 DRG per spinal segment per mouse). Scale bar 50 μm, which is consistent across all images. Each inset image is 56×56 μm. DRG, dorsal root ganglia; FB, Fast Blue; GDNF, glial-derived neurotrophic factor; LS, lumbosacral; scRNAseq, single-cell RNAseq; TL, thoracolumbar.

In order to confirm that the molecular patterning observed by mRNA transcript expression using scRNAseq and scqRT-PCR translated to protein expression at the level of the cell body in intact ganglia, we performed IHC on sections from retrogradely labelled mouse TL (T13 and L1) and LS (L6 and S1) DRG following injection of FB into the colon wall. Immunostaining was performed using antisera raised against tyrosine receptor kinase C (TrkC, encoded by *Ntrk3*), Gfrα2, neuropilin 1 (Nrp1), Gfrα3 and Spp1: proteins selected based on the availability of well-validated antibodies used previously in mouse DRG tissues and on their predicted scRNAseq mRNA expression to delineate neuronal populations. Indeed, *Ntrk3* and *Gfra2* are expressed by predominantly TL populations (mNP/mNFa/mNFb and mNP/mNFa, respectively), while *Spp1* is only expressed by pNF subtype of LS derived pelvic neurons (figure 4A). Therefore, we would predict that TrkC and Gfrα2 would have a low frequency of expression within LS neurons and that Spp1 will not be present at all in TL neurons. Indeed, TrkC labelling was observed in a fifth of TL neurons (31 of 154) with an average TrkC-positive (TrkC+) diameter of 34.3±1.4 µm, whereas, no LS neuron immunostained for TrkC (figures 4B and 0 of 152). For Gfrα2, labelling was present in 6% of TL (17 of 265), but not in any LS neuron (0 of 201), with a Gfrα2+ cell diameter of 21.0±1.1 µm (figure 4C). Labelling of Nrp1 revealed a bias towards expression within TL, over LS, neurons (TL, 42%, 70 of 167 cells (diameter, 25.3±0.6 µm) vs LS, 10%, 14 of 145 cells (diameter, 26.9±2.2 µm), figure 4D), as predicted by the relatively broad, although not high, expression within mNFa, mNFb, mPEPa and mPEPb subgroups observed by scRNAseq (figure 4A). Furthermore, mRNA transcripts for Gfrα3 were selectively expressed within peptidergic subgroups coexpressing *Trpv1*, *Tac1* and *Calca*, from both TL (mPEPb and to a lesser extent mPEPa) and LS (pPEP) DRG. Immunolabelling for Gfrα3, in line with the predicted mPEPa and pPEP expression, revealed significant staining in both TL (32%, 33 of 104) and LS (47%, 49 of 105) neurons (figure 4E). In agreement with the scRNAseq data, the average soma diameter for Gfrα3+LS neurons (pPEP neurons; 20.8±0.8 µm) was significantly smaller than Gfrα3+TL neurons (mPEPb neurons; 24.3±0.9 µm vs Gfrα3+LS neurons, P=0.0055, n=33–49, 95% CI 1.06 to 5.93, two-sided unpaired t-test), suggesting that Gfrα3 is expressed broadly in peptidergic neuronal subgroups, but that segmental differences exist between the subtypes present in different nerves. Lastly, Spp1, a selective marker for pNF subtype, was present in 6% of LS neurons (6 of 96; diameter 20.6±1.3 µm) only and was not observed in any TL neurons (0 of 281; figure 4F).

### Functional diversity of colonic sensory neurons

**Figure 5:**
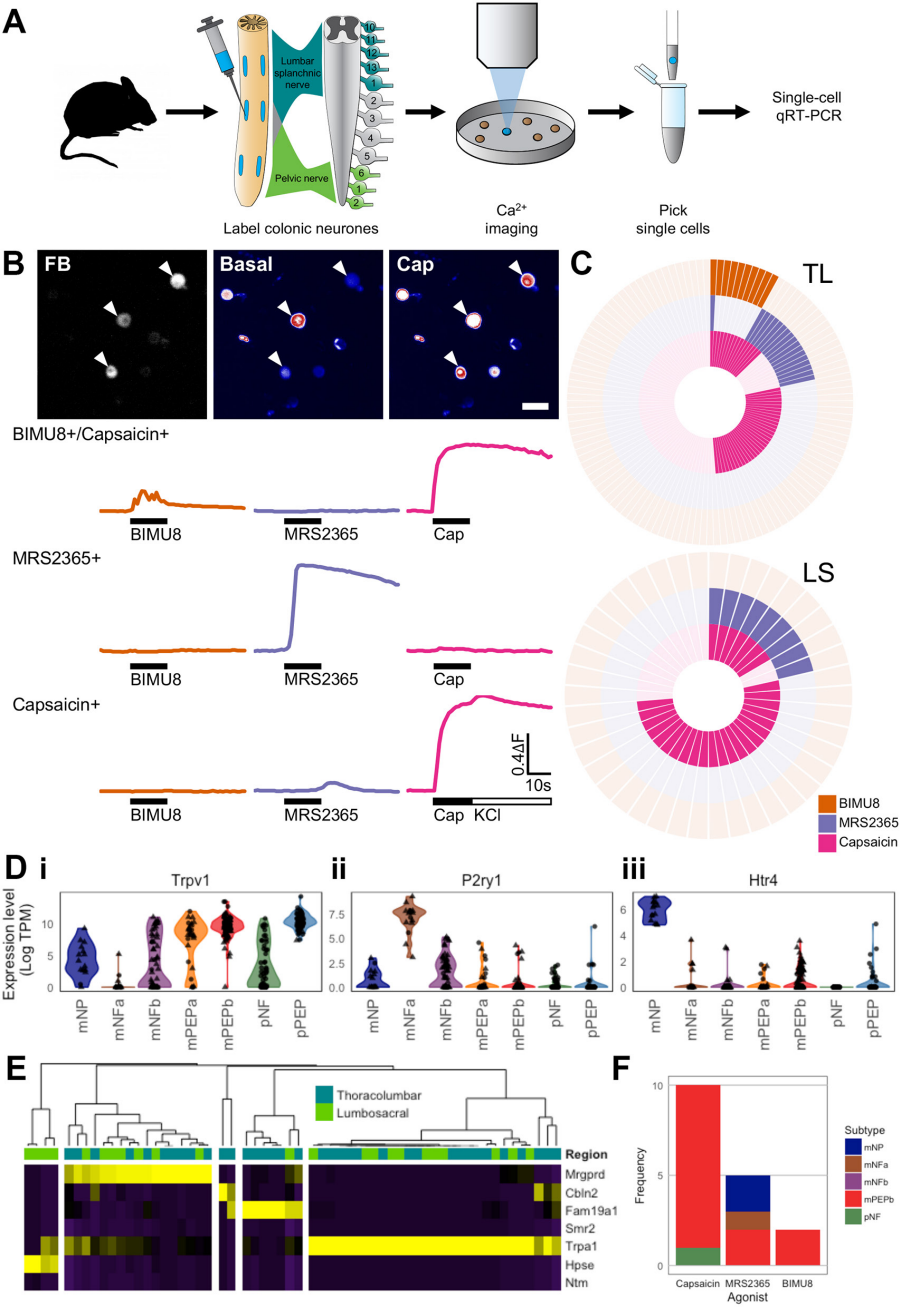
Colonic sensory neuron subtypes are functionally heterogeneous. (A) Flowchart depicting the experimental procedure undertaken. Neuronal activity was assessed by measurement of [Ca^2+^]_i_ in FB labelled neuron isolated from both TL and LS DRG in response to different stimuli. After imaging, FB+ cells were picked by glass pipette, scqRT-PCR performed to determine expression of seven cluster-specific genes (*Mrgprd*, *Cbln2*, *Fam19a1*, *Smr2*, *Trpa1*, *Hpse* and *Ntm*) and neurons assigned to colonic sensory subtypes. (B) Examples of increases in [Ca^2+^]_i_ in colonic sensory neurons measured using fluo-4. *Top*, fluo-4 fluorescence under basal and after addition of capsaicin in colonic sensory neurons labelled with FB. Arrowheads highlight FB+ neurons. Scale bar 50 μm. *Bottom*: example traces of increases in [Ca^2+^]_i_ of a 5-HT_4_/TRPV1-coexpressing neuron in responses to BIMU8 (orange) and capsaicin (pink), a P2Y_1_-expressing neuron responding to MRS2365 (purple) only and a TRPV1-expressing neuron responding to capsaicin only. (C) Analysis of BIMU8, MRS2365 and capsaicin responsiveness in both TL (*top*) and LS (*bottom*) colonic sensory neurons. Each segment represents a single neuron with a filled coloured block signifying a positive response to agonist addition. (D) Violin plots of *Trpv1*, *P2ry1* and *Htr4* expression levels [Log(TPM)] from scRNAseq analysis of colonic neurons split and coloured by seven subtypes based on clustering from figure 1B. Spinal segmental region from which the neuron was isolated is represented by shape (triangle, TL; circle, LS). *Trpv1* has broad expression across a range of subtypes, most notably mPEPb and pPEP. *P2ry1* is highly expressed by mNFa neurons, which do not express either *Htr4* or *Trpv1*. Low level coexpression of *P2ry1* and *Trpv1* is observed in mNP and mNFb subgroups. In agreement with functional profiles from figure 5C, *Htr4* is predominantly expressed by mNP neurons (and to a lesser extent mPEPb), which coexpress low levels of *Trpv1*. (E) Heat map and hierarchical clustering by scqRT-PCR of colonic sensory neurons harvested postfunctionally (n=59). Neurons with high expression of *Smr2* (marker for mPEPa) and *Ntm* (marker for pPEP) were not detected in this cohort and neurons were therefore classified into one of the remaining five subgroups (mNP, 17/59 (29%); mNFa, 2/59 (3%); mNFb, 7/59 (12%); mPEPb, 29/59 (49%) and pNF, 4/59 (7%)). Colour scheme is *z*-score distributions between markers from negative (purple) to positive (yellow). (F) Of those colonic sensory neurons assigned to subtypes based on postfunctional scqRT-PCR (see figure 5E), stacked bar charts showing the proportions in each identified subtype responding to capsaicin, MRS2365 and BIMU8. DRG, dorsal root ganglia; FB, Fast Blue; GDNF, glial-derived neurotrophic factor; 5-HT, 5-hydroxytryptamine; LS, lumbosacral; mNFa; mNeuroFilament-a; mNP; mNonPeptidergic; mPEPa; mPeptidergic-a; scRNAseq, single-cell RNAseq; TL, thoracolumbar.

Colonic sensory neurons respond to a vast range of stimuli. To investigate which subtypes were responding to differing stimuli we used Ca^2+^-imaging, coupled with post-imaging scqRT-PCR, to assign agonist responsiveness to the subtype framework (figure 5A). Specifically, we examined increases in [Ca^2+^]_i_ within colonic neurons in response to the algogenic compound capsaicin (Trpv1 agonist), the 5-HT_4_ receptor agonist BIMU8 and the P2Y_1_ receptor agonist MRS2365 (figure 5B). In contrast to *Trpv1*, both *Htr4* and *P2ry1* expressions are relatively restricted to mNP and mNFa subgroups, respectively (online supplementary figure 4B).

In agreement with previous studies,^28 29^ the algogenic Trpv1 agonist capsaicin activated 40% (50 of 125) of TL and 69% (29 of 42) of LS colonic neurons (figure 5C), reflecting the broad expression profile of *Trpv1* within mPEPa, mPEPb and pPEP subgroups, which collectively make up 57% of the sampled scRNAseq neurons (figure 5Di). The P2Y_1_ receptor agonist MRS2365 was used as an agonist biased towards activation of mNFa neurons (figure 5Dii), although it is worth noting that low-level expression was observed in other subgroups (eg, mNFb). In total, 14% (18 of 125) of TL and 21% (9 of 42) of LS neurons were sensitive to MRS2365. By contrast, expression of *Htr4* is much more restricted: all mNP colonic neurons have high expression of *Htr4*, a subset of mPEPb neurons possess low-level *Htr4* and it is poorly expressed or absent in the remaining subtypes (figure 5Diii). In agreement with this profile, 8% (10 of 125) of TL neurons responded to the addition of the 5-HT_4_ receptor agonist BIMU8, while no LS neurons (0 of 42) were activated. In broad terms, of the sensitive cells, four response profiles were observed in TL neurons: cells responsive to BIMU8 and capsaicin, but not MRS2365; cells responsive to MRS2365 and capsaicin, but not BIMU8; cells only responsive to MRS2365 and, finally, cells responsive solely to capsaicin. The almost perfect mutual exclusivity of responses to BIMU8 and MRS2365 mirrors the subtype-selective expression of *Htr4* (mNP) and *P2ry1* (mNFa) observed by scRNAseq (figure 5D). Furthermore, the large proportion (61%, 11 of 18) of MRS2365+ neurons that were also capsaicin-insensitive, fits well with the complete absence of *Trpv1* expression in mNFa neurons, with MRS2365+/capsaicin+ neurons likely examples of subgroups featuring low level expression of both *Htr4* and *P2ry1* (such as mNP, mNFb, mPEPa and pNF; figure 5D). It is possible that those LS neurons sensitive to both MRS2365 and capsaicin fall into this category.

After Ca^2+^-imaging, 62 FB-labelled colonic neurons were individually harvested and processed for scqRT-PCR. The relative expression of the seven marker genes (*Mrgprd, Cbln2*, *Fam19a1, Smr2*, *Trpa1*, *Hpse* and *Ntm*) was evaluated in 59 cells before assignment to subtypes (mNP, mNFa, mNFb, mPEPa, mPEPb, pNF and pPEP); cells predominantly expressing *Smr2* or *Ntm* (markers for mPEPa and pPEP, respectively) were not represented. Therefore, cells were clustered into five groups (mNP (n=17), mNFa (n=2), mNFb (n=7), mPEPb (n=29) and pNF (n=4); figure 5E) and functional responses mapped to this assignment. The absence of mPEPa and pPEP subtypes may be due to low sample size or an adverse sensitivity of these subtypes to extended primary culture required for Ca^2+^ imaging. Of those neurons sensitive to capsaicin, the majority (9 of 11 capsaicin+) mapped to the mPEPb subtype, in agreement with the scRNAseq analysis and suggestive of a nociceptive phenotype for this subgroup (figure 5F). MRS2365+ neurons were classified as mNP (n=2), mNFa (n=1) and mPEPb (n=2), with low frequency of the mNFa subgroup (only two neurons identified) constraining conclusions. Last, only two neurons harvested for scqRT-PCR were sensitive to BIMU8 and were linked to mPEPb subtype (figure 5F), which although not the subtype with highest expression, do possess low level *Htr4* (figure 5Diii). Using these stimuli, we show subtype-specific activation of colonic sensory neurons occurs and that such classification is a valid approach to assess the contribution of these pathways to visceral sensation.

## Discussion

Conscious sensation, including fullness, urge, discomfort and pain, is encoded by sensory neurons innervating the GI tract. In parallel, sensory pathways also coordinate complex defaecatory reflexes and act as an interface between luminal contents, the microbiota and the immune system. By regulating secretory processes, motility and blood flow, the extrinsic sensory innervation of the distal colon is capable of coordinating bowel function, detecting damage (eg, bacterial infiltration, ischaemia and inflammation) and regulating behaviour.

Visceral afferents are typically characterised by their sensitivity to mechanical and, to a lesser extent, chemical stimuli (eg, bradykinin, adenosine-5'-triphosphate (ATP) and capsaicin^30 31^) alongside anatomical criteria. However, a mechanosensitive categorisation paradigm overlooks other damage detecting transduction pathways that contribute to gut function. This is most clearly exemplified by those mechano-insensitive neurons or so-called silent afferents^31 32^ that do not sensitise to mechanical stimuli following incubation with inflammatory mediators and whose function is unknown. Indeed, how a recently identified population of visceral silent nociceptors (characterised by expression of nicotinic acetylcholine receptor subunit alpha 3 (*Chrna3*) and sensitisation by nerve growth factor) contributes to visceral sensation is unknown.^33^ The current lack of subtype-specific molecular markers for colonic sensory neurons constrains our understanding of reflex functions, conscious sensation from the bowel and in gut-specific pathologies (eg, IBS and IBD).

Here, we show using scRNAseq and unsupervised clustering that visceral sensory neurons innervating the mouse colorectum are populated by seven molecularly distinct subtypes (figure 1). Of these, only five subtypes are present in TL DRG neurons that project via the LSN and two subtypes are almost exclusive to LS DRG neurons that project via the PN, although examples of the remaining five are also present in a minority of LS DRG. Using this schema for classifying gut-projecting sensory neurons, we identified marker genes selectively expressed by each of the seven subtypes and validate the subtypes by scqRT-PCR and IHC; the utility of these marker genes for studying colonic sensory neurons in other species, including humans, will be the subject of future studies. The abundance of different subtypes in lumbar splanchnic (eg, mNP, mNFa, mNFb, mPEPa and mPEPb) versus pelvic (eg, mainly pNF and pPEP) innervation of the gut reflects the different stimuli transduced by these nerves and their functional requirements; for example, the encoding of defaecatory and evacuatory reflexes by pelvic, but not lumbar splanchnic, nerves.^34^

These data represent the first comprehensive single-cell transcriptomic profiling of sensory neurons of a known innervation target. Based on the expression patterns of genes contributing to sensory neuronal patterning, we identify one NP group, three NF groups and three PEP groups. Broadly our data match published datasets of L4-L6 non-identified DRG neurons but with some key differences.^16 35^ Nearly all colonic sensory neurons (just under 95%) express CGRP (encoded by *Calca*) in line with previous studies.^8 11 36^ Only mNP neurons completely lacked *Calca* and likely represent a subgroup of previously described isolectin B4-binding colonic neurons.^8 11^ While *Calca* was extensively expressed by PEP neuronal subtypes, low levels were observed in colonic neurons also expressing markers of NF subtypes (including *Nefh* and *Ldhb* and more specific markers such as *NECAB2*, *Fam19a1* and *Spp1*). Whereas cutaneous afferents often have conduction velocities (CVs) of up to 25 m/s,^37^ visceral sensory afferents rarely possess CVs exceeding 1 m/s in mouse.^32^ Therefore, the expression of NF markers is unlikely to correlate with significant myelination, but rather discriminates neurons into these two subtypes (PEP and NF). Whereas TL neurons broadly fit classifications outlined in published datasets, LS neurons do not. In the skin, expression of tyrosine hydroxylase (Th) defines C-fibre low-threshold mechanoreceptors (C-LTMR) in adult DRGs,^38^ with neurons expressing the non-peptidergic marker Gfrα2 and lacking CGRP, TrkA and Trpv1.^38^ In agreement with a previous study, LS subtypes express Th, with pPEP (*Th^high^*, online supplementary figure 4B) to a greater extent.^40^ By contrast, pNF neurons (with lower *Th* expression, *Th^low^*) abundantly express the mechanotransducer *Piezo2*,^37^ and pPEP *Th^high^* neurons express low levels of *Piezo2*, alongside high levels of *Calca*, *Ntrk1* and *Trpv1*, suggesting that *Th*+ neurons innervating the viscera do not in general accommodate the established somatic C-LTMR phenotype. Indeed, *ex vivo* recordings of L6 colonic neurons identify two distinct populations: one with low-firing frequencies and high thresholds to activation by distension of the bowel correlated to expression of Trpv1 and Gfrα3 and a second Trpv1/Gfrα3-negative group with high-firing frequencies and low-threshold activation.^28^ Our LS data directly support this observation, suggesting that pPEP neurons expressing *Trpv1* and *Gfra3* (and *Th^high^*) could represent a group of high-threshold nociceptors, while pNF neurons (negative for *Gfra3* and *Th^low^*) are low-threshold mechanotransducers likely contributing to defecation reflexes. Additionally, it is tempting to speculate whether the split of LS neurons into pPEP and mPEP groups represents observed differences in stretch-response functions of PN afferents terminating in the colon versus the rectum.^40^

Our data also provide insight into the sensory mechanisms contributing to the clinical efficacy of a class of drugs modulating serotonergic signalling. 5-HT receptor subunits possess selective expression within colonic neuronal subtypes. For example, alosetron, which reduces abdominal pain and discomfort in IBS, is a potent antagonist of 5-HT_3_ receptors that are expressed by NF neurons^41^ and likely represent sensory pathways coupled to EC chemosensors in the mucosa.^26^ *Htr4*, which encodes the 5-HT_4_ receptor, is only expressed by mNP neurons, implicating these as effectors of tegaserod, a partial 5-HT_4_ agonist, with disease-modifying properties in IBS.^42^ Other neuronal subtypes appear tuned to specific serotonergic signalling pathways (eg, mNFa neurons express *Htr1d*/*Htr7*, mNFa/mNFb express *Htr2a* and mPEPa/mPEPb/mNFb express *Htr1b*) advocating the pharmacological discrimination of visceral sensory pathways.

In conjunction with mechanosensation, the detection of damage-causing irritants, ischaemia and bacterial infiltration of the mucosal barrier represent critical sensory modalities for the gut-brain axis. Based on our analysis of the colonic neuronal subtypes present, we divide these three modalities by overlapping groups of neuronal subtypes. First, *Trpa1*/*Trpv1*-positive neurons (eg, mPEPb/mNP/pPEP) represent an irritant detection pathway (analogous to the somatic ‘itch’ pathway); activation of Trp-sensitive fibres evokes visceral hypersensitivity in mice.^22^ Release of histamine from intestinal mast cells can facilitate this sensitisation in IBS,^43^ which can be attenuated by Trpv1 antagonists in rodent models.^44^ Coexpression of *Mrgprd* and *Trpa1* by mNP neurons suggests that this subtype may represent a histamine-independent ‘itch’ pathway analogous to somatic systems.^45 46^ While it is not possible to ‘scratch’ the gut, facilitation of irritant removal by secretomotor events may be a consequence of activating this pathway. Interestingly, the G-protein coupled receptor PAR2 was almost not detected in any colonic sensory neuron by RNAseq or qRT-PCR, suggesting either challenges in detecting mRNA for this protein or alternative sites of action contributing to visceral hypersensitivity.

Mesenteric ischaemia is associated with severe abdominal pain, with potentially lethal peritonitis and septicaemia developing once transmural progression occurs.^47^ Less severe ischaemic colitis can also develop following a transient reduction in bloodflow. Asic3 acts as both an acid/lactate/ATP coincidence detector^48^ and a mechanosensor capable of initiating vasodilatory cascades against low local pressure in the skin.^49^ As such, Asic3 sensitive pathways (eg, mNFb/mPEPa neurons) may represent ischaemic sensing in the bowel and be important in the promotion of vasodilation.

Sensory nociceptors are sensitive to bacteria.^50^ In the gut, a high proportion of commensal bacteria are flagellated, and flagellin is a major antigen for the innate immune system. Bacterial invasion of the mucosal epithelium following altered gut permeability represents a significant breakdown of mucosal barrier function. Indeed, antibodies against flagellin are increased in IBS^51^ and Toll-like receptor 5 (Tlr5; the receptor for flagellin) signalling is impaired in Crohn’s disease.^52^ We find that mNFa neurons express *Tlr5* and mPEPa neurons express *Tlr4* (a receptor for the bacterial product lipopolysaccharide), suggesting that afferents of these subtypes contribute to defence mechanisms (including facilitating immune response) against bacterial penetration of the mucosal barrier.

In summary, these data provide a comprehensive framework for the molecular basis of visceral sensation originating from the lower GI tract. Additionally, they afford insight into the functional division of sensory modalities unique to the metabolic homeostasis of the bowel. Single-cell transcriptomic profiles enable the use of strategies to elucidate the functional roles of these neuronal subtypes, greatly facilitating our understanding of visceral sensory pathways in GI physiology in health and disease.
