# Supplementary material for: Single-cell RNAseq reveals seven classes of colonic sensory neuron
Source: Gut. 2018 Feb 26;68(4):633–44. doi: 10.1136/gutjnl-2017-315631 (PMC6580772; doi:10.1136/gutjnl-2017-315631)
Supplement: Supplementary data [file gutjnl-2017-315631supp001.pdf]

Single-Cell RNAseq Reveals Seven Classes of Colonic Sensory Neurone

Short Title

Colonic sensory neurone transcriptomics

James R.F. Hockley[1,2], Toni S. Taylor[1], Gerard Callejo[1], Anna L. Wilbrey[2], Alex Gutteridge[2], Karsten Bach[1], Wendy J. Winchester[2], David C. Bulmer[1], Gordon McMurray[2] and Ewan St. J. Smith[1]

Affiliations

[1] Department of Pharmacology, University of Cambridge, Tennis Court Road, Cambridge CB1 2PD, UK

[2] Pfizer Neuroscience and Pain Research Unit, Great Abington, Cambridge, UK

Co-corresponding authors

James R.F. Hockley PhD,
Department of Pharmacology, University of Cambridge, Tennis Court Road, Cambridge CB1 2PD, UK;
Email: jh2033@cam.ac.uk; Tel: +44 (0) 1223 339683

Ewan St. John Smith PhD,
Department of Pharmacology, University of Cambridge, Tennis Court Road, Cambridge CB1 2PD, UK;
Email: es336@cam.ac.uk; Tel: +44 (0) 1223 334048

Online Methods

Animals/ethics

C57Bl6/Jax mice were used for all experiments (see experimental subheadings for age and sex details). Mice were housed in a temperature controlled (21 ºC) room on a 12-hour light/dark cycle, with access to food and water *ad libitum* in groups of up to five. All protocols were performed in accordance with the UK Animals (Scientific Procedures) Act 1986 Amendment Regulations 2012 following ethical review by the University of Cambridge Animal Welfare and Ethical Review Body.

Labelling of colon-specific sensory neurones by retrograde tracer injection

Sensory neurones specifically innervating the distal colon *via* both splanchnic and pelvic nerves were labelled using the retrograde tracer Fast Blue (FB; 2 % in saline, Polysciences Gmbh, Germany) for expression analysis and Ca^{2+}-imaging experiments as previously described[11, 12]. Mice were anaesthetised with isoflurane (4 % induction, 1.25 - 1.5 % maintenance) and a midline laparotomy (~1.5 cm incision) was performed. Using a fine pulled borosilicate glass needle and microinfusion pump (0.4 µl min^{-1}), multiple bilateral injections of 0.2 µl FB were made into the distal colon over a region covering afferent receptive fields for both the splanchnic and pelvic pathways. The abdominal cavity was flushed with saline to remove any excess FB prior to securing muscle and skin by suturing and use of Michel clips. Animals were allowed to recover, during which time body weight was monitored and access to a soft, glucose-enriched diet and post-operative analgesia (buprenorphine 0.05-0.1 mg kg^{-1}) was provided.

Primary culture and cell picking

Three to ten days after surgery, colon-innervating thoracolumbar (TL; T10-L1) and lumbosacral (LS; L5-S2) dorsal root ganglia (DRG) were dissected and dissociated as two separate primary cultures using previously published protocols[12]. Dissected ganglia were trimmed of excess nerve fibre, the meninges removed and then incubated in Lebovitz L-15 Glutamax (Thermo Fisher Scientific, UK) media containing

1 mg ml^{-1} collagenase type 1A (Sigma-Aldrich, UK) and 6 mg ml^{-1} bovine serum albumin (BSA, Sigma-Aldrich) for 15 min at 37 °C (in 5 % CO_2), followed by L-15 media containing 1 mg ml^{-1} trypsin (Sigma-Aldrich) and 6 mg ml^{-1} BSA for 30 min. Gentle trituration of the ganglia was performed with a 1 mL Gilson pipette prior to collection, by brief centrifugation at 500 *g,* of the dissociated cell-containing supernatant and the trituration/centrifugation cycle repeated five times. Neurones from both TL and LS DRG were plated onto poly-D-lysine-coated coverslips (BD Biosciences, UK) and incubated (37 ºC in 5 % CO_2) in Lebovitz L-15 Glutamax media containing 2 % penicillin/streptomycin, 24 mM $NaHCO_3$, 38 mM glucose and 10 % fetal bovine serum. FB-positive colonic sensory neurones were identified by 365nm fluorescence illumination (Cairn Research, UK) and individual neurones manually harvested on an adapted inverted Olympus microscope using a micromanipulator controlled (PatchStar, Scientifica, UK) pulled glass pipette. Neurones were visually inspected prior to picking and only those free from debris and obviously not associated with satellite glia cells were captured and photographed (DCC1545M, ThorLabs Inc) for cell size analysis (images from 6 neurones were not captured). For single-cell RNAseq experiments, a maximum of 60 neurones per culture, per mouse were collected in an unbiased fashion within 6 hours of plating to minimise the potential for changes in gene expression. Negative controls were also obtained by collecting a small volume of bath solution; these were subjected to exactly the same protocols as samples containing isolated individual neurones. Labelled neurones were selected at random.

Single-cell RNAseq

In total 412 individual reaction tubes were collected consisting of 399 colonic sensory neurones from 10 mice (10-12 wks old, male) and 13 negative controls. Full-length cDNA from polyadenylated RNA of single cells was generated using previously published Smart-seq2 protocols[13]. In brief, isolated cells were collected into 4 µl single-cell RNAseq lysis buffer (consisting of 0.1 µl RNase inhibitor (Clontech), 1.9 µl 0.2 % Triton X-100, 1 µl oligo-dT$_{30}$VN primer and 1 µl dNTP mix (Fermentas, UK)) before brief centrifugation and immediate freezing on dry-ice. Whilst maintaining tubes on ice, cells were lysed by vortexing, centrifuged and the oligo-dT primer hybridised to the poly(A) tail of all mRNA molecules (72 °C for 3 min). Reverse transcription (RT) was performed by addition of 5.7 µl of RT mix (consisting of 0.5 µl Superscript II reverse

transcriptase (200 U μl^{-1}; Invitrogen), 0.25 μl RNase inhibitor (40 U μl^{-1}; Clontech), 2 μl Superscript II first-strand buffer (5x; Invitrogen), 0.5 μl DTT (100mM; Invitrogen), 2 μl betaine (5M; Sigma-Aldrich), 0.06 μl $MgCl_2$ (1M; Sigma-Aldrich), 0.1 μl template-switching oligo (TSO; 100 μM) and 0.29 μl nuclease-free water) per reaction and immediately subjected to thermal cycling (42 °C for 90 min then 10 cycles of (50 °C for 2 min, 42 °C for 2 min) and 70 °C for 15 min). To each reaction, 15 μl of PCR preamplification mix was added (consisting of 10 μl first-strand reaction (Invitrogen), 12.50 μl KAPA HiFi HotStart ReadyMix (2x; KAPA Biosystems), 0.25 μl IS PCR primers (10 μM) and 2.25 μl nuclease-free water) and the following PCR performed (98 °C for 3 min, then 21 cycles of (98 °C for 20 sec, 67 °C for 15 sec, 72 °C for 6 min) followed by 72 °C for 5 min). PCR reaction products were purified by incubation with 25 μl AMPure XP beads (Agencourt, Beckman Coulter) for 8 min, followed by magnetic retention, removal of the liquid and 2x wash in 200 μl of 80% (v/v) ethanol. After air drying, 17.5 μl of 10mM Tris-HCl, pH8.5 (TE) buffer was added to elute the DNA from the beads and transferred to a fresh 96-well plate. Individual samples were quantified using Quant-It PicoGreen dsDNA Assay (Molecular Probes) and analysed on a SpectraMax Plus spectrophotometer (Molecular Devices, USA) before dilution to 0.1 – 0.3 ng/μl in TE buffer.

Libraries were prepared using the Illumina Nextera XT DNA Sample Kit (Illumina) following the manufacturer's instructions. To 1.25 μl of each sample, 2.5 μl tagment DNA buffer and 1.25 μl amplification tagment mix were added, vortexed, centrifuged (4,000 rpm for 5 min) and tagmentation reaction performed on a thermal cycler (55 °C for 10 min). Once cooled, NT buffer (1.25 μl) was added to each tagmented sample to neutralise, plates vortexed and centrifuged (4,000 rpm for 5 min). Samples were 96-way multiplexed into 6 pooled libraries each containing a maximum of 96 reaction products. 3.75 μl of Nextera PCR Master Mix (NPM) was added to each sample in a 96-well plate, in addition to 1.25 μl of Index 1 Adapters (i7: N701, TAAGGCGA; N702, CGTACTAG; N703, AGGCAGAA; N704, TCCTGAGC; N705, GGACTCCT; N706, TAGGCATG; N707, CTCTCTAC; N708, CAGAGAGG; N709, GCTACGCT; N710, CGAGGCTG; N711, AAGAGGCA; N712, GTAGAGGA) row-wise and 1.25 μl of Index 2 Adapters (i5: S501, GCGATCTA (or S517, TCTTACGC); S502, ATAGAGAG; S503, AGAGGATA; S504, TCTACTCT; S505, CTCCTTAC; S506, TATGCAGT; S507, TACTCCTT; S508, AGGCTTAG) column-wise. Samples were vortexed, centrifuged

(4,000 rpm for 2 min) and a PCR amplification performed on a thermal cycler (72 °C for 2 min, 95 °C for 30 sec, then 12 cycles of (95 °C for 10 sec, 55 °C of 30 sec, 72 °C for 60 sec) followed by 72 °C for 5 min). Libraries were then pooled (1 µl of each sample) and purified by addition of 90% of the equivalent volume of AMPure XP beads (e.g. for 96 samples; 96 µl total pooled volume and 87 µl AMPure XP beads), followed by ethanol wash and DNA elution (1 µl per pooled library) steps mentioned above. The purification step was repeated a second time before elution in 1.5 µl TE buffer per pooled library. Concentration and size distribution of pooled libraries were quantified by Qubit dsDNA High Sensitivity Assay Kit (Thermo Fisher Scientific), Agilent High Sensitivity dsDNA Bioanalyzer (Agilent) and KAPA Library Quantification assays (KAPA Biosystems). Individual libraries (1-3 ng/uL with a size distribution of 500-800 bp) were sequenced from both ends to a length of 75 bp on an Illumina NextSeq500 at a read depth of ~4.9 M reads/cell. Reads were demultiplexed and aligned by STAR (v2.4.1) to the mouse reference genome (GRCm38.p3; Ensembl version 80; retrieved 2015-05-27) and genomic features determined using featureCounts (v1.4.4). We filtered low-quality cells from the dataset based on a threshold for the number of genes detected (i.e. minimum 5000 genes/cell; median value for all samples of 9380, this value is comparable to an existing single-cell sensory neuron RNAseq study (~10,000 genes)[16] and where the proportion of reads aligning to mitochondrial RNA (mtRNA) was low (i.e. < mean ± 2*SD (~8.5%); see Fig. S1a), which suggests successful genomic reverse transcription. Finally, the Smart-seq2 method is weakly 3' biased due to 3' poly(A) tail hybridisation and as an additional quality control, normalised gene coverage was also assessed for 3' bias (i.e. median 5' to 3' bias > 0.27; Fig. S1b). Using the *scater* package for analysis of single-cell RNA sequencing data[14], data from 325 cells and 34,769 genes was standardised for gene length by converting to Transcript Per Million (TPM) and normalised using size factors as previously described[15] and log transformed for all subsequent analyses (log(TPM+1)).

Regressing out latent technical effects

The 325 cells passing quality control measures described above were processed in 6 experimental batches: 74 in batch 1, 75 in batch 2, 76 in batch 3, 48 in batch 4, 47 in batch 5 and 5 in batch 6. Representatives of each cell type based on subsequent *de novo* clustering were present in each batch (apart from the pilot batch (6) consisting

of only 5 samples). We observed a weak batch effect corresponding to this experimental design and conservatively removed this using linear regression modelling as described by McCarthy *et al.*[14], with the derived residual expression from the model used as a 'corrected' gene expression value.

Clustering analysis and removal of contaminating subpopulations

Sensory neurones in DRG are encapsulated by satellite glia that may have contaminated individual samples, as such high co-expression of four satellite glia marker genes (ATPase Na^+/K^+ transporting subunit $\beta 2$ (*Atp1b2*), brain-type fatty acid binding protein 7 (*Fabp7*), sclerostin domain containing 1 (*Sostdc1*) and tissue inhibitor of metalloproteinase 3 (*Timp3*)) were used as exclusion criteria[16]. To avoid potentially confounding our dataset, we conservatively excluded samples with high expression of these four satellite glia markers resulting in 314 cells being taken forward into downstream clustering (Fig. S2). Dimensionality reduction and *k*-means consensus clustering was performed using the single-cell consensus clustering (SC3) package[17]. Ubiquitous and rare genes (expression values less than two) present or absent in at least 94% of cells were filtered and the optimal number of clusters assessed based on visualisation of the consensus matrix (Fig. 1b) and the silhouette index. The most stable result (average silhouette index of 0.92) was achieved with $k = 7$ and Pearson correlation with PCA transformation (Fig. 1b). To investigate the robustness of the clustering, we performed down-sampling of cells and bootstrapping. We repeated the SC3 cluster analysis ten times with a random selection of 90% of the total cell population (282 cells) and determined the Adjusted RAND Index (ARI) for similarity between the cluster assignments derived from the full dataset and the down-sampled dataset (Fig. S3). Where applicable, data was visualised using t-distributed Stochastic Neighbour Embedding (t-SNE) based on the top 500 most highly variable genes[14]. The clusters were stable giving ARI of 0.94 ± 0.06 (mean ± S.D.), with the largest appreciable changes observable when down-sampling reduced the number of cells present in less frequent clusters. Using SC3, we identified 1887 marker genes capable of distinguishing individual clusters using broad selection criteria (area under the receiver operating characteristic (AUROC) of ≥ 0.6 and $P < 0.01$; Table S1); 709 of which were present using more conservative criteria (AUROC ≥ 0.85 and $P < 0.01$)[17]. Many of these had been previously reported as selective markers of sensory

neuronal subpopulations[16, 35], shown to have differential expression patterns in colonic sensory neurones[28] and several which have not previously been reported (Fig. 1e).

Single-cell qRT-PCR

The colonic sensory neurone populations determined by hierarchical clustering of the scRNAseq data were first validated using scqRT-PCR. Colonic sensory neurones were retrogradely-labelled, picked and the expression of mRNA transcripts for subpopulation-specific marker genes determined by qRT-PCR as previously described[10, 12]. Primary cultures of colon-innervating TL and LS neurones were generated as described above and FB-positive colonic sensory neurones individually harvested by pulled glass pipette. Each cell was collected into a tube containing 5 µl CellDirect 2 × reaction buffer (Invitrogen, UK), 2.5 µl 0.2 × primer–probe mix, 0.1 µl SUPERase-in (Ambion, TX, USA), 1.2 µl TE buffer (Applichem, Germany) and 0.2 µl Superscript III Reverse Transcriptase–Platinum Taq mix (Invitrogen, UK) and immediately frozen on dry ice. Reverse transcription and preamplification of cDNA was achieved by thermal cycling (50 °C for 30 min, 95 °C for 2 min, then 24 cycles of 95 °C for 15 s, 60 °C for 4 min). Samples were diluted 1:5 in TE buffer and Taqman qPCR assays were run for each gene of interest (Taqman Assay ID: Cbln2, Mm01261946_m1; Smr2, Mm00491149_m1; Fam19a1, Mm00805384_m1; Mrgprd, Mm04212994_m1; Trpa1, Mm01227437_m1; Hpse, Mm00461768_m1; Ntm, Mm00549431_m1; GAPDH, Mm99999915_g1; F2r, Mm00438851_m1; F2rl1, Mm00433160_m1; F2rl2, Mm00438852_m1; F2rl3, Mm00433161_g1; Applied Biosystems) using the following cycling protocol: 50 °C for 2 min, 95 °C for 10 min, then 40 cycles of 95 °C for 15 s, 60 °C for 1 min. All single-cell RT-PCR products expressed glyceraldehyde-3-phosphate dehydrogenase (GAPDH), which acted as an internal positive control and bath control samples were negative for all Taqman reactions. As described above, an image of each neurone was also captured to enable post-hoc soma size analysis. Relative expression of marker genes were normalised to GAPDH quantification cycles (CT) using $2^{-\quad CT}$ formula and a *z*-score distribution of marker gene expression within a neurone determined. Normalised expression data per neurone was grouped using *k*-means-based clustering (with euclidean distances and complete linkage agglomeration method) based on the expression of Mrgprd, Cbln2, Fam19a1, Smr2, Trpa1, Hpse and Ntm only. In total, 180 colonic sensory

neurones were collected (90 from TL DRG and 90 LS DRG) from 6 mice (4 male, 2 females, 10-12 wks). Of these, qPCR products were detected in 168 neurones, which were assigned to colonic sensory neuronal subpopulation based on the relative expression of the seven marker genes.

Immunohistochemistry

Mice receiving FB colonic injections as described above were euthanised (sodium pentobarbital; 200 mg kg^{-1} i.p.) and transcardially perfused with saline (0.9 %) followed by 4 % paraformaldehyde in phosphate-buffered saline (PFA in PBS; pH 7.4). Thoracolumbar (T13-L1) and lumbosacral (L6-S1) DRG were removed (5 mice, 10 - 12 wks, female), post-fixed (4 % PFA; 2 h) and cryoprotected in 30 % sucrose (w/v PBS) overnight before freezing in Optimum Cutting Temperature Compound (Tissue-Tek, UK). Sections (12 µm) were collected on a cryostat sequentially across 10 slides per DRG and subsequently treated to 1 h blocking in antibody diluent (1 % BSA, 5 % donkey serum and 0.2 % Triton X-100 in PBS). Primary antibodies were incubated overnight at 4 °C (goat anti-Tropomyosin receptor kinase C (TrkC, 1:500, AF1404, R&D Systems), goat anti-GDNF Family Receptor Alpha 2 (Gfrα2, 1:200, AF429, R&D Systems), goat anti-GDNF Family Receptor Alpha 3 (Gfrα3, 1:300, AF2645, R&D Systems), goat anti-Neuropillin-1 (Nrp1, 1:200, AF566, R&D Systems), goat anti-osteopontin (Spp1, 1:300, AF808, R&D Systems) and species-specific secondary antibodies conjugated to fluorophores (donkey anti-goat IgG-AF488 (A11055), donkey anti-rabbit IgG-AF568 (A10042, Life Technologies)) were applied at room temperature (RT) for 2 h. In control experiments, no labelling was observed where primary antibody was excluded. Sections were imaged and the relative intensities of reaction products after immunostaining were determined for all colon-labelled DRG neurones with visible nuclei from 2 DRG per spinal segment from 2-3 animals (ImageJ v1.51a, NIH).

Ca^{2+}-imaging and post-functional classification

Primary cultures of TL and LS colon-innervating sensory neurones were prepared as described above. Neurones were plated onto poly-D-lysine-coated coverslips (BD Biosciences, UK) and incubated (37 ºC in 5 % CO_2) in Lebovitz L-15 Glutamax media containing 2 % penicillin/streptomycin, 24 mM $NaHCO_3$, 38 mM glucose and 10 %

fetal bovine serum for up to 2 hours prior to incubation with the Ca^{2+}-sensitive dye Fluo4 (10 µM; Thermo Fischer Scientific) in extracellular solution (ECS, in mM: NaCl 140, KCl 4, $CaCl_2$ 2, $MgCl_2$ 1, HEPES 10, glucose 4; adjusted to pH7.4 with NaOH and 300-310 mOsm with sucrose) for 30 min at RT. Coverslips were then washed and transfered to an imaging chamber (RC-26, Warner Instruments, UK) on an inverted Nikon Eclipse T*i* microscope under continuous perfusion with ECS. To avoid changes in gene expression, experiments were carried out within 8 hours of culturing DRG neurones. Under fluorescent illumination (365nm LED; Cairn Research, UK), emission from FB labelled neurones was captured using a Zyla sCMOS camera (Andor, UK). Subsequent images of Fluo-4 fluorescence (excited using 470 nm LED; Cairn Research, UK) were captured every second for 60 sec with a 250 ms exposure time using Micro-Manager software (v1.4; NIH). After a 10 sec baseline, the test compound was applied by a gravity rapid exchange perfusion system directly over the field of view for 15 sec and the response to wash-out (remaining 35 sec) captured. Subsequent compounds were applied at 5 min intervals in random order. KCl (50 mM) was applied in replacement of ECS wash during the final recording as a non-specific depolarising stimulus. Individual FB-positive neurones that responded to KCl were harvested using pulled glass pipettes as described above. Isolated neurones (62 neurones from 3 mice, 12-13 wks, female) free from debris, other neurones and glia were transferred to reaction tubes with 9 µl pre-amplification mastermix containing Taqman primer-probes for *Cbln2*, *Mrgprd*, *Fam19a1*, *Smr2*, *Trpa1*, *Hpse*, *Ntm* and *GAPDH* before processing for single-cell qRT-PCR as described above.

Data handling and statistics

For each sensory neurone picked for RNAseq and qRT-PCR validation experiments, the diameter (d_1) at the widest point and the diameter perpendicular (d_2) was determined. The normalised soma diameter (d) was calculated with the following formula;

$$d = 2\sqrt{\frac{d_1 d_2}{4}}$$

For Ca^{2+}-imaging experiments, Region of Interests (ROIs) were drawn around all FB-labelled gut-projecting neurones and overlaid. Background illumination was subtracted from ROI relative intensities before normalising between pre-compound application baseline and peak change in relative intensity following application of 50 mM KCl ($\Delta F/F_{max}$). Cells not responding to 50 mM KCl were excluded (i.e. peak Δ relative intensity of < 100 fluorescent units) from subsequent Ca^{2+}-imaging analyses. Responses of >0.2 $\Delta F/F_{max}$ Ca^{2+} flux to compound addition (selective 5-HT_4 agonist BIMU8 (1 μM), P2Y1 agonist MRS2365 (10 nM), Trpv1 agonist capsaicin (1 μM) and vehicle control (1.2% (v/v) DMSO) during the 15 sec of application were assigned as positive responses. Cells responding to application of vehicle also typically responded indiscriminately to compound addition and were likely of poor health and excluded. Agonist responses were visualised using R and the ggplot2 package[18].

Drugs

Stock concentrations of BIMU8 (1 mM; water), MRS2365 (10 μM; water) were prepared as described and purchased from Tocris Biosciences. Capsaicin (100 μM; EtOH) was purchased from Sigma-Aldrich. All compounds were diluted to working concentration in ECS buffer on the day of experimentation.

Code Availability

Computer code used to generate results that are reported in this study are available from the corresponding author on reasonable request.

Data Availability

Sequence data that support the findings of this study have been deposited Gene Expression Omnibus under accession code GSE102962. The expression profile of a given gene within colonic neuronal subtypes is provided at http://hockley.shinyapps.io/ColonicRNAseq.
