# Supplementary figures and images for: Single-cell RNAseq reveals seven classes of colonic sensory neuron
Source: Gut. 2018 Feb 26;68(4):633–44. doi: 10.1136/gutjnl-2017-315631 (PMC6580772; doi:10.1136/gutjnl-2017-315631)

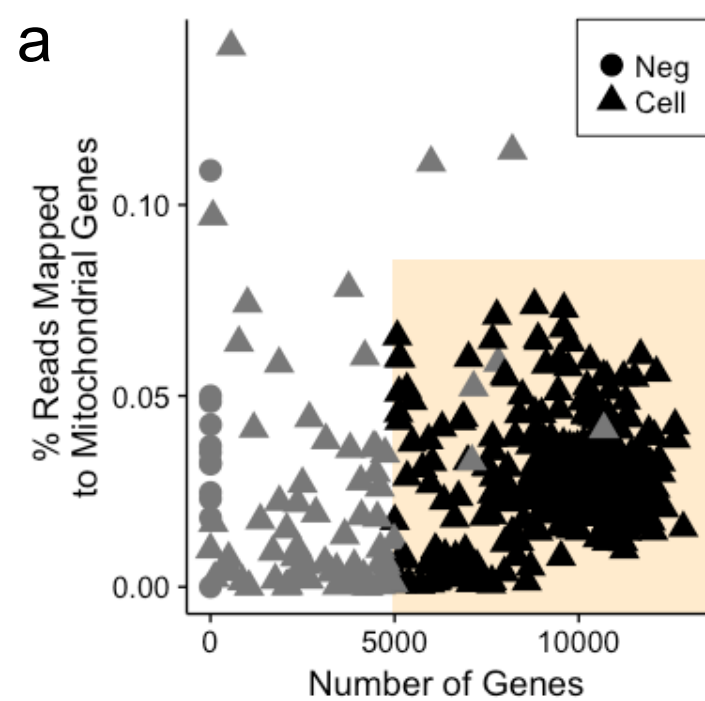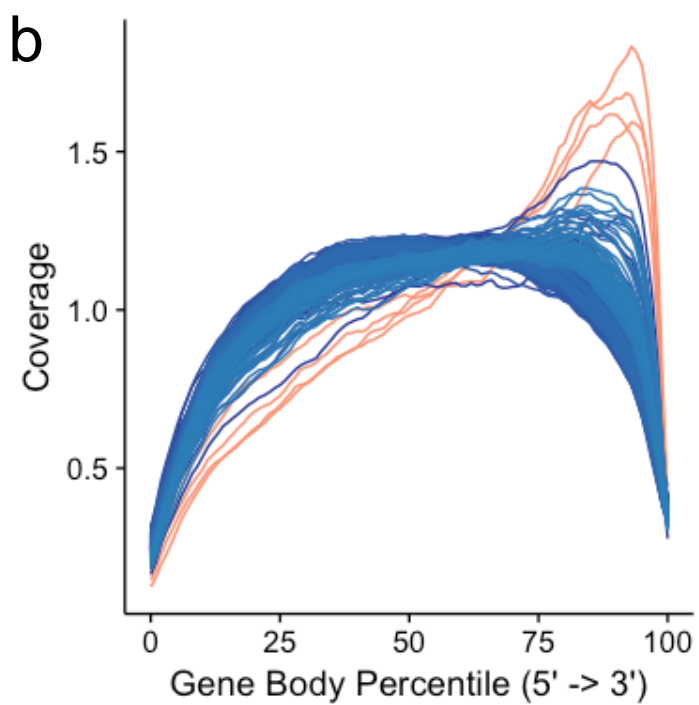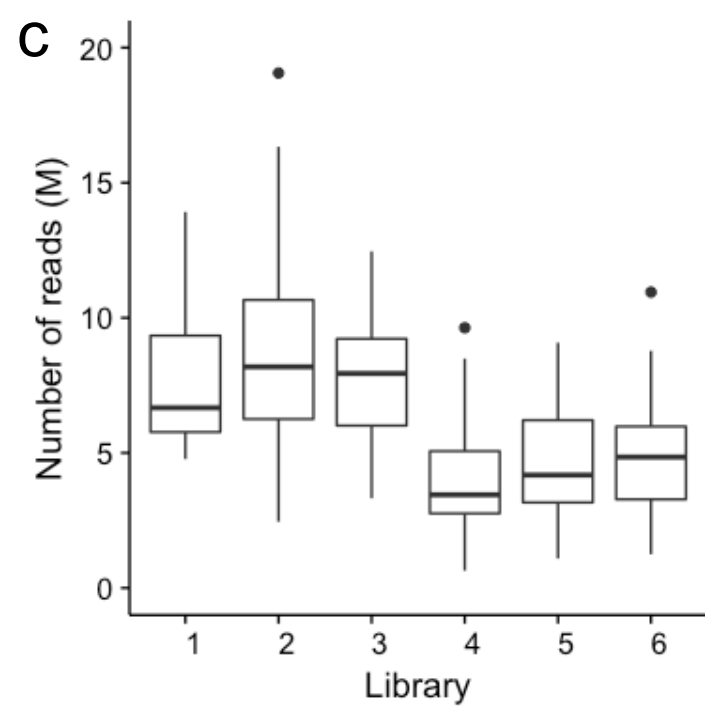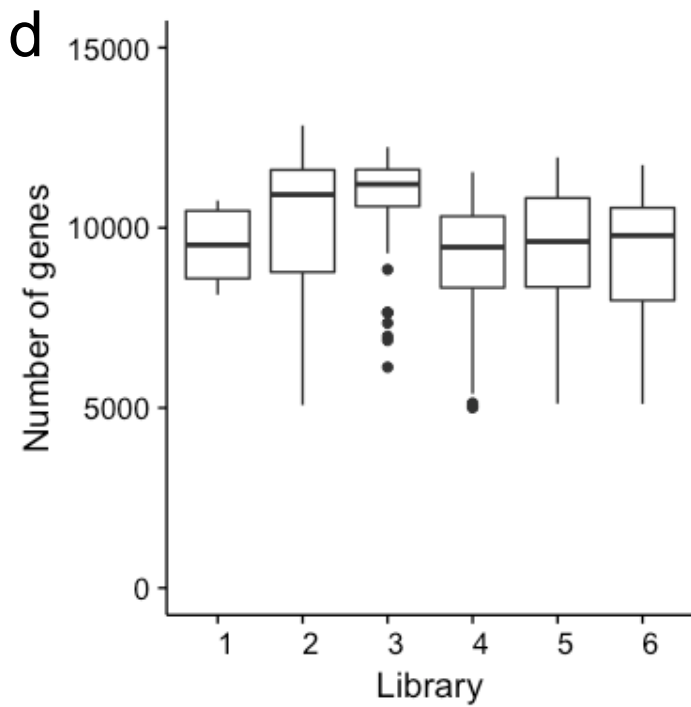

Supplement: Supplementary data [file gutjnl-2017-315631supp002.pdf]

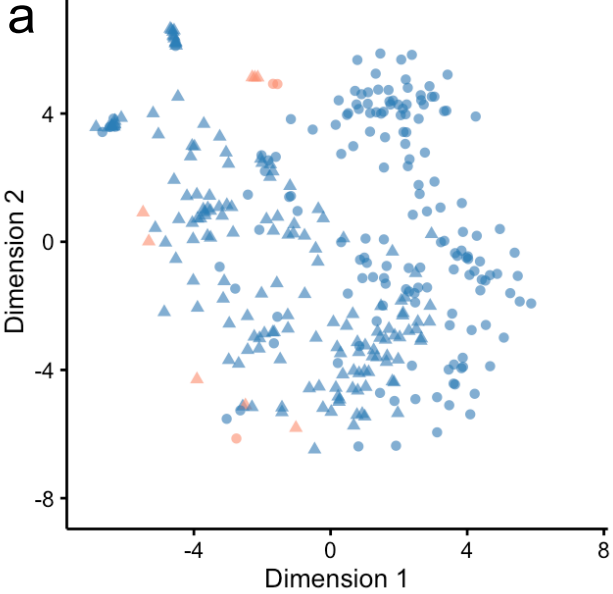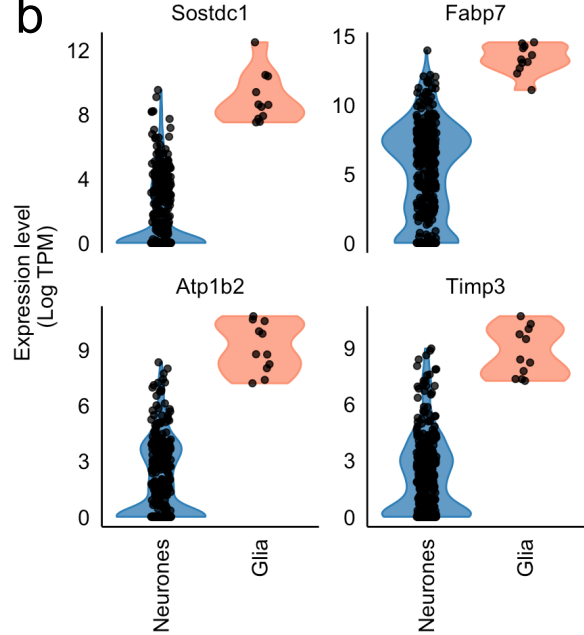

Supplement: Supplementary data [file gutjnl-2017-315631supp003.pdf]

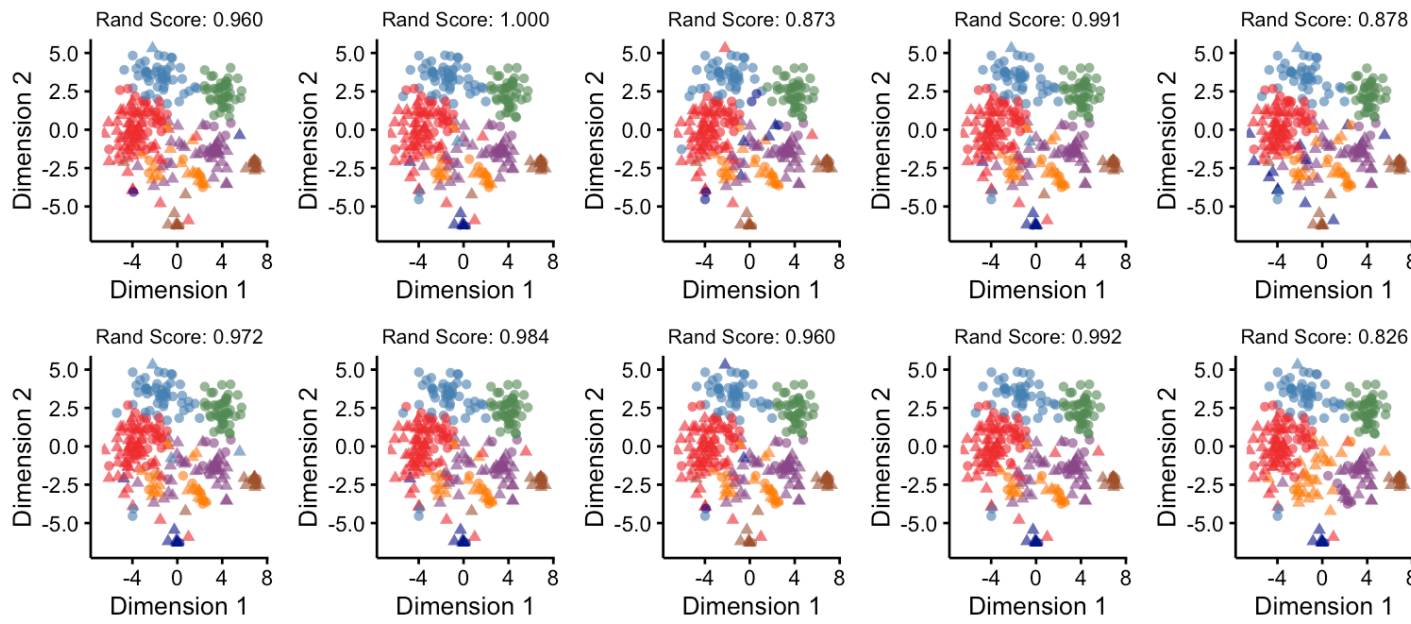

Supplement: Supplementary data [file gutjnl-2017-315631supp004.pdf]

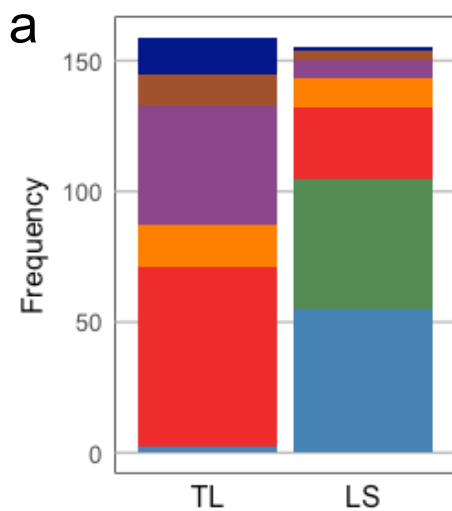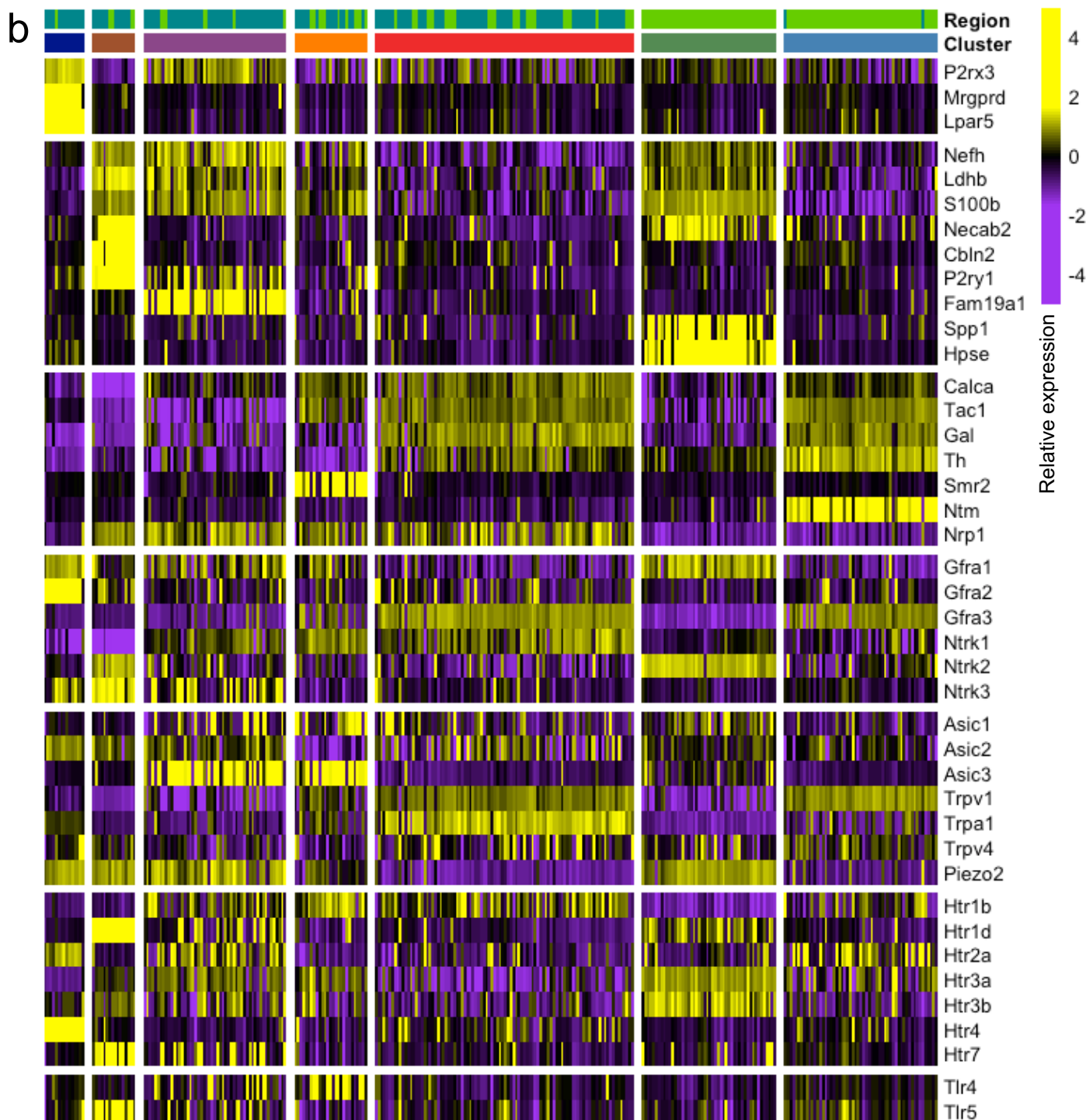

Supplement: Supplementary data [file gutjnl-2017-315631supp005.pdf]
