# Supplementary material for: Single-cell RNAseq reveals seven classes of colonic sensory neuron
Source: Gut. 2018 Feb 26;68(4):633–44. doi: 10.1136/gutjnl-2017-315631 (PMC6580772; doi:10.1136/gutjnl-2017-315631)
Supplement: Supplementary data [file gutjnl-2017-315631supp007.pdf]

Table S1

List of 1887 marker genes (ENSEMBL and Symbol) selectively expressed within specific clusters as determined by SC3 analysis (AUROC ≤ 0.6 and $P < 0.01$).

Cluster	ENSEMBL	Symbol	AUROC	p.value
mPEPb	ENSMUSG00000032769	Trpa1	0.92985474	4.83E-30
mPEPb	ENSMUSG00000039954	Stk32a	0.91057435	3.58E-27
mPEPb	ENSMUSG00000029420	Rimbp2	0.902331804	5.08E-26
mPEPb	ENSMUSG00000033717	Adra2a	0.900086009	8.86E-26
mPEPb	ENSMUSG00000047415	Gpr68	0.89012328	2.09E-24
mPEPb	ENSMUSG00000027004	Frzb	0.885440558	1.07E-23
mPEPb	ENSMUSG00000031492	Chrnb3	0.877006881	1.42E-22
mPEPb	ENSMUSG00000060429	Sntb1	0.873399274	4.01E-22
mPEPb	ENSMUSG00000046321	Hs3st2	0.872276376	5.67E-22
mPEPb	ENSMUSG00000031738	Irx6	0.862791476	7.28E-21
mPEPb	ENSMUSG00000015843	Rxrg	0.860784595	1.85E-20
mPEPb	ENSMUSG00000024366	Gfra3	0.860617355	1.73E-20
mPEPb	ENSMUSG00000062210	Tnfaip8	0.860426223	1.97E-20
mPEPb	ENSMUSG00000042846	Lrrtm3	0.860354549	2.02E-20
mPEPb	ENSMUSG00000067438	Hmx1	0.857224771	4.22E-20
mPEPb	ENSMUSG00000019775	Rgs17	0.85588685	7.64E-20
mPEPb	ENSMUSG00000063626	Unc5d	0.85106078	2.35E-19
mPEPb	ENSMUSG00000036528	Ppfibp2	0.846593081	1.04E-18
mPEPb	ENSMUSG00000021032	Ngb	0.841958142	3.79E-18
mPEPb	ENSMUSG00000026271	Gpr35	0.840142393	6.23E-18
mPEPb	ENSMUSG00000028359	Orm3	0.839330084	6.09E-18
mPEPb	ENSMUSG00000074345	Tnfaip8l3	0.838063838	8.84E-18
mPEPb	ENSMUSG00000021614	Vcan	0.83627198	1.60E-17
mPEPb	ENSMUSG00000018339	Gpx3	0.835626911	2.14E-17
mPEPb	ENSMUSG00000085931	NA	0.835053517	1.94E-17
mPEPb	ENSMUSG00000103346	NA	0.834527905	2.25E-17
mPEPb	ENSMUSG00000016758	Bik	0.834097859	2.52E-17
mPEPb	ENSMUSG00000067081	Asb18	0.833500573	3.50E-17
mPEPb	ENSMUSG00000024907	Gal	0.831899847	5.79E-17
mPEPb	ENSMUSG00000020520	Galnt10	0.831708716	6.13E-17
mPEPb	ENSMUSG00000035528	Npffr2	0.827408257	1.18E-16
mPEPb	ENSMUSG00000016024	Lbp	0.825903096	1.66E-16
mPEPb	ENSMUSG00000046834	Krt1	0.825879205	2.29E-16
mPEPb	ENSMUSG00000034336	Ina	0.825019113	3.57E-16
mPEPb	ENSMUSG00000034981	Parm1	0.823060015	6.01E-16
mPEPb	ENSMUSG00000048218	Amigo2	0.820766437	1.09E-15

mPEPb	ENSMUSG00000031364	Grpr	0.820694763	9.37E-16
mPEPb	ENSMUSG00000027584	Oprl1	0.819046254	1.60E-15
mPEPb	ENSMUSG00000019876	Pkib	0.817708333	2.39E-15
mPEPb	ENSMUSG00000039286	Fndc3b	0.817564985	2.47E-15
mPEPb	ENSMUSG00000032564	Cpne4	0.817445528	2.56E-15
mPEPb	ENSMUSG00000032776	Mctp2	0.816035933	3.48E-15
mPEPb	ENSMUSG00000047904	Sstr2	0.814650229	4.36E-15
mPEPb	ENSMUSG00000036502	Tmem255a	0.814459098	5.45E-15
mPEPb	ENSMUSG00000015829	Tnr	0.813814029	6.00E-15
mPEPb	ENSMUSG00000054976	Nyap2	0.813527332	6.18E-15
mPEPb	ENSMUSG00000004626	Stxbp2	0.812476109	8.29E-15
mPEPb	ENSMUSG00000024937	Ehbp1l1	0.811377102	1.19E-14
mPEPb	ENSMUSG00000022179	4931414P19Rik	0.811233754	1.13E-14
mPEPb	ENSMUSG00000074607	Tox2	0.810708142	1.39E-14
mPEPb	ENSMUSG00000066189	Cacng3	0.810325879	1.35E-14
mPEPb	ENSMUSG00000070552	Mrgprx1	0.809871942	1.25E-14
mPEPb	ENSMUSG00000040990	Sh3kbp1	0.809728593	1.80E-14
mPEPb	ENSMUSG00000052981	Ube2ql1	0.807578364	3.07E-14
mPEPb	ENSMUSG00000037624	Kcnk2	0.80733945	3.21E-14
mPEPb	ENSMUSG00000076612	NA	0.807172209	2.97E-14
mPEPb	ENSMUSG00000022945	Chaf1b	0.806622706	3.45E-14
mPEPb	ENSMUSG00000100850	NA	0.806240443	2.43E-14
mPEPb	ENSMUSG00000078670	Fam174b	0.805643157	4.95E-14
mPEPb	ENSMUSG00000036111	Lmo1	0.804759174	5.95E-14
mPEPb	ENSMUSG00000075325	Gm13582	0.8046875	5.36E-14
mPEPb	ENSMUSG00000037610	Kcnmb2	0.804329128	6.63E-14
mPEPb	ENSMUSG00000032437	Stt3b	0.803588494	8.20E-14
mPEPb	ENSMUSG00000050875	A730017C20Rik	0.802250573	1.14E-13
mPEPb	ENSMUSG00000028152	Tspan5	0.79986143	2.03E-13
mPEPb	ENSMUSG00000041479	Syt15	0.799311927	1.72E-13
mPEPb	ENSMUSG00000019139	Isyna1	0.798642966	2.72E-13
mPEPb	ENSMUSG00000037681	Esyt3	0.798069572	2.70E-13
mPEPb	ENSMUSG00000060735	Rxfp3	0.793697439	6.33E-13
mPEPb	ENSMUSG00000068762	Gstm6	0.793601873	8.75E-13
mPEPb	ENSMUSG00000032849	Abcc4	0.792980696	1.05E-12
mPEPb	ENSMUSG00000039364	Sectm1b	0.792096713	1.19E-12
mPEPb	ENSMUSG00000064294	Aox3	0.790734901	1.30E-12
mPEPb	ENSMUSG00000025888	Casp1	0.790304855	1.77E-12
mPEPb	ENSMUSG00000026688	Mgst3	0.789612003	2.32E-12
mPEPb	ENSMUSG00000047787	Flrt1	0.788584671	2.86E-12
mPEPb	ENSMUSG00000039629	Strip2	0.78789182	3.42E-12
mPEPb	ENSMUSG00000003559	As3mt	0.786840596	4.42E-12
mPEPb	ENSMUSG00000087196	NA	0.785598242	5.31E-12

mPEPb	ENSMUSG00000028713	Cyp4b1	0.78557435	4.52E-12
mPEPb	ENSMUSG00000022587	Ly6e	0.784857607	6.87E-12
mPEPb	ENSMUSG00000020844	Nxn	0.784762041	7.13E-12
mPEPb	ENSMUSG00000038248	Sobp	0.78457091	7.46E-12
mPEPb	ENSMUSG00000026389	Steap3	0.784523127	7.54E-12
mPEPb	ENSMUSG00000032038	St3gal4	0.78423643	8.00E-12
mPEPb	ENSMUSG00000053930	Shisa6	0.783830275	7.08E-12
mPEPb	ENSMUSG00000082193	NA	0.783686927	7.93E-12
mPEPb	ENSMUSG00000051515	NA	0.78158448	1.05E-11
mPEPb	ENSMUSG00000020098	Pcbd1	0.781536697	1.49E-11
mPEPb	ENSMUSG00000097796	Gm16702	0.780939411	1.69E-11
mPEPb	ENSMUSG00000009214	Tmem8c	0.780198777	1.60E-11
mPEPb	ENSMUSG00000031491	Chrna6	0.779505925	2.35E-11
mPEPb	ENSMUSG00000005124	Wisp1	0.779028096	2.54E-11
mPEPb	ENSMUSG00000002012	Pnck	0.77859805	2.90E-11
mPEPb	ENSMUSG00000051980	Casr	0.777905199	3.18E-11
mPEPb	ENSMUSG00000036856	Wnt4	0.777690176	3.27E-11
mPEPb	ENSMUSG00000026301	Iqca	0.777499044	2.49E-11
mPEPb	ENSMUSG00000031841	Cdh13	0.777475153	3.68E-11
mPEPb	ENSMUSG00000051359	Ncald	0.776877867	4.27E-11
mPEPb	ENSMUSG00000071234	Syndig1l	0.775922209	5.07E-11
mPEPb	ENSMUSG00000029361	Nos1	0.775516055	5.78E-11
mPEPb	ENSMUSG00000037492	Zmat4	0.775396598	5.84E-11
mPEPb	ENSMUSG00000037031	Tspan15	0.774823203	6.73E-11
mPEPb	ENSMUSG00000069855	Slc47a2	0.772816323	1.00E-10
mPEPb	ENSMUSG00000085915	NA	0.772792431	9.33E-11
mPEPb	ENSMUSG00000069072	Slc7a14	0.772696865	1.08E-10
mPEPb	ENSMUSG00000084973	Gm13848	0.771287271	1.25E-10
mPEPb	ENSMUSG00000022235	Cmbl	0.770164373	1.88E-10
mPEPb	ENSMUSG00000074749	Kiz	0.770140482	1.89E-10
mPEPb	ENSMUSG00000039007	Cpq	0.769017584	2.41E-10
mPEPb	ENSMUSG00000023367	Tmem176a	0.768706995	2.58E-10
mPEPb	ENSMUSG00000022856	Tmem41a	0.768348624	2.79E-10
mPEPb	ENSMUSG00000031488	Rab11fip1	0.767297401	3.01E-10
mPEPb	ENSMUSG00000058159	NA	0.767058486	3.17E-10
mPEPb	ENSMUSG00000010066	Cacna2d2	0.76679568	3.88E-10
mPEPb	ENSMUSG00000032109	Nlrx1	0.7664612	4.01E-10
mPEPb	ENSMUSG00000084126	NA	0.765744457	3.55E-10
mPEPb	ENSMUSG00000006219	Fblim1	0.765266628	4.99E-10
mPEPb	ENSMUSG00000016757	Ttll12	0.764263188	6.73E-10
mPEPb	ENSMUSG00000048644	Ctxn1	0.763044725	8.73E-10
mPEPb	ENSMUSG00000070867	Trabd2b	0.762996942	6.89E-10
mPEPb	ENSMUSG00000040183	Ankrd6	0.76180237	1.14E-09

mPEPb	ENSMUSG00000021541	Trpc7	0.761539564	1.04E-09
mPEPb	ENSMUSG00000000125	Wnt3	0.761372324	1.07E-09
mPEPb	ENSMUSG00000028100	Nudt17	0.761276758	1.26E-09
mPEPb	ENSMUSG00000086843	E030013I19Rik	0.760727255	7.26E-10
mPEPb	ENSMUSG00000031932	Gpr83	0.760583907	1.22E-09
mPEPb	ENSMUSG00000086804	NA	0.760129969	1.45E-09
mPEPb	ENSMUSG00000022773	Ypel1	0.759938838	1.67E-09
mPEPb	ENSMUSG00000043441	Gpr149	0.759891055	1.65E-09
mPEPb	ENSMUSG00000024664	Fads3	0.759508792	1.84E-09
mPEPb	ENSMUSG00000043068	Fam89a	0.759461009	1.86E-09
mPEPb	ENSMUSG00000015396	Cd83	0.758529243	1.84E-09
mPEPb	ENSMUSG00000002265	Peg3	0.758457569	2.29E-09
mPEPb	ENSMUSG00000031119	Gpc4	0.757931957	2.12E-09
mPEPb	ENSMUSG00000029153	Ociad2	0.757669151	2.64E-09
mPEPb	ENSMUSG00000023945	Slc5a7	0.75680906	3.08E-09
mPEPb	ENSMUSG00000041708	Mpped1	0.756785168	3.20E-09
mPEPb	ENSMUSG00000013846	St3gal1	0.756092317	3.55E-09
mPEPb	ENSMUSG00000028194	Ddah1	0.755925076	3.88E-09
mPEPb	ENSMUSG00000041654	Slc39a11	0.755733945	4.04E-09
mPEPb	ENSMUSG00000074782	4833422C13Rik	0.755662271	4.04E-09
mPEPb	ENSMUSG00000073535	NA	0.755064985	3.16E-09
mPEPb	ENSMUSG00000006930	Hap1	0.754372133	5.32E-09
mPEPb	ENSMUSG00000044288	Cnr1	0.754348242	5.37E-09
mPEPb	ENSMUSG00000004035	Gstm7	0.754348242	5.33E-09
mPEPb	ENSMUSG00000034773	BC030867	0.75415711	5.49E-09
mPEPb	ENSMUSG00000028661	Epha8	0.753344801	5.00E-09
mPEPb	ENSMUSG00000025468	Caly	0.753129778	6.90E-09
mPEPb	ENSMUSG00000029321	Slc10a6	0.753034213	6.96E-09
mPEPb	ENSMUSG00000021803	Cdhr1	0.750597286	1.15E-08
mPEPb	ENSMUSG00000085953	NA	0.749522171	1.40E-08
mPEPb	ENSMUSG00000015709	Arnt2	0.749307148	1.50E-08
mPEPb	ENSMUSG00000043635	Adamts3	0.749020451	1.32E-08
mPEPb	ENSMUSG00000021534	1700001L19Rik	0.748327599	1.82E-08
mPEPb	ENSMUSG00000039956	Mrap	0.748040902	1.71E-08
mPEPb	ENSMUSG00000024873	Cnih2	0.747371942	2.22E-08
mPEPb	ENSMUSG00000028064	Sema4a	0.747061353	2.29E-08
mPEPb	ENSMUSG00000030666	Calcb	0.746798547	2.49E-08
mPEPb	ENSMUSG00000032265	Fam46a	0.746487959	2.63E-08
mPEPb	ENSMUSG00000026360	Rgs2	0.746296827	2.74E-08
mPEPb	ENSMUSG00000028871	Rspo1	0.745580084	2.89E-08
mPEPb	ENSMUSG00000074006	Omp	0.745221713	3.21E-08
mPEPb	ENSMUSG00000026976	Pax8	0.744719992	3.55E-08
mPEPb	ENSMUSG00000026170	Cyp27a1	0.744385512	2.97E-08

mPEPb	ENSMUSG00000026463	Atp2b4	0.744313838	4.06E-08
mPEPb	ENSMUSG00000045216	Hs6st1	0.743979358	4.36E-08
mPEPb	ENSMUSG00000028517	Plpp3	0.743597095	4.68E-08
mPEPb	ENSMUSG00000032297	Celf6	0.743167049	5.09E-08
mPEPb	ENSMUSG00000037362	Nov	0.741972477	5.51E-08
mPEPb	ENSMUSG00000078235	Fam43b	0.740969037	7.87E-08
mPEPb	ENSMUSG00000092392	NA	0.740515099	7.45E-08
mPEPb	ENSMUSG00000026090	2010300C02Rik	0.740491208	8.31E-08
mPEPb	ENSMUSG00000000766	Oprm1	0.740228402	9.04E-08
mPEPb	ENSMUSG00000094910	D430019H16Rik	0.739272745	1.09E-07
mPEPb	ENSMUSG00000050071	Bex1	0.737456995	1.55E-07
mPEPb	ENSMUSG00000021340	Gpld1	0.736955275	1.71E-07
mPEPb	ENSMUSG00000030102	Itpr1	0.736788035	1.72E-07
mPEPb	ENSMUSG00000010663	Fads1	0.736764144	1.78E-07
mPEPb	ENSMUSG00000020846	Fam101b	0.735474006	2.27E-07
mPEPb	ENSMUSG00000029832	Nfe2l3	0.73537844	2.03E-07
mPEPb	ENSMUSG00000025743	Sdc3	0.735258983	2.37E-07
mPEPb	ENSMUSG00000039166	Akap7	0.734470566	2.75E-07
mPEPb	ENSMUSG00000042115	Klhdc8a	0.732965405	3.66E-07
mPEPb	ENSMUSG00000106603	NA	0.732272554	3.97E-07
mPEPb	ENSMUSG00000085981	NA	0.732200879	3.20E-07
mPEPb	ENSMUSG00000038668	Lpar1	0.732033639	4.34E-07
mPEPb	ENSMUSG00000028347	Tmeff1	0.731460245	4.83E-07
mPEPb	ENSMUSG00000056394	Lig1	0.731006307	5.28E-07
mPEPb	ENSMUSG00000019846	Lama4	0.731006307	5.23E-07
mPEPb	ENSMUSG00000023439	Gnb3	0.730862959	5.07E-07
mPEPb	ENSMUSG00000042761	Mrap2	0.729763953	6.63E-07
mPEPb	ENSMUSG00000026773	Pfkfb3	0.729525038	6.96E-07
mPEPb	ENSMUSG00000024268	Celf4	0.729501147	6.99E-07
mPEPb	ENSMUSG00000019359	Gdpd2	0.728664946	7.53E-07
mPEPb	ENSMUSG00000087131	NA	0.728091552	8.90E-07
mPEPb	ENSMUSG00000021451	Sema4d	0.727446483	1.02E-06
mPEPb	ENSMUSG00000016503	Gtf3a	0.726036888	1.32E-06
mPEPb	ENSMUSG00000074071	Fam169b	0.725487385	1.34E-06
mPEPb	ENSMUSG00000076431	Sox4	0.724842317	1.64E-06
mPEPb	ENSMUSG00000055409	Nell1	0.724794534	1.29E-06
mPEPb	ENSMUSG00000061911	Myt1l	0.724340596	1.80E-06
mPEPb	ENSMUSG00000048058	Ldlrad3	0.724197248	1.82E-06
mPEPb	ENSMUSG00000049744	Arhgap15	0.724125573	1.87E-06
mPEPb	ENSMUSG00000022240	Ctnnd2	0.723886659	1.96E-06
mPEPb	ENSMUSG00000029477	Morn3	0.723169916	1.84E-06
mPEPb	ENSMUSG00000020646	Mboat2	0.722859327	2.33E-06
mPEPb	ENSMUSG00000075012	Fjx1	0.722715979	1.83E-06

mPEPb	ENSMUSG00000018819	Lsp1	0.722333716	2.37E-06
mPEPb	ENSMUSG00000017144	Rnd3	0.722094801	2.70E-06
mPEPb	ENSMUSG00000045246	Kcng4	0.721354167	2.28E-06
mPEPb	ENSMUSG00000054196	Cthrc1	0.720995795	3.18E-06
mPEPb	ENSMUSG00000060002	Chpt1	0.720971904	3.30E-06
mPEPb	ENSMUSG00000028370	Pappa	0.720661315	3.32E-06
mPEPb	ENSMUSG00000028173	Wls	0.720541858	3.57E-06
mPEPb	ENSMUSG00000039046	Usp6nl	0.720279052	3.74E-06
mPEPb	ENSMUSG00000035681	Kcnc2	0.720231269	3.77E-06
mPEPb	ENSMUSG00000004110	Cacna1e	0.720207378	3.75E-06
mPEPb	ENSMUSG00000062561	NA	0.71992068	2.82E-06
mPEPb	ENSMUSG00000039963	Ccdc40	0.719395069	4.37E-06
mPEPb	ENSMUSG00000097094	NA	0.719180046	3.57E-06
mPEPb	ENSMUSG00000069792	Wfdc17	0.719108372	4.00E-06
mPEPb	ENSMUSG00000021319	Sfrp4	0.718988914	4.63E-06
mPEPb	ENSMUSG00000029352	Crybb3	0.717770451	5.49E-06
mPEPb	ENSMUSG00000095079	NA	0.717149274	5.05E-06
mPEPb	ENSMUSG00000022876	Samsn1	0.716862576	6.82E-06
mPEPb	ENSMUSG00000032890	Rims3	0.716265291	7.59E-06
mPEPb	ENSMUSG00000019851	Perp	0.715978593	7.96E-06
mPEPb	ENSMUSG00000086152	NA	0.715309633	6.40E-06
mPEPb	ENSMUSG00000090061	Nwd2	0.714473433	1.04E-05
mPEPb	ENSMUSG00000007613	Tgfbr1	0.714282301	1.06E-05
mPEPb	ENSMUSG00000020793	Galr2	0.713995604	8.47E-06
mPEPb	ENSMUSG00000020644	Id2	0.713732798	1.18E-05
mPEPb	ENSMUSG00000009687	Fxyd5	0.712227638	1.51E-05
mPEPb	ENSMUSG00000029095	Ablim2	0.71210818	1.56E-05
mPEPb	ENSMUSG00000044345	Marveld1	0.712084289	1.55E-05
mPEPb	ENSMUSG00000025175	Fn3k	0.711869266	1.62E-05
mPEPb	ENSMUSG00000034591	Slc41a2	0.711821483	1.64E-05
mPEPb	ENSMUSG00000091430	NA	0.7117737	1.36E-05
mPEPb	ENSMUSG00000066235	Pomgnt2	0.711702026	1.67E-05
mPEPb	ENSMUSG00000006800	Sulf2	0.711654243	1.55E-05
mPEPb	ENSMUSG00000001988	Npas1	0.711654243	1.59E-05
mPEPb	ENSMUSG00000020703	5530401A14Rik	0.710841934	1.53E-05
mPEPb	ENSMUSG00000020023	Tmcc3	0.710340214	2.11E-05
mPEPb	ENSMUSG00000032491	Nradd	0.710172974	2.10E-05
mPEPb	ENSMUSG00000029090	Adgra3	0.710125191	2.15E-05
mPEPb	ENSMUSG00000013653	1810065E05Rik	0.709957951	1.80E-05
mPEPb	ENSMUSG00000003341	Atp8b3	0.709480122	2.30E-05
mPEPb	ENSMUSG00000005705	Agrp	0.709384557	2.35E-05
mPEPb	ENSMUSG00000051107	NA	0.708405008	2.92E-05
mPEPb	ENSMUSG00000103538	NA	0.708142202	1.95E-05

mPEPb	ENSMUSG00000016763	Scube1	0.708142202	2.79E-05
mPEPb	ENSMUSG00000066877	Nck2	0.707353784	3.38E-05
mPEPb	ENSMUSG00000086885	NA	0.707258219	2.79E-05
mPEPb	ENSMUSG00000051166	Eml5	0.707067087	3.66E-05
mPEPb	ENSMUSG00000079564	NA	0.70594419	3.72E-05
mPEPb	ENSMUSG00000026321	Tnfrsf11a	0.705920298	4.38E-05
mPEPb	ENSMUSG00000022421	Nptxr	0.703937309	6.18E-05
mPEPb	ENSMUSG00000022261	Sdc2	0.703865635	6.24E-05
mPEPb	ENSMUSG00000071724	Smpd5	0.703722286	6.09E-05
mPEPb	ENSMUSG00000025986	Slc39a10	0.703674503	6.44E-05
mPEPb	ENSMUSG00000035200	Chrnb4	0.703650612	6.42E-05
mPEPb	ENSMUSG00000022840	Adcy5	0.703507263	6.60E-05
mPEPb	ENSMUSG00000021621	Zcchc9	0.70262328	7.66E-05
mPEPb	ENSMUSG00000031972	Acta1	0.701930428	8.55E-05
mPEPb	ENSMUSG00000015222	Map2	0.701452599	9.28E-05
mPEPb	ENSMUSG00000053214	Gm9899	0.701046445	9.61E-05
mPEPb	ENSMUSG00000066800	Rnasel	0.699995222	0.000117543
mPEPb	ENSMUSG00000033585	Ndn	0.698681193	0.000145425
mPEPb	ENSMUSG00000020176	Grb10	0.698107798	0.000159195
mPEPb	ENSMUSG00000000223	Drp2	0.697868884	0.000164889
mPEPb	ENSMUSG00000025551	Fgf14	0.697749427	0.000139216
mPEPb	ENSMUSG00000017721	Pigt	0.697653861	0.000171181
mPEPb	ENSMUSG00000031842	Pde4c	0.697438838	0.000171185
mPEPb	ENSMUSG00000072553	Gm525	0.697271598	0.000144767
mPEPb	ENSMUSG00000033256	Shf	0.696913226	0.000191924
mPEPb	ENSMUSG00000041992	Rapgef5	0.696865443	0.000191086
mPEPb	ENSMUSG00000042816	Gpr151	0.696865443	0.00018371
mPEPb	ENSMUSG00000027276	Jag1	0.696793769	0.000194904
mPEPb	ENSMUSG00000070817	Vmn1r85	0.696793769	0.000131014
mPEPb	ENSMUSG00000087074	NA	0.695933677	0.000176542
mPEPb	ENSMUSG00000031838	Ifi30	0.695193043	0.000252734
mPEPb	ENSMUSG00000040536	Necab1	0.695073586	0.000258528
mPEPb	ENSMUSG00000021185	9030617O03Rik	0.69497802	0.000261741
mPEPb	ENSMUSG00000054863	Fam19a5	0.694739106	0.000271777
mPEPb	ENSMUSG00000050830	Vwc2	0.694667431	0.000255067
mPEPb	ENSMUSG00000021876	Rnase4	0.6944763	0.000282524
mPEPb	ENSMUSG00000037336	Mfsd2b	0.694285168	0.000282333
mPEPb	ENSMUSG00000023031	Cela1	0.694165711	0.000281965
mPEPb	ENSMUSG00000017733	Eppin	0.693783448	0.000245649
mPEPb	ENSMUSG00000046378	Asphd1	0.693018922	0.000356119
mPEPb	ENSMUSG00000040133	Gpr176	0.692947248	0.000359039
mPEPb	ENSMUSG00000106320	NA	0.69215883	0.000383927
mPEPb	ENSMUSG00000052135	Foxo6	0.691083716	0.00046228

mPEPb	ENSMUSG00000063506	Arhgap22	0.691083716	0.000481374
mPEPb	ENSMUSG00000057897	Camk2b	0.691083716	0.000481584
mPEPb	ENSMUSG00000030089	Slc41a3	0.691035933	0.000484945
mPEPb	ENSMUSG00000021311	Mtr	0.69098815	0.000487889
mPEPb	ENSMUSG00000036526	Card11	0.690605887	0.00048039
mPEPb	ENSMUSG00000084866	A930006K02Rik	0.690366972	0.000526517
mPEPb	ENSMUSG00000029119	Man2b2	0.689650229	0.000599915
mPEPb	ENSMUSG00000031398	Plxna3	0.689626338	0.00060096
mPEPb	ENSMUSG00000103807	NA	0.689626338	0.000543383
mPEPb	ENSMUSG00000025026	Add3	0.68867068	0.00069614
mPEPb	ENSMUSG00000045008	9030612E09Rik	0.688575115	0.000651395
mPEPb	ENSMUSG00000027907	S100a11	0.688025612	0.000769434
mPEPb	ENSMUSG00000044337	Ackr3	0.687906154	0.000749754
mPEPb	ENSMUSG00000023067	Cdkn1a	0.68783448	0.000792619
mPEPb	ENSMUSG00000043556	Fbxl7	0.68766724	0.000760037
mPEPb	ENSMUSG00000068082	Grxcr1	0.687547783	0.000729622
mPEPb	ENSMUSG00000020326	Ccng1	0.687547783	0.000827724
mPEPb	ENSMUSG00000085620	NA	0.687117737	0.000670234
mPEPb	ENSMUSG00000046841	Ckap4	0.686926606	0.000907803
mPEPb	ENSMUSG00000038459	Abhd17c	0.686568234	0.000959244
mPEPb	ENSMUSG00000027408	Cpxm1	0.686544343	0.000945125
mPEPb	ENSMUSG00000021070	Bdkrb2	0.686018731	0.000969425
mPEPb	ENSMUSG00000024338	Psmb8	0.685779817	0.001079984
mPEPb	ENSMUSG00000036777	Anln	0.685660359	0.001066011
mPEPb	ENSMUSG00000078238	NA	0.685325879	0.001103905
mPEPb	ENSMUSG00000030255	Sspn	0.685301988	0.001146999
mPEPb	ENSMUSG00000037138	Aff3	0.685206422	0.001177788
mPEPb	ENSMUSG00000011267	Zfp296	0.684728593	0.001201097
mPEPb	ENSMUSG00000041141	Pnmal1	0.684418005	0.00132276
mPEPb	ENSMUSG00000087366	Junos	0.683629587	0.001436126
mPEPb	ENSMUSG00000025742	Prps2	0.683438456	0.001532698
mPEPb	ENSMUSG00000040848	Sft2d2	0.682793387	0.001679001
mPEPb	ENSMUSG00000042184	1700069L16Rik	0.682267775	0.001317467
mPEPb	ENSMUSG00000097440	NA	0.681837729	0.001786242
mPEPb	ENSMUSG00000032059	Alg9	0.681574924	0.0020151
mPEPb	ENSMUSG00000100975	NA	0.680404243	0.002385228
mPEPb	ENSMUSG00000020660	Pomc	0.679711391	0.002576414
mPEPb	ENSMUSG00000021180	Rps6ka5	0.679424694	0.002758734
mPEPb	ENSMUSG00000005045	Chd5	0.679281346	0.002822371
mPEPb	ENSMUSG00000031380	Figf	0.678445145	0.003039023
mPEPb	ENSMUSG00000040490	Lrfn2	0.678397362	0.003078448
mPEPb	ENSMUSG00000041341	Atg2b	0.678373471	0.003215819
mPEPb	ENSMUSG00000021540	Smad5	0.678254014	0.003268116

mPEPb	ENSMUSG00000070047	Fat1	0.678158448	0.003124053
mPEPb	ENSMUSG00000026185	Igfbp5	0.678038991	0.002996686
mPEPb	ENSMUSG00000022454	Nell2	0.678015099	0.003161345
mPEPb	ENSMUSG00000000416	Cttnbp2	0.677346139	0.003712486
mPEPb	ENSMUSG00000073295	Nudt11	0.677083333	0.003870173
mPEPb	ENSMUSG00000021575	Ahrr	0.676844419	0.003846562
mPEPb	ENSMUSG00000072964	Bhlhb9	0.676844419	0.004004334
mPEPb	ENSMUSG00000056553	Ptprn2	0.676629396	0.004129358
mPEPb	ENSMUSG00000027230	Creb3l1	0.67603211	0.004495115
mPEPb	ENSMUSG00000078496	Gm13152	0.675578173	0.004787567
mPEPb	ENSMUSG00000020423	Btg2	0.67486143	0.005311598
mPEPb	ENSMUSG00000053552	Ebf4	0.674765864	0.005363361
mPEPb	ENSMUSG00000039154	Shd	0.674598624	0.005510392
mPEPb	ENSMUSG00000104168	NA	0.674096904	0.005822612
mPEPb	ENSMUSG00000034987	Hrh2	0.673857989	0.005324227
mPEPb	ENSMUSG00000053799	Exoc6	0.673762424	0.006207224
mPEPb	ENSMUSG00000057346	Apol9a	0.672782875	0.006385733
mPEPb	ENSMUSG00000039137	Whrn	0.6727112	0.006440478
mPEPb	ENSMUSG00000074922	Fam122a	0.672424503	0.00749269
mPEPb	ENSMUSG00000005125	Ndrg1	0.671922783	0.007983445
mPEPb	ENSMUSG00000044847	Lsm11	0.671492737	0.008502687
mPEPb	ENSMUSG00000030519	Apba2	0.671229931	0.008842128
mPEPb	ENSMUSG00000028689	Ccdc163	0.67120604	0.008650571
mPEPb	ENSMUSG00000024818	Slc25a45	0.670489297	0.009533309
mPEPb	ENSMUSG00000024219	Anks1	0.670345948	0.009992605
mPEPb	ENSMUSG00000026247	Ecel1	0.670107034	0.007944941
mPEPb	ENSMUSG00000020427	Igfbp3	0.670011468	0.010310826
mPEPb	ENSMUSG00000067377	Tspan6	0.669892011	0.010625527
mPEPb	ENSMUSG00000099893	NA	0.669342508	0.008716815
mPEPb	ENSMUSG00000099444	NA	0.669127485	0.010613097
mPEPb	ENSMUSG00000071392	Ect2l	0.668912462	0.011144517
mPEPb	ENSMUSG00000021916	Glt8d1	0.668769113	0.012399699
mPEPb	ENSMUSG00000039059	Hrh3	0.668577982	0.012360639
mPEPb	ENSMUSG00000017204	Gsdma	0.668506307	0.011127404
mPEPb	ENSMUSG00000050623	Tex40	0.668362959	0.013079935
mPEPb	ENSMUSG00000024754	Tmem2	0.668315176	0.013184266
mPEPb	ENSMUSG00000022766	Serpind1	0.667574541	0.012967676
mPEPb	ENSMUSG00000031028	Tub	0.667120604	0.015464995
mPEPb	ENSMUSG00000021686	Ap3b1	0.66704893	0.015654864
mPEPb	ENSMUSG00000063628	NA	0.666308295	0.01720723
mPEPb	ENSMUSG00000032867	Fbxw8	0.665997706	0.01802151
mPEPb	ENSMUSG00000093271	NA	0.665878249	0.015418896
mPEPb	ENSMUSG00000074671	Tspyl3	0.665663226	0.018840271

mPEPb	ENSMUSG00000074170	Plekhf1	0.665472095	0.01748256
mPEPb	ENSMUSG00000057060	Slc35f3	0.664827026	0.020904785
mPEPb	ENSMUSG00000022439	Parvg	0.664134174	0.020420866
mPEPb	ENSMUSG00000045777	Ifitm10	0.664134174	0.023108691
mPEPb	ENSMUSG00000087296	NA	0.664086391	0.017321522
mPEPb	ENSMUSG00000025870	Arl10	0.663297974	0.025844578
mPEPb	ENSMUSG00000028108	Ecm1	0.663154625	0.026135243
mPEPb	ENSMUSG00000087382	Ctcflos	0.662533448	0.026588072
mPEPb	ENSMUSG00000021973	Micu2	0.662318425	0.029406103
mPEPb	ENSMUSG00000004864	Mapk13	0.661721139	0.03010699
mPEPb	ENSMUSG00000054582	Pabpc1l	0.661625573	0.031871649
mPEPb	ENSMUSG00000027611	Procr	0.661267202	0.029710006
mPEPb	ENSMUSG00000074378	Bsph1	0.660837156	0.027478343
mPEPb	ENSMUSG00000067973	Gm6994	0.660717699	0.034612274
mPEPb	ENSMUSG00000097749	NA	0.660693807	0.035530261
mPEPb	ENSMUSG00000019027	Dnah1	0.660550459	0.033867872
mPEPb	ENSMUSG00000099162	NA	0.659379778	0.036040747
mPEPb	ENSMUSG00000055320	Tead1	0.659355887	0.043123891
mPEPb	ENSMUSG00000041773	Enc1	0.658448012	0.048251676
pPEP	ENSMUSG00000059974	Ntm	0.952897809	5.73E-23
pPEP	ENSMUSG00000019929	Dcn	0.930165882	2.06E-20
pPEP	ENSMUSG00000000214	Th	0.90347464	1.26E-17
pPEP	ENSMUSG00000003476	Crhr2	0.895453615	6.65E-17
pPEP	ENSMUSG00000005973	Rcn1	0.882346918	1.36E-15
pPEP	ENSMUSG00000036437	Npy1r	0.875213325	6.13E-15
pPEP	ENSMUSG00000063260	Syt10	0.855485016	3.13E-13
pPEP	ENSMUSG00000029999	Tgfa	0.85200355	6.97E-13
pPEP	ENSMUSG00000037014	Sstr4	0.850706533	8.89E-13
pPEP	ENSMUSG00000097248	Gm2694	0.848317291	1.47E-12
pPEP	ENSMUSG00000037386	Rims2	0.842617243	4.63E-12
pPEP	ENSMUSG00000029503	P2rx2	0.840432794	5.86E-12
pPEP	ENSMUSG00000028464	Tpm2	0.839238173	7.77E-12
pPEP	ENSMUSG00000026748	Plxdc2	0.833640522	2.55E-11
pPEP	ENSMUSG00000005952	Trpv1	0.832206977	3.36E-11
pPEP	ENSMUSG00000038059	Smim3	0.831660864	3.73E-11
pPEP	ENSMUSG00000028785	Hpca	0.829339887	5.69E-11
pPEP	ENSMUSG00000001510	Dlx3	0.827428493	7.54E-11
pPEP	ENSMUSG00000099521	NA	0.826575193	7.72E-11
pPEP	ENSMUSG00000036256	Igfbp7	0.82247935	1.77E-10
pPEP	ENSMUSG00000042499	Hoxd11	0.822103898	1.86E-10
pPEP	ENSMUSG00000064293	Cntn4	0.814594853	7.29E-10
pPEP	ENSMUSG00000030138	Bms1	0.814321797	8.86E-10
pPEP	ENSMUSG00000045092	S1pr1	0.8125128	1.19E-09

pPEP	ENSMUSG00000009292	Trpm2	0.811010991	1.59E-09
pPEP	ENSMUSG00000036699	Zcchc12	0.808928937	2.26E-09
pPEP	ENSMUSG00000024084	Qpct	0.806744488	3.30E-09
pPEP	ENSMUSG00000049265	Kcnk3	0.803331285	6.01E-09
pPEP	ENSMUSG00000066361	Serpina3c	0.801249232	7.91E-09
pPEP	ENSMUSG00000016520	Lnx2	0.800498328	9.65E-09
pPEP	ENSMUSG00000019817	Plagl1	0.800157007	1.04E-08
pPEP	ENSMUSG00000028786	Tmem54	0.799235443	1.14E-08
pPEP	ENSMUSG00000041261	Car8	0.797426445	1.52E-08
pPEP	ENSMUSG00000024420	Zfp521	0.792955151	3.29E-08
pPEP	ENSMUSG00000023966	Rsph9	0.78636767	1.02E-07
pPEP	ENSMUSG00000031654	Cbln1	0.784797597	1.02E-07
pPEP	ENSMUSG00000078721	Fam205a1	0.777834664	3.59E-07
pPEP	ENSMUSG00000025533	Asl	0.777732268	4.10E-07
pPEP	ENSMUSG00000050711	Scg2	0.776230459	5.19E-07
pPEP	ENSMUSG00000021187	Tc2n	0.774182538	6.59E-07
pPEP	ENSMUSG00000048978	Nrsn1	0.773158577	8.36E-07
pPEP	ENSMUSG00000041594	Tmtc4	0.772817257	8.81E-07
pPEP	ENSMUSG00000030677	Kif22	0.76831183	1.76E-06
pPEP	ENSMUSG00000066705	Fxyd6	0.766024985	2.47E-06
pPEP	ENSMUSG00000017057	Il13ra1	0.763908799	3.36E-06
pPEP	ENSMUSG00000074825	Itpripl1	0.762714178	4.07E-06
pPEP	ENSMUSG00000046719	Nxph3	0.76155369	3.93E-06
pPEP	ENSMUSG00000038112	AW551984	0.761417162	3.71E-06
pPEP	ENSMUSG00000059674	Cdh24	0.758891392	5.90E-06
pPEP	ENSMUSG00000089706	B230216N24Rik	0.757799167	8.37E-06
pPEP	ENSMUSG00000021108	Prkch	0.756433886	9.76E-06
pPEP	ENSMUSG00000061762	Tac1	0.756365622	1.04E-05
pPEP	ENSMUSG00000036298	Slc2a13	0.753191344	1.60E-05
pPEP	ENSMUSG00000055026	Gabrg3	0.750221858	2.38E-05
pPEP	ENSMUSG00000035258	Abi3bp	0.746501468	3.68E-05
pPEP	ENSMUSG00000044461	Shisa2	0.746194279	3.90E-05
pPEP	ENSMUSG00000087142	NA	0.743190661	6.05E-05
pPEP	ENSMUSG00000053194	Cib4	0.741893645	6.24E-05
pPEP	ENSMUSG00000023965	Fbxl17	0.741415796	8.52E-05
pPEP	ENSMUSG00000062257	Opcml	0.739572667	0.000109545
pPEP	ENSMUSG00000022863	Btg3	0.738753499	0.00011173
pPEP	ENSMUSG00000030223	Ptpro	0.737661274	0.000141176
pPEP	ENSMUSG00000032322	Pstpip1	0.737354086	0.000147125
pPEP	ENSMUSG00000031343	Gabra3	0.735784013	0.000176018
pPEP	ENSMUSG00000032607	Amt	0.732575602	0.000277859
pPEP	ENSMUSG00000046523	Kctd4	0.731585774	0.000290013
pPEP	ENSMUSG00000034783	Cd207	0.731244454	0.000220217

pPEP	ENSMUSG00000005949	Ctns	0.73117619	0.0003353
pPEP	ENSMUSG00000085067	NA	0.730220493	0.000281982
pPEP	ENSMUSG00000005951	Shpk	0.729128268	0.000436726
pPEP	ENSMUSG00000031066	Usp11	0.728718684	0.000462098
pPEP	ENSMUSG00000039997	Ifi203	0.723701277	0.000820179
pPEP	ENSMUSG00000024254	Abcg8	0.722813844	0.000855379
pPEP	ENSMUSG00000073968	Trim68	0.722438392	0.001027136
pPEP	ENSMUSG00000042289	Hsd3b7	0.722028807	0.001081284
pPEP	ENSMUSG00000022180	Slc7a8	0.72056113	0.001302886
pPEP	ENSMUSG00000070803	Cited4	0.720015018	0.001345404
pPEP	ENSMUSG00000021871	Pnp	0.719741962	0.001443992
pPEP	ENSMUSG00000083750	NA	0.718854529	0.001253949
pPEP	ENSMUSG00000027977	Ndst3	0.717932965	0.001802669
pPEP	ENSMUSG00000006931	P3h4	0.71632876	0.00218656
pPEP	ENSMUSG00000074115	Saa1	0.716226364	0.002008258
pPEP	ENSMUSG00000038026	Kcnj9	0.715577855	0.001845156
pPEP	ENSMUSG00000034853	Acot11	0.713393406	0.00309944
pPEP	ENSMUSG00000040978	Gm11992	0.713017953	0.003243303
pPEP	ENSMUSG00000000686	Abhd15	0.712608369	0.003320858
pPEP	ENSMUSG00000022021	Diaph3	0.711345484	0.004018731
pPEP	ENSMUSG00000048540	Nhlh2	0.710014335	0.004609472
pPEP	ENSMUSG00000026437	Cdk18	0.709980203	0.004495115
pPEP	ENSMUSG00000014599	Csf1	0.709536487	0.004974275
pPEP	ENSMUSG00000031024	St5	0.708375998	0.005676548
pPEP	ENSMUSG00000057465	Saa2	0.708341866	0.004942888
pPEP	ENSMUSG00000029878	Dbpht2	0.706805925	0.006895088
pPEP	ENSMUSG00000000126	Wnt9a	0.705747833	0.007748862
pPEP	ENSMUSG00000046093	Hpcal4	0.704758004	0.008642335
pPEP	ENSMUSG00000052397	Ezr	0.704484948	0.009034419
pPEP	ENSMUSG00000083498	NA	0.70417776	0.007609334
pPEP	ENSMUSG00000019850	Tnfaip3	0.703904703	0.009375809
pPEP	ENSMUSG00000039385	Cdh6	0.703904703	0.009540234
pPEP	ENSMUSG00000039462	Col10a1	0.703324459	0.008639948
pPEP	ENSMUSG00000042401	Crtac1	0.702641819	0.011166758
pPEP	ENSMUSG00000005672	Kit	0.701754386	0.0123462
pPEP	ENSMUSG00000085992	NA	0.701754386	0.010049489
pPEP	ENSMUSG00000104860	NA	0.701003481	0.012441122
pPEP	ENSMUSG00000036139	Hoxc9	0.700525633	0.01289032
pPEP	ENSMUSG00000026922	Agpat2	0.700354973	0.014088911
pPEP	ENSMUSG00000020654	Adcy3	0.700320841	0.014534786
pPEP	ENSMUSG00000039579	Grin3a	0.699911257	0.01521658
pPEP	ENSMUSG00000097074	4833428L15Rik	0.69827292	0.015153743
pPEP	ENSMUSG00000070366	Plpp4	0.697897467	0.018323216

pPEP	ENSMUSG00000049892	Rasd1	0.696839375	0.021449763
pPEP	ENSMUSG00000063430	Wscd2	0.696566319	0.021513674
pPEP	ENSMUSG00000102101	Zbtb11os1	0.696463922	0.02232415
pPEP	ENSMUSG00000031210	Gpr165	0.695713018	0.024156602
pPEP	ENSMUSG00000018451	NA	0.695132774	0.026008191
pPEP	ENSMUSG00000034435	Tmem30b	0.694484265	0.025482875
pPEP	ENSMUSG00000061535	C1qtnf7	0.694381869	0.028164484
pPEP	ENSMUSG00000024867	Pip5k1b	0.693938153	0.029550534
pPEP	ENSMUSG00000057329	Bcl2	0.69236808	0.035147871
pPEP	ENSMUSG00000034758	Tle6	0.69219742	0.035499812
pPEP	ENSMUSG00000024256	Adcyap1	0.691924363	0.0368851
pPEP	ENSMUSG00000031681	Smad1	0.691378251	0.039167337
pPEP	ENSMUSG00000020241	Col6a2	0.691241723	0.039112321
pPEP	ENSMUSG00000048721	Fndc9	0.689739914	0.045972966
pPEP	ENSMUSG00000009291	Pttg1ip	0.68933033	0.048908876
pPEP	ENSMUSG00000030866	Ern2	0.689296198	0.040310822
pNF	ENSMUSG00000022123	Scel	0.98280303	1.60E-23
pNF	ENSMUSG00000025739	Gng13	0.97719697	2.30E-23
pNF	ENSMUSG00000035273	Hpse	0.975416667	3.94E-23
pNF	ENSMUSG00000031355	Arhgap6	0.975075758	1.10E-22
pNF	ENSMUSG00000054477	Kcnn2	0.972727273	2.43E-22
pNF	ENSMUSG00000022762	Ncam2	0.971969697	2.85E-22
pNF	ENSMUSG00000056423	Uts2b	0.971856061	7.86E-23
pNF	ENSMUSG00000026110	Mgat4a	0.968484848	6.72E-22
pNF	ENSMUSG00000039488	Cntn5	0.968257576	5.39E-22
pNF	ENSMUSG00000063446	Plppr1	0.965681818	6.22E-22
pNF	ENSMUSG00000042589	Cux2	0.965530303	1.14E-21
pNF	ENSMUSG00000036095	Dgkb	0.964242424	1.28E-21
pNF	ENSMUSG00000040254	Sema3d	0.963939394	1.16E-21
pNF	ENSMUSG00000036854	Hspb6	0.961818182	3.11E-21
pNF	ENSMUSG00000047712	Ust	0.957651515	7.19E-21
pNF	ENSMUSG00000024526	Cidea	0.954469697	1.42E-20
pNF	ENSMUSG00000040147	Maob	0.954166667	1.39E-20
pNF	ENSMUSG00000029851	Tcaf2	0.948106061	6.07E-20
pNF	ENSMUSG00000034109	Golim4	0.948030303	7.74E-20
pNF	ENSMUSG00000036103	Colec12	0.946969697	3.21E-20
pNF	ENSMUSG00000100706	NA	0.940530303	1.69E-19
pNF	ENSMUSG00000000093	Tbx2	0.939924242	4.56E-19
pNF	ENSMUSG00000041741	Pde3a	0.939583333	2.56E-19
pNF	ENSMUSG00000061132	Blnk	0.937537879	2.88E-19
pNF	ENSMUSG00000031561	Tenm3	0.936893939	8.13E-19
pNF	ENSMUSG00000059901	Adamts14	0.936590909	4.97E-19
pNF	ENSMUSG00000031298	Adgrg2	0.935151515	1.34E-18

pNF	ENSMUSG00000020053	Igf1	0.934507576	5.46E-19
pNF	ENSMUSG00000049493	Pls1	0.934242424	1.16E-18
pNF	ENSMUSG00000019768	Esr1	0.932878788	2.16E-18
pNF	ENSMUSG00000048281	Dleu7	0.929924242	2.23E-18
pNF	ENSMUSG00000030790	Adm	0.926666667	7.75E-18
pNF	ENSMUSG00000024866	Acy3	0.925681818	8.53E-18
pNF	ENSMUSG00000045589	Frrs1l	0.925681818	9.37E-18
pNF	ENSMUSG00000063296	Tmem117	0.92530303	1.20E-17
pNF	ENSMUSG00000031245	Hmgn5	0.924848485	1.12E-17
pNF	ENSMUSG00000020015	Cdk17	0.924242424	1.41E-17
pNF	ENSMUSG00000053647	Gper1	0.922424242	9.26E-18
pNF	ENSMUSG00000027966	Col11a1	0.921818182	1.90E-17
pNF	ENSMUSG00000046997	Spsb4	0.918636364	4.97E-17
pNF	ENSMUSG00000002059	Rab34	0.917007576	5.67E-17
pNF	ENSMUSG00000034755	Pcdh11x	0.915719697	4.47E-17
pNF	ENSMUSG00000009654	Oit3	0.915568182	4.09E-17
pNF	ENSMUSG00000061758	Akr1b10	0.915227273	8.31E-17
pNF	ENSMUSG00000035473	Galm	0.915227273	8.11E-17
pNF	ENSMUSG00000052026	Slc6a7	0.914166667	7.59E-17
pNF	ENSMUSG00000052911	Lamb2	0.913901515	8.96E-17
pNF	ENSMUSG00000019945	1700040L02Rik	0.913863636	5.16E-17
pNF	ENSMUSG00000074971	Fibin	0.912992424	6.03E-17
pNF	ENSMUSG00000040037	Negr1	0.9125	1.80E-16
pNF	ENSMUSG00000006221	Hspb7	0.911287879	7.93E-17
pNF	ENSMUSG00000003420	Fcgrt	0.909924242	2.55E-16
pNF	ENSMUSG00000073574	Grxcr2	0.908409091	2.47E-16
pNF	ENSMUSG00000029101	Rgs12	0.90780303	4.14E-16
pNF	ENSMUSG00000023972	Ptk7	0.906136364	6.07E-16
pNF	ENSMUSG00000055322	Tns1	0.904469697	8.62E-16
pNF	ENSMUSG00000032269	Htr3a	0.904015152	1.05E-15
pNF	ENSMUSG00000082674	NA	0.903560606	5.69E-16
pNF	ENSMUSG00000021846	Peli2	0.902272727	1.50E-15
pNF	ENSMUSG00000005803	Sqrdl	0.902045455	1.19E-15
pNF	ENSMUSG00000033022	Cdo1	0.901666667	1.64E-15
pNF	ENSMUSG00000033342	Plppr5	0.899545455	1.88E-15
pNF	ENSMUSG00000029771	Irf5	0.899507576	2.13E-15
pNF	ENSMUSG00000032714	Syde1	0.899090909	1.67E-15
pNF	ENSMUSG00000029304	Spp1	0.898901515	1.99E-15
pNF	ENSMUSG00000020085	Aifm2	0.898636364	2.45E-15
pNF	ENSMUSG00000034472	Rasd2	0.898106061	2.48E-15
pNF	ENSMUSG00000021587	Pcsk1	0.897878788	3.71E-15
pNF	ENSMUSG00000030341	Tnfrsf1a	0.89719697	3.26E-15
pNF	ENSMUSG00000001656	Hoxc11	0.896212121	3.09E-15

pNF	ENSMUSG00000024805	Pcgf5	0.895757576	4.97E-15
pNF	ENSMUSG00000022425	Enpp2	0.895568182	4.30E-15
pNF	ENSMUSG00000039481	Nrtn	0.89530303	3.04E-15
pNF	ENSMUSG00000044674	Fzd1	0.894507576	5.73E-15
pNF	ENSMUSG00000018862	Otop3	0.893484848	7.09E-15
pNF	ENSMUSG00000053025	Sv2b	0.893181818	9.45E-15
pNF	ENSMUSG00000033350	Chst2	0.89280303	1.03E-14
pNF	ENSMUSG00000020848	Doc2b	0.892007576	7.48E-15
pNF	ENSMUSG00000048376	F2r	0.891212121	1.09E-14
pNF	ENSMUSG00000075297	H60b	0.891174242	6.67E-15
pNF	ENSMUSG00000046417	Lrrc75a	0.890568182	9.28E-15
pNF	ENSMUSG00000091811	Inafm1	0.89030303	1.69E-14
pNF	ENSMUSG00000034135	Sik3	0.889924242	1.79E-14
pNF	ENSMUSG00000032609	Klhdc8b	0.888257576	2.53E-14
pNF	ENSMUSG00000056215	Lrguk	0.888106061	2.57E-14
pNF	ENSMUSG00000025402	Nab2	0.887045455	2.70E-14
pNF	ENSMUSG00000019487	Trip10	0.885416667	4.09E-14
pNF	ENSMUSG00000032360	Hcrtr2	0.885113636	2.85E-14
pNF	ENSMUSG00000051022	Hs3st1	0.884545455	4.27E-14
pNF	ENSMUSG00000029086	Prom1	0.883257576	3.43E-14
pNF	ENSMUSG00000098158	NA	0.883257576	5.86E-14
pNF	ENSMUSG00000050368	Hoxd10	0.882121212	6.97E-14
pNF	ENSMUSG00000027078	Ube2l6	0.881893939	8.82E-14
pNF	ENSMUSG00000027263	Tubgcp4	0.881363636	9.77E-14
pNF	ENSMUSG00000094500	Smim18	0.881287879	9.47E-14
pNF	ENSMUSG00000022358	Fbxo32	0.881212121	9.96E-14
pNF	ENSMUSG00000079657	Rab26	0.880416667	1.07E-13
pNF	ENSMUSG00000027560	Dok5	0.88030303	1.14E-13
pNF	ENSMUSG00000097618	NA	0.88030303	5.61E-14
pNF	ENSMUSG00000050619	Zscan29	0.879848485	1.31E-13
pNF	ENSMUSG00000030339	Ltbr	0.879848485	1.25E-13
pNF	ENSMUSG00000048497	Mmgt2	0.878939394	1.51E-13
pNF	ENSMUSG00000059361	Nrsn2	0.876893939	2.30E-13
pNF	ENSMUSG00000074218	Cox7a1	0.876818182	2.32E-13
pNF	ENSMUSG00000017724	Etv4	0.876704545	1.13E-13
pNF	ENSMUSG00000036782	Klhl13	0.875909091	2.79E-13
pNF	ENSMUSG00000022525	Hrasls	0.875757576	1.58E-13
pNF	ENSMUSG00000034295	Fhod3	0.87344697	4.08E-13
pNF	ENSMUSG00000039628	Hs3st6	0.873181818	2.56E-13
pNF	ENSMUSG00000049097	Ankrd34a	0.872878788	4.66E-13
pNF	ENSMUSG00000000792	Slc5a5	0.871212121	6.48E-13
pNF	ENSMUSG00000032348	Gsta4	0.871136364	6.77E-13
pNF	ENSMUSG00000021710	Nln	0.870909091	7.17E-13

pNF	ENSMUSG00000055540	Epha6	0.870757576	4.33E-13
pNF	ENSMUSG00000061171	Slc38a11	0.870606061	3.00E-13
pNF	ENSMUSG00000034488	Edil3	0.869545455	9.34E-13
pNF	ENSMUSG00000036907	C1ql2	0.869242424	7.03E-13
pNF	ENSMUSG00000020806	Rhbdf2	0.868787879	7.15E-13
pNF	ENSMUSG00000035109	Shc4	0.868636364	9.78E-13
pNF	ENSMUSG00000035835	Plppr3	0.868560606	1.05E-12
pNF	ENSMUSG00000032246	Calml4	0.868560606	1.06E-12
pNF	ENSMUSG00000044258	Ctla2a	0.868181818	4.83E-13
pNF	ENSMUSG00000020473	Aebp1	0.867537879	8.17E-13
pNF	ENSMUSG00000026610	Esrrg	0.864280303	1.42E-12
pNF	ENSMUSG00000034613	Ppm1h	0.863106061	3.10E-12
pNF	ENSMUSG00000030731	Syt3	0.862348485	3.33E-12
pNF	ENSMUSG00000045094	Arhgef37	0.861098485	3.30E-12
pNF	ENSMUSG00000031870	Pgr	0.859810606	5.42E-12
pNF	ENSMUSG00000027297	Ltk	0.859204545	3.01E-12
pNF	ENSMUSG00000008590	Htr3b	0.85905303	6.02E-12
pNF	ENSMUSG00000021760	Gpx8	0.85844697	6.77E-12
pNF	ENSMUSG00000017491	Rarb	0.857840909	6.35E-12
pNF	ENSMUSG00000096936	NA	0.856969697	6.71E-12
pNF	ENSMUSG00000103255	Pcdhac1	0.856666667	9.95E-12
pNF	ENSMUSG00000040373	Cacng5	0.856212121	1.01E-11
pNF	ENSMUSG00000053158	Fes	0.855984848	5.69E-12
pNF	ENSMUSG00000021895	Arhgef3	0.854810606	1.28E-11
pNF	ENSMUSG00000050822	Slc29a4	0.854242424	1.57E-11
pNF	ENSMUSG00000052364	B630019K06Rik	0.853560606	1.77E-11
pNF	ENSMUSG00000020178	Adora2a	0.853522727	1.69E-11
pNF	ENSMUSG00000032387	Rbpms2	0.853219697	1.06E-11
pNF	ENSMUSG00000038515	Grtp1	0.850909091	2.08E-11
pNF	ENSMUSG00000040520	Manea	0.850643939	2.98E-11
pNF	ENSMUSG00000026167	Wnt10a	0.850037879	1.76E-11
pNF	ENSMUSG00000047658	Gal3st3	0.84905303	2.77E-11
pNF	ENSMUSG00000044067	Gpr22	0.847878788	4.86E-11
pNF	ENSMUSG00000002459	Rgs20	0.847727273	4.51E-11
pNF	ENSMUSG00000044716	Dok7	0.846628788	5.72E-11
pNF	ENSMUSG00000034796	Cpne7	0.846136364	2.62E-11
pNF	ENSMUSG00000031409	Tceal6	0.845568182	6.70E-11
pNF	ENSMUSG00000000690	Hoxb6	0.845113636	6.40E-11
pNF	ENSMUSG00000023015	Racgap1	0.844318182	8.04E-11
pNF	ENSMUSG00000024376	Epb41l4a	0.842916667	9.52E-11
pNF	ENSMUSG00000060591	Ifitm2	0.841515152	1.42E-10
pNF	ENSMUSG00000026173	Plcd4	0.840454545	1.78E-10
pNF	ENSMUSG00000021226	Acot2	0.839772727	2.01E-10

pNF	ENSMUSG00000020656	Grhl1	0.839734848	1.45E-10
pNF	ENSMUSG00000000440	Pparg	0.839583333	1.64E-10
pNF	ENSMUSG00000049612	Omg	0.837992424	2.26E-10
pNF	ENSMUSG00000032625	Thsd7a	0.836893939	3.32E-10
pNF	ENSMUSG00000032417	Rwdd2a	0.836666667	3.39E-10
pNF	ENSMUSG00000086596	Susd5	0.835871212	2.37E-10
pNF	ENSMUSG00000059277	R74862	0.834734848	2.80E-10
pNF	ENSMUSG00000056306	Sertm1	0.833977273	5.28E-10
pNF	ENSMUSG00000032816	Igdcc4	0.833598485	4.91E-10
pNF	ENSMUSG00000058571	Gpc6	0.832878788	6.50E-10
pNF	ENSMUSG00000078350	Smim1	0.832348485	6.92E-10
pNF	ENSMUSG00000014813	Stc1	0.83219697	3.20E-10
pNF	ENSMUSG00000020773	Trim47	0.831704545	5.94E-10
pNF	ENSMUSG00000024451	Arap3	0.831628788	6.55E-10
pNF	ENSMUSG00000024349	Tmem173	0.831515152	6.86E-10
pNF	ENSMUSG00000041078	Grid1	0.831136364	7.69E-10
pNF	ENSMUSG00000074682	Zcchc3	0.830909091	9.13E-10
pNF	ENSMUSG00000030606	Hapln3	0.830265152	7.42E-10
pNF	ENSMUSG00000000938	Hoxa10	0.829166667	1.21E-09
pNF	ENSMUSG00000050132	Sarm1	0.828333333	1.42E-09
pNF	ENSMUSG00000043857	Mgat5b	0.828257576	1.38E-09
pNF	ENSMUSG00000048240	Gng7	0.827083333	1.65E-09
pNF	ENSMUSG00000042155	Klhl23	0.826704545	1.41E-09
pNF	ENSMUSG00000050103	Agmo	0.826287879	1.23E-09
pNF	ENSMUSG00000049176	Frmpd4	0.825681818	2.11E-09
pNF	ENSMUSG00000035342	Lzts2	0.825416667	2.17E-09
pNF	ENSMUSG00000003469	Phyhip	0.824166667	2.70E-09
pNF	ENSMUSG00000035105	Egln3	0.82405303	1.49E-09
pNF	ENSMUSG00000091475	2810468N07Rik	0.823977273	1.91E-09
pNF	ENSMUSG00000071856	Mcc	0.823977273	2.88E-09
pNF	ENSMUSG00000036144	Meox2	0.823787879	1.33E-09
pNF	ENSMUSG00000067279	Ppp1r3c	0.822348485	2.66E-09
pNF	ENSMUSG00000005057	Sh2b2	0.822045455	3.74E-09
pNF	ENSMUSG00000030605	Mfge8	0.821931818	3.61E-09
pNF	ENSMUSG00000049555	Tmie	0.821022727	2.54E-09
pNF	ENSMUSG00000022799	Arhgap31	0.820454545	5.14E-09
pNF	ENSMUSG00000032495	Lrrc2	0.819583333	5.60E-09
pNF	ENSMUSG00000030518	Fam189a1	0.81844697	6.46E-09
pNF	ENSMUSG00000028378	Ptgr1	0.817954545	7.77E-09
pNF	ENSMUSG00000040703	Cyp2s1	0.817310606	5.93E-09
pNF	ENSMUSG00000020598	Nrcam	0.815454545	1.17E-08
pNF	ENSMUSG00000053963	6330403A02Rik	0.814128788	1.09E-08
pNF	ENSMUSG00000011305	Plin5	0.814015152	7.75E-09

pNF	ENSMUSG00000082361	Btc	0.813939394	1.34E-08
pNF	ENSMUSG00000071369	Map3k5	0.813787879	1.53E-08
pNF	ENSMUSG00000032303	Chrna3	0.813560606	1.56E-08
pNF	ENSMUSG00000021379	Id4	0.813484848	1.61E-08
pNF	ENSMUSG00000014932	Yes1	0.812878788	1.76E-08
pNF	ENSMUSG00000031548	Sfrp1	0.812651515	1.79E-08
pNF	ENSMUSG00000024427	Spry4	0.810909091	2.43E-08
pNF	ENSMUSG00000038319	Kcnh2	0.810530303	2.44E-08
pNF	ENSMUSG00000104696	NA	0.810189394	1.88E-08
pNF	ENSMUSG00000097745	AI115009	0.81	2.34E-08
pNF	ENSMUSG00000024601	Isoc1	0.809545455	3.02E-08
pNF	ENSMUSG00000027296	Itpka	0.808484848	1.94E-08
pNF	ENSMUSG00000002885	Adgre5	0.806590909	3.71E-08
pNF	ENSMUSG00000044968	Napepld	0.806515152	4.57E-08
pNF	ENSMUSG00000052373	Mpp3	0.805984848	5.23E-08
pNF	ENSMUSG00000028488	Sh3gl2	0.805454545	5.74E-08
pNF	ENSMUSG00000056899	Immp2l	0.804621212	6.48E-08
pNF	ENSMUSG00000029378	Areg	0.804318182	4.00E-08
pNF	ENSMUSG00000032431	Crtap	0.804318182	6.80E-08
pNF	ENSMUSG00000044646	Zbtb7c	0.803863636	6.11E-08
pNF	ENSMUSG00000030137	Tuba8	0.803787879	5.16E-08
pNF	ENSMUSG00000026471	Mr1	0.803636364	6.12E-08
pNF	ENSMUSG00000019822	Smpd2	0.803295455	7.46E-08
pNF	ENSMUSG00000038543	BC028528	0.803219697	7.65E-08
pNF	ENSMUSG00000035566	Pcdh17	0.803106061	8.26E-08
pNF	ENSMUSG00000020407	Upp1	0.801515152	1.02E-07
pNF	ENSMUSG00000024087	Cyp1b1	0.801477273	1.02E-07
pNF	ENSMUSG00000021974	Fgf9	0.800719697	1.09E-07
pNF	ENSMUSG00000020810	Cygb	0.799924242	1.35E-07
pNF	ENSMUSG00000021947	Cryl1	0.799772727	1.38E-07
pNF	ENSMUSG00000023249	Parp3	0.799166667	1.48E-07
pNF	ENSMUSG00000022044	Stmn4	0.798787879	1.56E-07
pNF	ENSMUSG00000086903	Hotair	0.797878788	1.38E-07
pNF	ENSMUSG00000041180	Hectd2	0.797575758	1.89E-07
pNF	ENSMUSG00000040653	Ppp1r14c	0.797310606	1.97E-07
pNF	ENSMUSG00000031246	Sh3bgrl	0.797045455	2.09E-07
pNF	ENSMUSG00000042594	Sh2b3	0.796704545	2.20E-07
pNF	ENSMUSG00000047344	Lancl3	0.796212121	2.30E-07
pNF	ENSMUSG00000038296	Galnt18	0.796060606	2.42E-07
pNF	ENSMUSG00000037548	H2-DMb2	0.795984848	1.82E-07
pNF	ENSMUSG00000022484	Hoxc10	0.795378788	2.38E-07
pNF	ENSMUSG00000001494	Sost	0.795265152	1.37E-07
pNF	ENSMUSG00000087201	NA	0.792840909	2.54E-07

pNF	ENSMUSG00000086555	Gm13446	0.79219697	2.95E-07
pNF	ENSMUSG00000054520	Sh3bp2	0.792083333	3.60E-07
pNF	ENSMUSG00000022817	Itgb5	0.791893939	3.98E-07
pNF	ENSMUSG00000006218	Fam131c	0.791515152	3.71E-07
pNF	ENSMUSG00000023092	Fhl1	0.791287879	4.97E-07
pNF	ENSMUSG00000002341	Ncan	0.790681818	5.03E-07
pNF	ENSMUSG00000027800	Tm4sf1	0.789393939	5.11E-07
pNF	ENSMUSG00000041736	Tspo	0.788939394	6.88E-07
pNF	ENSMUSG00000020589	Fam49a	0.788333333	7.66E-07
pNF	ENSMUSG00000055567	Unc80	0.787727273	8.39E-07
pNF	ENSMUSG00000011148	Adssl1	0.787348485	6.66E-07
pNF	ENSMUSG00000022376	Adcy8	0.787159091	8.05E-07
pNF	ENSMUSG00000020904	Cfap52	0.787083333	5.97E-07
pNF	ENSMUSG00000022441	Efcab6	0.785984848	9.55E-07
pNF	ENSMUSG00000044014	Npy5r	0.785795455	9.01E-07
pNF	ENSMUSG00000052889	Prkcb	0.785227273	1.17E-06
pNF	ENSMUSG00000037523	Mavs	0.784772727	1.22E-06
pNF	ENSMUSG00000051043	Gprc5c	0.784242424	1.34E-06
pNF	ENSMUSG00000041801	Phlda3	0.783712121	1.50E-06
pNF	ENSMUSG00000028434	Epb41l4b	0.783712121	1.42E-06
pNF	ENSMUSG00000033688	1300017J02Rik	0.783484848	1.42E-06
pNF	ENSMUSG00000003436	Dll3	0.783409091	1.48E-06
pNF	ENSMUSG00000058153	Sez6l	0.783371212	1.23E-06
pNF	ENSMUSG00000051067	Lingo3	0.783068182	1.35E-06
pNF	ENSMUSG00000025213	Kazald1	0.781931818	1.72E-06
pNF	ENSMUSG00000009646	Pla2g12b	0.781439394	1.24E-06
pNF	ENSMUSG00000078117	NA	0.78094697	1.89E-06
pNF	ENSMUSG00000048772	Tmem53	0.780643939	2.34E-06
pNF	ENSMUSG00000032392	Parp16	0.780530303	2.35E-06
pNF	ENSMUSG00000029864	Gstk1	0.780454545	2.28E-06
pNF	ENSMUSG00000019916	P4ha1	0.78030303	2.46E-06
pNF	ENSMUSG00000019055	Plod1	0.779545455	2.74E-06
pNF	ENSMUSG00000031022	BC051019	0.779507576	2.21E-06
pNF	ENSMUSG00000041920	Slc16a6	0.779204545	2.80E-06
pNF	ENSMUSG00000040713	Creg1	0.779015152	2.95E-06
pNF	ENSMUSG00000073102	Drc1	0.778409091	3.14E-06
pNF	ENSMUSG00000057729	Prtn3	0.77780303	2.39E-06
pNF	ENSMUSG00000029762	Akr1b8	0.777575758	3.23E-06
pNF	ENSMUSG00000021069	Pygl	0.777348485	3.74E-06
pNF	ENSMUSG00000059248	Sep-09	0.777272727	3.75E-06
pNF	ENSMUSG00000002897	Il17ra	0.776439394	4.09E-06
pNF	ENSMUSG00000034919	Ttc22	0.776363636	3.24E-06
pNF	ENSMUSG00000044134	Fam109a	0.775795455	4.64E-06

pNF	ENSMUSG00000039910	Cited2	0.775	5.22E-06
pNF	ENSMUSG00000003354	Ccdc65	0.774583333	5.14E-06
pNF	ENSMUSG00000000959	Oxa1l	0.774242424	5.81E-06
pNF	ENSMUSG00000037169	Mycn	0.774242424	5.67E-06
pNF	ENSMUSG00000039873	Neurl2	0.774204545	4.89E-06
pNF	ENSMUSG00000004655	Aqp1	0.774090909	5.92E-06
pNF	ENSMUSG00000029392	Rilpl1	0.773712121	6.24E-06
pNF	ENSMUSG00000046668	Cxxc5	0.772954545	6.93E-06
pNF	ENSMUSG00000056091	St3gal5	0.77219697	7.68E-06
pNF	ENSMUSG00000049858	Suox	0.772007576	7.83E-06
pNF	ENSMUSG00000017830	Dhx58	0.771856061	7.64E-06
pNF	ENSMUSG00000040972	Igsf21	0.771818182	7.20E-06
pNF	ENSMUSG00000036867	Smad6	0.771477273	6.50E-06
pNF	ENSMUSG00000047117	Ankdd1b	0.771325758	8.61E-06
pNF	ENSMUSG00000028527	Ak4	0.770681818	9.24E-06
pNF	ENSMUSG00000091586	Cyp4f17	0.770568182	5.87E-06
pNF	ENSMUSG00000038963	Slco4a1	0.769886364	8.95E-06
pNF	ENSMUSG00000020599	Rgs9	0.769166667	1.16E-05
pNF	ENSMUSG00000042364	Snx18	0.769015152	1.20E-05
pNF	ENSMUSG00000033063	Cntnap3	0.768901515	1.04E-05
pNF	ENSMUSG00000029126	Nsg1	0.768560606	1.27E-05
pNF	ENSMUSG00000053477	Tcf4	0.768409091	1.30E-05
pNF	ENSMUSG00000029370	Rassf6	0.767916667	1.30E-05
pNF	ENSMUSG00000052305	Hbb-b1	0.767083333	1.32E-05
pNF	ENSMUSG00000050534	Htr5b	0.767045455	1.56E-05
pNF	ENSMUSG00000037145	2210407C18Rik	0.766287879	1.38E-05
pNF	ENSMUSG00000034192	Lsm3	0.766136364	1.78E-05
pNF	ENSMUSG00000102215	Gm2464	0.765833333	1.84E-05
pNF	ENSMUSG00000030094	Xpc	0.765681818	1.88E-05
pNF	ENSMUSG00000020150	Gamt	0.765454545	1.95E-05
pNF	ENSMUSG00000105290	NA	0.764924242	1.52E-05
pNF	ENSMUSG00000074227	Spint2	0.763863636	2.38E-05
pNF	ENSMUSG00000022544	Eef2kmt	0.763863636	2.40E-05
pNF	ENSMUSG00000007837	Prrg2	0.76344697	2.55E-05
pNF	ENSMUSG00000033318	Gstt2	0.762916667	2.50E-05
pNF	ENSMUSG00000046561	Arsj	0.762878788	2.04E-05
pNF	ENSMUSG00000030598	Fbxo17	0.76280303	2.60E-05
pNF	ENSMUSG00000043342	Hoxd9	0.762424242	2.91E-05
pNF	ENSMUSG00000035773	Kiss1r	0.762045455	3.01E-05
pNF	ENSMUSG00000024851	Pitpnm1	0.761893939	3.14E-05
pNF	ENSMUSG00000041895	Wipi1	0.761515152	3.31E-05
pNF	ENSMUSG00000020253	Ppm1m	0.761363636	3.24E-05
pNF	ENSMUSG00000034098	Fstl5	0.761287879	3.40E-05

pNF	ENSMUSG00000038775	Vill	0.760681818	2.58E-05
pNF	ENSMUSG00000021265	Slc25a29	0.760606061	3.71E-05
pNF	ENSMUSG00000085241	Snhg3	0.760454545	3.76E-05
pNF	ENSMUSG00000039208	Metrnl	0.760265152	3.80E-05
pNF	ENSMUSG00000046167	Gldn	0.760189394	3.82E-05
pNF	ENSMUSG00000029333	Rasgef1b	0.759318182	4.41E-05
pNF	ENSMUSG00000020733	Slc9a3r1	0.759318182	4.42E-05
pNF	ENSMUSG00000006611	Hfe	0.758863636	4.57E-05
pNF	ENSMUSG00000074743	Thbd	0.7575	5.45E-05
pNF	ENSMUSG00000038524	Fchsd1	0.7575	5.62E-05
pNF	ENSMUSG00000028480	Glipr2	0.757121212	5.91E-05
pNF	ENSMUSG00000040043	Rbms2	0.75655303	6.32E-05
pNF	ENSMUSG00000086454	Platr14	0.756212121	6.12E-05
pNF	ENSMUSG00000029545	Acads	0.755492424	7.31E-05
pNF	ENSMUSG00000028082	Sh3d19	0.755378788	7.43E-05
pNF	ENSMUSG00000000308	Ckmt1	0.75530303	7.50E-05
pNF	ENSMUSG00000032172	Olfm2	0.75530303	7.46E-05
pNF	ENSMUSG00000007039	Ddah2	0.755151515	7.65E-05
pNF	ENSMUSG00000031682	1700011L22Rik	0.754583333	7.47E-05
pNF	ENSMUSG00000034107	Ano7	0.754545455	7.03E-05
pNF	ENSMUSG00000076441	Ass1	0.754318182	8.53E-05
pNF	ENSMUSG00000050321	Neto1	0.753484848	7.61E-05
pNF	ENSMUSG00000000753	Serpinf1	0.753333333	9.63E-05
pNF	ENSMUSG00000031684	Slc10a7	0.75280303	0.000103548
pNF	ENSMUSG00000042826	Fgf11	0.752765152	7.96E-05
pNF	ENSMUSG00000074673	Ttll9	0.752537879	0.000106441
pNF	ENSMUSG00000050671	Ism2	0.752159091	9.53E-05
pNF	ENSMUSG00000056144	Trim34a	0.752083333	9.46E-05
pNF	ENSMUSG00000020092	Pald1	0.751704545	0.00011458
pNF	ENSMUSG00000031952	Chst5	0.750833333	9.36E-05
pNF	ENSMUSG00000029171	Pgm1	0.750568182	0.000135819
pNF	ENSMUSG00000009876	Cox4i2	0.750378788	0.000133864
pNF	ENSMUSG00000042942	Greb1l	0.750151515	0.000145256
pNF	ENSMUSG00000029798	Herc6	0.750075758	0.000146428
pNF	ENSMUSG00000012443	Kif11	0.749734848	0.000147125
pNF	ENSMUSG00000046999	1110032F04Rik	0.74969697	0.000153988
pNF	ENSMUSG00000045555	Mettl24	0.749545455	0.000120105
pNF	ENSMUSG00000035686	Thrsp	0.749431818	0.000125527
pNF	ENSMUSG00000037977	6430571L13Rik	0.749128788	0.000158537
pNF	ENSMUSG00000068522	Aard	0.74905303	0.000149257
pNF	ENSMUSG00000041153	Osgin2	0.748939394	0.000169425
pNF	ENSMUSG00000043460	Elfn2	0.748598485	0.000150773
pNF	ENSMUSG00000032198	Dock6	0.748106061	0.000180884

pNF	ENSMUSG00000022678	Nde1	0.74780303	0.000185012
pNF	ENSMUSG00000031778	Cx3cl1	0.746287879	0.000220009
pNF	ENSMUSG00000003411	Rab3b	0.745984848	0.000247233
pNF	ENSMUSG00000034194	R3hcc1	0.745606061	0.0002592
pNF	ENSMUSG00000051890	Klhdc1	0.745378788	0.000265472
pNF	ENSMUSG00000022665	Ccdc80	0.744393939	0.000292095
pNF	ENSMUSG00000049649	Gpr3	0.744280303	0.000305013
pNF	ENSMUSG00000000340	Dbt	0.744090909	0.000312621
pNF	ENSMUSG00000036615	Rfxap	0.744015152	0.000316237
pNF	ENSMUSG00000035504	Reep6	0.743560606	0.000270873
pNF	ENSMUSG00000004933	Matk	0.743409091	0.000323638
pNF	ENSMUSG00000026638	Irf6	0.7425	0.000326256
pNF	ENSMUSG00000026525	Opn3	0.742083333	0.000401897
pNF	ENSMUSG00000038244	Mical2	0.742045455	0.000403331
pNF	ENSMUSG00000036995	Asap3	0.741931818	0.000392221
pNF	ENSMUSG00000033174	Mgll	0.741666667	0.000423633
pNF	ENSMUSG00000051502	Ufsp1	0.741439394	0.000434942
pNF	ENSMUSG00000006585	Cdt1	0.741287879	0.000357
pNF	ENSMUSG00000026433	Rab29	0.741098485	0.000442466
pNF	ENSMUSG00000040495	Chrm4	0.741022727	0.000436726
pNF	ENSMUSG00000020935	Dcakd	0.740833333	0.000468834
pNF	ENSMUSG00000068735	Trp53i11	0.740606061	0.000372683
pNF	ENSMUSG00000024430	Cabyr	0.740492424	0.000474271
pNF	ENSMUSG00000074925	Ptar1	0.740378788	0.000494855
pNF	ENSMUSG00000021647	Cartpt	0.739886364	0.000525892
pNF	ENSMUSG00000035228	Ccdc106	0.739734848	0.000530313
pNF	ENSMUSG00000070866	Zfp804a	0.739204545	0.000560563
pNF	ENSMUSG00000039168	Dap	0.738409091	0.000630147
pNF	ENSMUSG00000097059	NA	0.737992424	0.000625432
pNF	ENSMUSG00000023328	Ache	0.7375	0.000703292
pNF	ENSMUSG00000063018	NA	0.737007576	0.00069273
pNF	ENSMUSG00000026259	Ngef	0.736780303	0.00069839
pNF	ENSMUSG00000021791	Dydc2	0.73655303	0.000731519
pNF	ENSMUSG00000031482	Slc25a15	0.736439394	0.000763392
pNF	ENSMUSG00000009376	Met	0.736325758	0.00067548
pNF	ENSMUSG00000020814	Mxra7	0.735757576	0.000868599
pNF	ENSMUSG00000028523	Tctex1d1	0.73530303	0.000870106
pNF	ENSMUSG00000034308	Sdr42e1	0.735113636	0.000865494
pNF	ENSMUSG00000039497	Dse	0.734545455	0.000988845
pNF	ENSMUSG00000024736	Tmem132a	0.732575758	0.001266554
pNF	ENSMUSG00000020432	Tcn2	0.732575758	0.001265792
pNF	ENSMUSG00000021448	Shc3	0.7325	0.001090089
pNF	ENSMUSG00000089685	NA	0.732424242	0.000923339

pNF	ENSMUSG00000027171	Prrg4	0.73219697	0.001080641
pNF	ENSMUSG00000068105	Tnfrsf13c	0.732159091	0.001013022
pNF	ENSMUSG00000049866	Arl4c	0.732045455	0.001352251
pNF	ENSMUSG00000021668	Polk	0.731893939	0.001368121
pNF	ENSMUSG00000002475	Abhd3	0.73155303	0.001432237
pNF	ENSMUSG00000054204	Fam150b	0.731363636	0.001464833
pNF	ENSMUSG00000038987	Cfap157	0.731136364	0.001503957
pNF	ENSMUSG00000049148	Plcxd3	0.731060606	0.001519859
pNF	ENSMUSG00000027792	Bche	0.730757576	0.001560074
pNF	ENSMUSG00000025239	Limd1	0.73	0.001684642
pNF	ENSMUSG00000020766	Galk1	0.728787879	0.001983349
pNF	ENSMUSG00000073889	Il11ra1	0.728636364	0.002015597
pNF	ENSMUSG00000043843	Tmem145	0.728219697	0.002105674
pNF	ENSMUSG00000036158	Prickle1	0.728106061	0.001925463
pNF	ENSMUSG00000029552	Tes	0.727765152	0.002227096
pNF	ENSMUSG00000064346	NA	0.727234848	0.002159239
pNF	ENSMUSG00000014470	Rnf166	0.727234848	0.002371766
pNF	ENSMUSG00000073405	NA	0.727007576	0.002104646
pNF	ENSMUSG00000097769	Snhg4	0.726515152	0.002580348
pNF	ENSMUSG00000086181	NA	0.726363636	0.002606823
pNF	ENSMUSG00000035165	Kcne3	0.726060606	0.001977587
pNF	ENSMUSG00000031323	Dmrtc1a	0.725454545	0.002872719
pNF	ENSMUSG00000033854	Kcnk10	0.72469697	0.003153223
pNF	ENSMUSG00000002983	Relb	0.724659091	0.003177618
pNF	ENSMUSG00000062526	Mppe1	0.724621212	0.003209425
pNF	ENSMUSG00000052430	Bmpr1b	0.724431818	0.002934197
pNF	ENSMUSG00000048416	Mlf1	0.724166667	0.00337935
pNF	ENSMUSG00000031980	Agt	0.724166667	0.002803382
pNF	ENSMUSG00000037752	Xkr8	0.723636364	0.003516002
pNF	ENSMUSG00000048699	4732456N10Rik	0.723409091	0.003087689
pNF	ENSMUSG00000038990	Cables2	0.723409091	0.003666067
pNF	ENSMUSG00000032062	2310030G06Rik	0.723371212	0.002934649
pNF	ENSMUSG00000028197	Col24a1	0.723295455	0.003462051
pNF	ENSMUSG00000026489	Adck3	0.723181818	0.003783506
pNF	ENSMUSG00000032369	Plscr1	0.722840909	0.003371327
pNF	ENSMUSG00000029186	Pi4k2b	0.72280303	0.003907516
pNF	ENSMUSG00000050963	Kcns2	0.722575758	0.003294542
pNF	ENSMUSG00000028078	Dclk2	0.722462121	0.004104644
pNF	ENSMUSG00000042228	Lyn	0.722272727	0.00398558
pNF	ENSMUSG00000054178	NA	0.722159091	0.00398558
pNF	ENSMUSG00000037580	Gch1	0.722121212	0.004255937
pNF	ENSMUSG00000032177	Pde4a	0.722121212	0.00418573
pNF	ENSMUSG00000034771	Tle2	0.721363636	0.004621594

pNF	ENSMUSG00000031146	Plp2	0.720606061	0.005021754
pNF	ENSMUSG00000034825	Nrip3	0.720378788	0.005198098
pNF	ENSMUSG00000008318	Relt	0.720113636	0.005176707
pNF	ENSMUSG00000022676	Snai2	0.720075758	0.003815927
pNF	ENSMUSG00000048782	Insc	0.719659091	0.005602207
pNF	ENSMUSG00000038180	Spag4	0.719545455	0.005617517
pNF	ENSMUSG00000001901	Kcnh6	0.719318182	0.005854377
pNF	ENSMUSG00000032377	Plscr4	0.719090909	0.005915123
pNF	ENSMUSG00000079414	Gm11110	0.718939394	0.004584102
pNF	ENSMUSG00000060268	Gm1661	0.718825758	0.005565794
pNF	ENSMUSG00000020363	Gfpt2	0.717992424	0.006255816
pNF	ENSMUSG00000028494	Plin2	0.717575758	0.007120319
pNF	ENSMUSG00000040118	Cacna2d1	0.716590909	0.007943907
pNF	ENSMUSG00000038550	Ciart	0.715909091	0.008085525
pNF	ENSMUSG00000042340	Ctf1	0.715909091	0.006680123
pNF	ENSMUSG00000034265	Zdhhc14	0.715643939	0.008801949
pNF	ENSMUSG00000092274	NA	0.715113636	0.009266055
pNF	ENSMUSG00000010660	Plcd1	0.713333333	0.01132856
pNF	ENSMUSG00000032194	Kank2	0.713106061	0.010450141
pNF	ENSMUSG00000021303	Gng4	0.7125	0.012378388
pNF	ENSMUSG00000046750	BC089491	0.711856061	0.010078588
pNF	ENSMUSG00000024892	Pcx	0.711818182	0.013357981
pNF	ENSMUSG00000057176	Ccdc189	0.711666667	0.012601439
pNF	ENSMUSG00000002835	Chaf1a	0.711666667	0.012936094
pNF	ENSMUSG00000070802	Pnmal2	0.711590909	0.013630603
pNF	ENSMUSG00000024855	Pacs1	0.710795455	0.014886769
pNF	ENSMUSG00000051648	Kctd19	0.710643939	0.01282887
pNF	ENSMUSG00000003623	Crot	0.71030303	0.015728624
pNF	ENSMUSG00000021906	Oxnad1	0.709924242	0.016239234
pNF	ENSMUSG00000027459	Fam110a	0.709924242	0.015562656
pNF	ENSMUSG00000051998	Lax1	0.709886364	0.015688292
pNF	ENSMUSG00000074466	Gm15417	0.709772727	0.016510923
pNF	ENSMUSG00000086855	NA	0.709734848	0.015940822
pNF	ENSMUSG00000030030	1700003E16Rik	0.70969697	0.016777145
pNF	ENSMUSG00000046814	Gchfr	0.709659091	0.016379436
pNF	ENSMUSG00000070469	Adamtsl3	0.709242424	0.017491459
pNF	ENSMUSG00000045316	Fahd1	0.709242424	0.017603956
pNF	ENSMUSG00000036251	Trpm8	0.709128788	0.016236002
pNF	ENSMUSG00000090272	Mndal	0.709015152	0.016491012
pNF	ENSMUSG00000051341	Zfp52	0.708522727	0.018796846
pNF	ENSMUSG00000024906	Mus81	0.708333333	0.01933937
pNF	ENSMUSG00000026980	Ly75	0.7075	0.02112513
pNF	ENSMUSG00000031239	Itm2a	0.7075	0.020675696

pNF	ENSMUSG00000022453	Naga	0.70719697	0.02187552
pNF	ENSMUSG00000038393	Txnip	0.705492424	0.023820333
pNF	ENSMUSG00000030096	Slc6a6	0.705378788	0.026473737
pNF	ENSMUSG00000039620	6430573F11Rik	0.705378788	0.02619958
pNF	ENSMUSG00000033214	Slitrk5	0.705075758	0.027218015
pNF	ENSMUSG00000026295	Spp2	0.704128788	0.024518098
pNF	ENSMUSG00000086910	NA	0.703787879	0.024588295
pNF	ENSMUSG00000013155	Enkd1	0.703674242	0.031180255
pNF	ENSMUSG00000039131	Gipc2	0.703143939	0.032756637
pNF	ENSMUSG00000020828	Pld2	0.703030303	0.033372421
pNF	ENSMUSG00000040690	Col16a1	0.702840909	0.032528981
pNF	ENSMUSG00000035637	Grhpr	0.70280303	0.034374962
pNF	ENSMUSG00000030792	Dkkl1	0.702689394	0.026275367
pNF	ENSMUSG00000035834	Polr3g	0.7025	0.035708551
pNF	ENSMUSG00000028865	Cd164l2	0.702348485	0.03600464
pNF	ENSMUSG00000097666	NA	0.701969697	0.030531393
pNF	ENSMUSG00000040219	Ttc12	0.700454545	0.043717496
pNF	ENSMUSG00000050967	Creg2	0.699962121	0.038912869
pNF	ENSMUSG00000053040	Aph1c	0.699734848	0.046923824
pNF	ENSMUSG00000064344	NA	0.699431818	0.048878835
pNF	ENSMUSG00000048747	NA	0.698030303	0.049164781
mNFb	ENSMUSG00000022231	Sema5a	0.92843201	5.42E-19
mNFb	ENSMUSG00000026452	Syt2	0.913540085	1.75E-17
mNFb	ENSMUSG00000059187	Fam19a1	0.898503578	2.36E-16
mNFb	ENSMUSG00000025582	Nptx1	0.893226343	1.24E-15
mNFb	ENSMUSG00000071340	Trappc3l	0.892069688	1.77E-15
mNFb	ENSMUSG00000025867	Cplx2	0.888888889	3.55E-15
mNFb	ENSMUSG00000048251	Bcl11b	0.886792453	3.76E-15
mNFb	ENSMUSG00000053007	Creb5	0.878732018	1.77E-14
mNFb	ENSMUSG00000067586	S1pr3	0.878334418	3.06E-14
mNFb	ENSMUSG00000044734	Serpinb1a	0.868611292	1.86E-13
mNFb	ENSMUSG00000027217	Tspan18	0.868502855	2.06E-13
mNFb	ENSMUSG00000032667	Pon2	0.864490711	4.55E-13
mNFb	ENSMUSG00000064225	Paqr9	0.862538856	5.95E-13
mNFb	ENSMUSG00000052726	Kcnt2	0.858996602	6.95E-13
mNFb	ENSMUSG00000015354	Pcolce2	0.85877973	1.28E-12
mNFb	ENSMUSG00000042312	S100a13	0.858526711	1.50E-12
mNFb	ENSMUSG00000031565	Fgfr1	0.857370057	1.87E-12
mNFb	ENSMUSG00000032232	Cgnl1	0.857080894	1.73E-12
mNFb	ENSMUSG00000024497	Pou4f3	0.854225403	3.33E-12
mNFb	ENSMUSG00000006205	Htra1	0.854189258	3.38E-12
mNFb	ENSMUSG00000045625	Pigz	0.853972385	3.55E-12
mNFb	ENSMUSG00000021190	Lgmn	0.853972385	3.56E-12

mNFb	ENSMUSG00000059325	Hopx	0.846490277	1.29E-11
mNFb	ENSMUSG00000096956	Snhg18	0.845369768	1.33E-11
mNFb	ENSMUSG00000028161	Ppp3ca	0.844791441	1.97E-11
mNFb	ENSMUSG00000031906	Smpd3	0.843237186	2.62E-11
mNFb	ENSMUSG00000017446	C1qtnf1	0.840309405	4.45E-11
mNFb	ENSMUSG00000042834	Nrep	0.836550278	8.81E-11
mNFb	ENSMUSG00000017412	Cacnb4	0.835682788	1.03E-10
mNFb	ENSMUSG00000041565	L3mbtl4	0.83430926	8.54E-11
mNFb	ENSMUSG00000021730	Hcn1	0.834164679	1.34E-10
mNFb	ENSMUSG00000106019	NA	0.832899588	1.32E-10
mNFb	ENSMUSG00000008540	Mgst1	0.831309188	1.89E-10
mNFb	ENSMUSG00000029802	Abcg2	0.830405552	2.60E-10
mNFb	ENSMUSG00000040138	Ndp	0.830297116	2.35E-10
mNFb	ENSMUSG00000029869	Ephb6	0.828706716	3.31E-10
mNFb	ENSMUSG00000036578	Fxyd7	0.824983735	6.81E-10
mNFb	ENSMUSG00000028864	Hgf	0.824369262	6.06E-10
mNFb	ENSMUSG00000032698	Lmo2	0.82382708	5.65E-10
mNFb	ENSMUSG00000052934	Fbxo31	0.822670426	1.02E-09
mNFb	ENSMUSG00000047821	Trim16	0.822525844	9.77E-10
mNFb	ENSMUSG00000052534	Pbx1	0.821802935	1.18E-09
mNFb	ENSMUSG00000038894	Irs2	0.81818839	2.19E-09
mNFb	ENSMUSG00000020431	Adcy1	0.816236536	2.17E-09
mNFb	ENSMUSG00000050558	Prokr2	0.815911227	2.14E-09
mNFb	ENSMUSG00000074457	S100a16	0.814465409	3.62E-09
mNFb	ENSMUSG00000031749	St3gal2	0.813850936	4.54E-09
mNFb	ENSMUSG00000032243	Itga11	0.813489482	3.77E-09
mNFb	ENSMUSG00000034684	Sema3f	0.812694282	4.07E-09
mNFb	ENSMUSG00000018417	Myo1b	0.810923155	7.39E-09
mNFb	ENSMUSG00000100426	NA	0.810597846	4.61E-09
mNFb	ENSMUSG00000019889	Ptprk	0.809947228	5.15E-09
mNFb	ENSMUSG00000038700	Hoxb5	0.809007446	8.73E-09
mNFb	ENSMUSG00000028565	Nfia	0.806477265	1.54E-08
mNFb	ENSMUSG00000045288	Ush1g	0.805031447	1.56E-08
mNFb	ENSMUSG00000001773	Folh1	0.803404901	2.17E-08
mNFb	ENSMUSG00000005268	Prlr	0.802790429	2.09E-08
mNFb	ENSMUSG00000086712	AI427809	0.802428974	2.01E-08
mNFb	ENSMUSG00000037712	Fermt2	0.801200029	3.65E-08
mNFb	ENSMUSG00000016918	Sulf1	0.79928432	4.96E-08
mNFb	ENSMUSG00000078247	Airn	0.798959011	4.74E-08
mNFb	ENSMUSG00000095407	Tmem200c	0.796717993	5.40E-08
mNFb	ENSMUSG00000024553	Galr1	0.795669775	7.55E-08
mNFb	ENSMUSG00000021708	NA	0.793826357	1.13E-07
mNFb	ENSMUSG00000007030	Vwa7	0.793754066	1.19E-07

mNFb	ENSMUSG00000050587	Lrrc4c	0.792525121	1.42E-07
mNFb	ENSMUSG00000025969	Nrp2	0.790717849	1.92E-07
mNFb	ENSMUSG00000063594	Gng8	0.789633485	2.26E-07
mNFb	ENSMUSG00000026315	Serpinb8	0.787862358	2.95E-07
mNFb	ENSMUSG00000071537	Klrg2	0.787067158	3.07E-07
mNFb	ENSMUSG00000075254	Heg1	0.786488831	3.57E-07
mNFb	ENSMUSG00000074274	D930028M14Rik	0.784717704	4.49E-07
mNFb	ENSMUSG00000075408	6030408B16Rik	0.784645413	2.82E-07
mNFb	ENSMUSG00000071379	Hpcal1	0.784067086	5.35E-07
mNFb	ENSMUSG00000049001	Ndnf	0.782259813	4.92E-07
mNFb	ENSMUSG00000048458	Fam212b	0.782079086	6.80E-07
mNFb	ENSMUSG00000009216	Fam163b	0.780488686	6.05E-07
mNFb	ENSMUSG00000041607	Mbp	0.778934432	1.15E-06
mNFb	ENSMUSG00000026737	Pip4k2a	0.77878985	1.18E-06
mNFb	ENSMUSG00000079598	Clec2l	0.77878985	1.18E-06
mNFb	ENSMUSG00000052949	Rnf157	0.777488614	1.43E-06
mNFb	ENSMUSG00000001663	Gstt1	0.777416323	1.42E-06
mNFb	ENSMUSG00000031129	Slc9a9	0.774886142	2.11E-06
mNFb	ENSMUSG00000042831	Alkbh6	0.774090942	2.37E-06
mNFb	ENSMUSG00000046157	Tmem229b	0.771127015	3.64E-06
mNFb	ENSMUSG00000024998	Plce1	0.770151088	4.07E-06
mNFb	ENSMUSG00000050199	Lgr4	0.76950047	4.59E-06
mNFb	ENSMUSG00000028755	Cda	0.768560688	5.28E-06
mNFb	ENSMUSG00000030865	Chp2	0.767837779	4.53E-06
mNFb	ENSMUSG00000035735	Dagla	0.766066652	7.53E-06
mNFb	ENSMUSG00000027534	Snx16	0.763247307	1.12E-05
mNFb	ENSMUSG00000041020	Map7d2	0.762741271	1.21E-05
mNFb	ENSMUSG00000027351	Spred1	0.762741271	1.21E-05
mNFb	ENSMUSG00000085517	NA	0.762415962	9.60E-06
mNFb	ENSMUSG00000043017	Ptgir	0.761946071	1.35E-05
mNFb	ENSMUSG00000106644	NA	0.759668908	1.16E-05
mNFb	ENSMUSG00000021337	Scgn	0.758837562	1.93E-05
mNFb	ENSMUSG00000097313	NA	0.758150799	2.03E-05
mNFb	ENSMUSG00000050896	Rtn4rl2	0.757897781	2.15E-05
mNFb	ENSMUSG00000030042	Pole4	0.756668835	2.80E-05
mNFb	ENSMUSG00000026674	Ddr2	0.756596544	2.61E-05
mNFb	ENSMUSG00000043850	Clrn1	0.754933854	2.38E-05
mNFb	ENSMUSG00000031101	Sash3	0.754391672	2.64E-05
mNFb	ENSMUSG00000032860	P2ry2	0.75384949	3.56E-05
mNFb	ENSMUSG00000037406	Htra4	0.752114509	4.04E-05
mNFb	ENSMUSG00000026514	Cnih3	0.7513916	5.62E-05
mNFb	ENSMUSG00000009075	Cabp7	0.751030145	3.97E-05
mNFb	ENSMUSG00000005994	Tyrp1	0.749728909	5.98E-05

mNFb	ENSMUSG00000047040	Prr15l	0.749439746	5.54E-05
mNFb	ENSMUSG00000019891	Dcbld1	0.748427673	8.53E-05
mNFb	ENSMUSG00000020614	Fam20a	0.747740909	9.11E-05
mNFb	ENSMUSG00000105974	NA	0.746764982	7.60E-05
mNFb	ENSMUSG00000049521	Cdc42ep1	0.746439673	8.79E-05
mNFb	ENSMUSG00000041417	Pik3r1	0.745680619	0.000122719
mNFb	ENSMUSG00000057816	1700007G11Rik	0.745572182	9.67E-05
mNFb	ENSMUSG00000025931	Paqr8	0.745102292	0.000132376
mNFb	ENSMUSG00000022098	Bmp1	0.744668546	0.000113696
mNFb	ENSMUSG00000020823	Sec14l1	0.742933565	0.000175623
mNFb	ENSMUSG00000106321	NA	0.742861274	0.000138747
mNFb	ENSMUSG00000042581	Thsd7b	0.742427528	0.000170477
mNFb	ENSMUSG00000038295	Atg9b	0.742029928	0.000164305
mNFb	ENSMUSG00000027999	Pla2g12a	0.741198583	0.000219891
mNFb	ENSMUSG00000020900	Myh10	0.738234656	0.000321109
mNFb	ENSMUSG00000049336	Tenm2	0.737692475	0.000341698
mNFb	ENSMUSG00000025887	Casp12	0.737403311	0.000339213
mNFb	ENSMUSG00000023047	Amhr2	0.737150293	0.000319283
mNFb	ENSMUSG00000047881	Rell1	0.735126148	0.000464415
mNFb	ENSMUSG00000007944	Ttc9b	0.734692402	0.000502816
mNFb	ENSMUSG00000042271	Nxt2	0.73407793	0.000543019
mNFb	ENSMUSG00000062098	Btbd3	0.733391166	0.000591711
mNFb	ENSMUSG00000028033	Kcnq5	0.732270657	0.000669028
mNFb	ENSMUSG00000052125	F730043M19Rik	0.730644112	0.000748414
mNFb	ENSMUSG00000040483	Xaf1	0.72970433	0.000932971
mNFb	ENSMUSG00000034463	Scara3	0.728583821	0.000822035
mNFb	ENSMUSG00000068037	Mas1	0.726776549	0.001028885
mNFb	ENSMUSG00000019947	Arid5b	0.72565604	0.001523777
mNFb	ENSMUSG00000094002	Gm9866	0.724680113	0.001670475
mNFb	ENSMUSG00000045201	Lrrc3b	0.723451167	0.001993353
mNFb	ENSMUSG00000031628	Casp3	0.722764404	0.00216104
mNFb	ENSMUSG00000031827	Cotl1	0.721933059	0.002234482
mNFb	ENSMUSG00000029561	Oasl2	0.721571604	0.002495434
mNFb	ENSMUSG00000069662	Marcks	0.721463168	0.002525088
mNFb	ENSMUSG00000050947	Amigo1	0.721318586	0.002570917
mNFb	ENSMUSG00000052155	Acvr2a	0.720740259	0.002751699
mNFb	ENSMUSG00000006411	Pvrl4	0.720053495	0.002915629
mNFb	ENSMUSG00000031659	Adcy7	0.719583604	0.002956371
mNFb	ENSMUSG00000104938	NA	0.719186004	0.002945928
mNFb	ENSMUSG00000026031	Cflar	0.718499241	0.003577759
mNFb	ENSMUSG00000022075	Rhobtb2	0.717125714	0.004192084
mNFb	ENSMUSG00000019737	Syne4	0.717017278	0.003594351
mNFb	ENSMUSG00000032024	Clmp	0.716330514	0.004590541

mNFb	ENSMUSG00000032060	Cryab	0.715969059	0.00475975
mNFb	ENSMUSG00000038776	Ephx1	0.715896769	0.004837161
mNFb	ENSMUSG00000019856	Fam184a	0.715463023	0.00490431
mNFb	ENSMUSG00000049686	Orai1	0.715246151	0.005207507
mNFb	ENSMUSG00000085042	Abhd11os	0.714342514	0.005032651
mNFb	ENSMUSG00000043259	Fam13c	0.712860551	0.006846476
mNFb	ENSMUSG00000081433	NA	0.712246078	0.005975907
mNFb	ENSMUSG00000027630	Tbl1xr1	0.712065351	0.00751373
mNFb	ENSMUSG00000054364	Rhob	0.710872551	0.008599528
mNFb	ENSMUSG00000030806	Stx1b	0.710764115	0.008699611
mNFb	ENSMUSG00000032462	Pik3cb	0.710077351	0.009388643
mNFb	ENSMUSG00000086363	A330102I10Rik	0.70960746	0.009898912
mNFb	ENSMUSG00000040253	Gbp7	0.708884551	0.010732846
mNFb	ENSMUSG00000029765	Plxna4	0.708595388	0.011092742
mNFb	ENSMUSG00000097125	NA	0.708559242	0.009039778
mNFb	ENSMUSG00000026213	Stk11ip	0.707908624	0.011977618
mNFb	ENSMUSG00000024588	Fech	0.707836333	0.012076018
mNFb	ENSMUSG00000027074	Slc43a3	0.707511024	0.012159546
mNFb	ENSMUSG00000026826	Nr4a2	0.706390515	0.013695101
mNFb	ENSMUSG00000031596	Slc7a2	0.705306152	0.015314021
mNFb	ENSMUSG00000038192	Cer1	0.705233861	0.013678697
mNFb	ENSMUSG00000071547	Nt5dc2	0.705233861	0.012399699
mNFb	ENSMUSG00000029249	Rest	0.704149498	0.017321522
mNFb	ENSMUSG00000020108	Ddit4	0.704041061	0.018297383
mNFb	ENSMUSG00000028967	Errfi1	0.703860334	0.018670293
mNFb	ENSMUSG00000054733	Msra	0.70357117	0.019196159
mNFb	ENSMUSG00000031133	Arhgef6	0.703462734	0.019477702
mNFb	ENSMUSG00000031284	Pak3	0.703390443	0.019648368
mNFb	ENSMUSG00000029167	Ppargc1a	0.702378371	0.021786299
mNFb	ENSMUSG00000050730	Arhgap42	0.701655462	0.023677461
mNFb	ENSMUSG00000071753	C230004F18Rik	0.701257862	0.024756055
mNFb	ENSMUSG00000071637	NA	0.700968698	0.025478441
mNFb	ENSMUSG00000096257	Ccer2	0.699812044	0.022103998
mNFb	ENSMUSG00000028708	Mknk1	0.699378298	0.030286342
mNFb	ENSMUSG00000037010	Apln	0.699342153	0.027793808
mNFb	ENSMUSG00000033420	Antxr1	0.698908407	0.030765462
mNFb	ENSMUSG00000020282	Rhbdf1	0.698583098	0.031649003
mNFb	ENSMUSG00000086605	NA	0.698510807	0.03315932
mNFb	ENSMUSG00000031775	Pllp	0.696811971	0.03672624
mNFb	ENSMUSG00000027457	Snph	0.69673968	0.040018553
mNFb	ENSMUSG00000025938	Slco5a1	0.696703535	0.039683297
mNFb	ENSMUSG00000024771	Lipk	0.696631244	0.035434771
mNFb	ENSMUSG00000029449	Rhof	0.696522808	0.040941382

mNFb	ENSMUSG00000030922	Lyrm1	0.694932408	0.048418475
mNFb	ENSMUSG00000041308	Sntb2	0.694643244	0.049821645
mNFb	ENSMUSG00000083355	NA	0.694462517	0.040165107
mPEPa	ENSMUSG00000022342	Kcnv1	0.929152149	1.19E-09
mPEPa	ENSMUSG00000038370	Pcp4l1	0.926054975	1.78E-09
mPEPa	ENSMUSG00000049946	BC030500	0.914698671	3.87E-09
mPEPa	ENSMUSG00000029281	Smr2	0.899019228	2.68E-08
mPEPa	ENSMUSG00000058057	Mettl7a3	0.892115112	1.24E-07
mPEPa	ENSMUSG00000028073	Pear1	0.883662408	2.57E-07
mPEPa	ENSMUSG00000105807	NA	0.869725126	8.39E-07
mPEPa	ENSMUSG00000025576	Rbfox3	0.856884759	6.50E-06
mPEPa	ENSMUSG00000021721	Htr1a	0.844367015	1.66E-05
mPEPa	ENSMUSG00000041831	Sytl3	0.840366499	3.64E-05
mPEPa	ENSMUSG00000057715	A830018L16Rik	0.838946961	4.21E-05
mPEPa	ENSMUSG00000053519	Kcnip1	0.835527165	5.73E-05
mPEPa	ENSMUSG00000038276	Asic3	0.828881146	9.28E-05
mPEPa	ENSMUSG00000037628	Cdkn3	0.825267776	0.000164299
mPEPa	ENSMUSG00000035547	Capn5	0.821783456	0.000230651
mPEPa	ENSMUSG00000027858	Tspan2	0.820492967	0.000261353
mPEPa	ENSMUSG00000037318	Traf3ip3	0.8195251	0.000274506
mPEPa	ENSMUSG00000020519	Sap30l	0.815718157	0.000412268
mPEPa	ENSMUSG00000028072	Ntrk1	0.815201962	0.000432424
mPEPa	ENSMUSG00000020186	Csrp2	0.815201962	0.000432613
mPEPa	ENSMUSG00000027499	Pkia	0.814556717	0.000459789
mPEPa	ENSMUSG00000069135	Fgfr1op	0.814298619	0.000470754
mPEPa	ENSMUSG00000030525	Chrna7	0.813459801	0.000465275
mPEPa	ENSMUSG00000054162	Spock3	0.81300813	0.000530716
mPEPa	ENSMUSG00000041544	Ptchd2	0.808362369	0.000768185
mPEPa	ENSMUSG00000022951	Rcan1	0.80707188	0.000921504
mPEPa	ENSMUSG00000059456	Ptk2b	0.805781391	0.000903412
mPEPa	ENSMUSG00000070476	Fam217b	0.804232804	0.001194454
mPEPa	ENSMUSG00000029245	Epha5	0.803329462	0.001292874
mPEPa	ENSMUSG00000105576	NA	0.803264937	0.000917443
mPEPa	ENSMUSG00000004151	Etv1	0.800813008	0.00162361
mPEPa	ENSMUSG00000052302	Tbc1d30	0.800103239	0.001737531
mPEPa	ENSMUSG00000036181	Hist1h1c	0.799845141	0.001778713
mPEPa	ENSMUSG00000018909	Arrb1	0.799199897	0.001885258
mPEPa	ENSMUSG00000049511	Htr1b	0.798877274	0.001894842
mPEPa	ENSMUSG00000045519	Zfp560	0.796877016	0.002319645
mPEPa	ENSMUSG00000022237	Ankrd33b	0.795973674	0.001983349
mPEPa	ENSMUSG00000031937	Vstm5	0.795586527	0.002600857
mPEPa	ENSMUSG00000066720	Cldn9	0.787972642	0.003817242
mPEPa	ENSMUSG00000050663	Trhde	0.787650019	0.004298709

mPEPa	ENSMUSG00000022257	Laptm4b	0.78577881	0.006104094
mPEPa	ENSMUSG00000106065	NA	0.784746419	0.004585465
mPEPa	ENSMUSG00000037653	Kctd8	0.78461737	0.006745409
mPEPa	ENSMUSG00000015882	Lcorl	0.783843077	0.007207972
mPEPa	ENSMUSG00000037553	Zdhhc18	0.782810685	0.007870996
mPEPa	ENSMUSG00000037705	Tecta	0.781971867	0.008368959
mPEPa	ENSMUSG00000030323	Ift122	0.779971609	0.009993869
mPEPa	ENSMUSG00000025723	Nmb	0.779455414	0.010432782
mPEPa	ENSMUSG00000030653	Pde2a	0.776229191	0.013639833
mPEPa	ENSMUSG00000025408	Ddit3	0.775842044	0.014084011
mPEPa	ENSMUSG00000075232	Amd1	0.772873919	0.017952054
mPEPa	ENSMUSG00000027994	Ccdc109b	0.772486772	0.018523616
mPEPa	ENSMUSG00000022656	Pvrl3	0.772099626	0.019116024
mPEPa	ENSMUSG00000008575	Nfib	0.771454381	0.020125272
mPEPa	ENSMUSG00000051177	Plcb1	0.76926055	0.024067148
mPEPa	ENSMUSG00000029651	Mtus2	0.76809911	0.026427035
mPEPa	ENSMUSG00000096025	NA	0.766421474	0.030251977
mPEPa	ENSMUSG00000039717	Ralyl	0.766034327	0.031180255
mPEPa	ENSMUSG00000078713	Tomm5	0.765776229	0.031817942
mPEPa	ENSMUSG00000042743	Sgtb	0.765776229	0.031817942
mPEPa	ENSMUSG00000019464	Ptger1	0.765518131	0.032466543
mPEPa	ENSMUSG00000021596	Mctp1	0.765260034	0.03055283
mPEPa	ENSMUSG00000021640	Naip1	0.764679313	0.030352616
mPEPa	ENSMUSG00000021071	Trim9	0.763711447	0.037433377
mPEPa	ENSMUSG00000024059	Clip4	0.762550006	0.041057851
mPEPa	ENSMUSG00000028226	Mmp16	0.761388566	0.045006755
mPEPa	ENSMUSG00000031202	Rab39b	0.760485224	0.048353749
mPEPa	ENSMUSG00000039323	Igfbp2	0.759839979	0.048557868
mNP	ENSMUSG00000067714	Lpar5	1	2.52E-07
mNP	ENSMUSG00000069372	Ctxn3	1	3.10E-07
mNP	ENSMUSG00000026322	Htr4	0.999777035	3.23E-07
mNP	ENSMUSG00000041737	Tmem45b	0.999331104	3.74E-07
mNP	ENSMUSG00000023903	Mmp25	0.998885173	3.45E-07
mNP	ENSMUSG00000009378	Slc16a12	0.998885173	3.09E-07
mNP	ENSMUSG00000025221	Kcnip2	0.998662207	4.19E-07
mNP	ENSMUSG00000027716	Trpc3	0.998439242	4.87E-07
mNP	ENSMUSG00000087014	NA	0.997993311	3.70E-07
mNP	ENSMUSG00000056296	Synpr	0.997993311	5.58E-07
mNP	ENSMUSG00000054920	Klhl5	0.997770346	5.42E-07
mNP	ENSMUSG00000093502	NA	0.996432553	5.96E-07
mNP	ENSMUSG00000038903	Ccdc68	0.995986622	3.47E-07
mNP	ENSMUSG00000028794	A3galt2	0.99509476	6.91E-07
mNP	ENSMUSG00000099560	NA	0.99509476	5.56E-07

mNP	ENSMUSG00000032561	Acpp	0.994202899	7.89E-07
mNP	ENSMUSG00000022935	Grik1	0.993756968	7.67E-07
mNP	ENSMUSG00000031997	Trpc6	0.993534002	7.81E-07
mNP	ENSMUSG00000032278	Paqr5	0.993311037	7.94E-07
mNP	ENSMUSG00000036198	Arhgap36	0.991973244	6.99E-07
mNP	ENSMUSG00000074785	Plxnc1	0.990635452	1.07E-06
mNP	ENSMUSG00000054457	NA	0.990412486	6.03E-07
mNP	ENSMUSG00000001420	Tmem79	0.989966555	1.15E-06
mNP	ENSMUSG00000027654	Fam83d	0.989520624	7.97E-07
mNP	ENSMUSG00000007655	Cav1	0.988851728	7.24E-07
mNP	ENSMUSG00000031517	Gpm6a	0.988628763	1.11E-06
mNP	ENSMUSG00000086915	NA	0.988405797	1.20E-06
mNP	ENSMUSG00000061859	Inadl	0.987736901	1.32E-06
mNP	ENSMUSG00000005148	Klf5	0.987513935	1.20E-06
mNP	ENSMUSG00000024172	St6gal2	0.987513935	7.50E-07
mNP	ENSMUSG00000061126	Cyp4f39	0.98729097	8.75E-07
mNP	ENSMUSG00000024697	Gna14	0.987068004	1.27E-06
mNP	ENSMUSG00000029088	Kcnip4	0.986845039	1.50E-06
mNP	ENSMUSG00000032420	Nt5e	0.986622074	1.42E-06
mNP	ENSMUSG00000036062	Phf24	0.986399108	1.56E-06
mNP	ENSMUSG00000021852	Slc35f4	0.986399108	1.49E-06
mNP	ENSMUSG00000035954	Dock4	0.985061315	1.53E-06
mNP	ENSMUSG00000053141	Ptprt	0.98483835	1.52E-06
mNP	ENSMUSG00000029185	Fam114a1	0.984615385	1.63E-06
mNP	ENSMUSG00000026519	Tmem63a	0.983054627	1.68E-06
mNP	ENSMUSG00000022490	Ppp1r1a	0.981939799	1.73E-06
mNP	ENSMUSG00000079278	Tmem233	0.981047938	2.08E-06
mNP	ENSMUSG00000032812	Arap1	0.980824972	2.45E-06
mNP	ENSMUSG00000039197	Adk	0.980379041	2.58E-06
mNP	ENSMUSG00000027016	Zfp385b	0.980156076	2.44E-06
mNP	ENSMUSG00000024511	Rab27b	0.97993311	2.63E-06
mNP	ENSMUSG00000021423	Ly86	0.979710145	2.72E-06
mNP	ENSMUSG00000026399	Cd55	0.979487179	2.78E-06
mNP	ENSMUSG00000032380	Dapk2	0.979041249	2.48E-06
mNP	ENSMUSG00000091722	Siah3	0.978818283	1.97E-06
mNP	ENSMUSG00000062151	Unc13c	0.978818283	2.13E-06
mNP	ENSMUSG00000071489	Ptgdr	0.978149387	2.71E-06
mNP	ENSMUSG00000070551	Mrgprb5	0.976923077	2.22E-06
mNP	ENSMUSG00000027071	P2rx3	0.975919732	3.72E-06
mNP	ENSMUSG00000017588	Krt27	0.975250836	2.53E-06
mNP	ENSMUSG00000067786	Nnat	0.974804905	3.78E-06
mNP	ENSMUSG00000074968	Ano3	0.974136009	4.32E-06
mNP	ENSMUSG00000038872	Zfhx3	0.973467113	4.33E-06

mNP	ENSMUSG00000097768	NA	0.972575251	3.16E-06
mNP	ENSMUSG00000024620	Pdgfrb	0.972575251	3.80E-06
mNP	ENSMUSG00000020000	Moxd1	0.972352285	3.13E-06
mNP	ENSMUSG00000024558	Mapk4	0.972352285	4.67E-06
mNP	ENSMUSG00000027347	Rasgrp1	0.971014493	5.52E-06
mNP	ENSMUSG00000043110	Lrrn4	0.970122631	3.61E-06
mNP	ENSMUSG00000027215	Cd82	0.969899666	6.08E-06
mNP	ENSMUSG00000051920	Rspo2	0.969899666	5.58E-06
mNP	ENSMUSG00000034115	Scn11a	0.969453735	6.33E-06
mNP	ENSMUSG00000058740	Kcnt1	0.967001115	7.73E-06
mNP	ENSMUSG00000037735	NA	0.967001115	6.18E-06
mNP	ENSMUSG00000045404	Kcnk13	0.967001115	6.56E-06
mNP	ENSMUSG00000081225	Cyp2j12	0.966778149	5.15E-06
mNP	ENSMUSG00000002504	Slc9a3r2	0.965663322	8.61E-06
mNP	ENSMUSG00000049409	Prokr1	0.964994426	6.16E-06
mNP	ENSMUSG00000026778	Prkcq	0.964102564	6.53E-06
mNP	ENSMUSG00000034533	Scn10a	0.963433668	1.03E-05
mNP	ENSMUSG00000048442	Smim5	0.962987737	9.04E-06
mNP	ENSMUSG00000037824	Tspan14	0.962541806	1.11E-05
mNP	ENSMUSG00000055805	Fmnl1	0.960089186	1.34E-05
mNP	ENSMUSG00000054469	Lclat1	0.959197324	1.45E-05
mNP	ENSMUSG00000097789	Gm2115	0.958751394	1.37E-05
mNP	ENSMUSG00000020592	Sdc1	0.958751394	1.39E-05
mNP	ENSMUSG00000026023	Cdk15	0.958528428	1.23E-05
mNP	ENSMUSG00000061184	Tmprss11c	0.958305463	9.75E-06
mNP	ENSMUSG00000036306	Lzts1	0.958305463	1.49E-05
mNP	ENSMUSG00000106554	NA	0.957859532	1.28E-05
mNP	ENSMUSG00000023232	Serinc2	0.957413601	1.61E-05
mNP	ENSMUSG00000001288	Rarg	0.957190635	1.59E-05
mNP	ENSMUSG00000034898	Filip1	0.956075808	1.84E-05
mNP	ENSMUSG00000020262	Adarb1	0.955629877	1.80E-05
mNP	ENSMUSG00000030768	Disp1	0.955406912	1.89E-05
mNP	ENSMUSG00000049420	Tmem200a	0.955183946	1.75E-05
mNP	ENSMUSG00000022103	Gfra2	0.952731327	2.09E-05
mNP	ENSMUSG00000056313	1810011O10Rik	0.951839465	1.91E-05
mNP	ENSMUSG00000028977	Casz1	0.951616499	2.56E-05
mNP	ENSMUSG00000056492	Adgrf5	0.951170569	1.93E-05
mNP	ENSMUSG00000052544	St6galnac3	0.950724638	2.71E-05
mNP	ENSMUSG00000101860	NA	0.950724638	2.58E-05
mNP	ENSMUSG00000054728	Phactr1	0.950278707	2.92E-05
mNP	ENSMUSG00000008393	Carhsp1	0.950055741	2.97E-05
mNP	ENSMUSG00000028487	Bnc2	0.948272018	3.13E-05
mNP	ENSMUSG00000028367	Txn1	0.948049052	3.48E-05

mNP	ENSMUSG00000034990	Otoa	0.948049052	2.79E-05
mNP	ENSMUSG00000026527	Rgs7	0.947826087	3.53E-05
mNP	ENSMUSG00000025429	Pstpip2	0.947826087	3.31E-05
mNP	ENSMUSG00000026765	Lypd6b	0.947268673	2.94E-05
mNP	ENSMUSG00000002908	Kcnn1	0.947157191	3.68E-05
mNP	ENSMUSG00000034485	Uaca	0.947157191	3.70E-05
mNP	ENSMUSG00000000154	Slc22a18	0.946934225	3.34E-05
mNP	ENSMUSG00000030905	Crym	0.94671126	3.52E-05
mNP	ENSMUSG00000030917	Tmem159	0.945150502	3.82E-05
mNP	ENSMUSG00000036304	Zdhhc23	0.944481605	3.64E-05
mNP	ENSMUSG00000000303	Cdh1	0.94425864	4.26E-05
mNP	ENSMUSG00000062309	Rpp25	0.944035674	3.94E-05
mNP	ENSMUSG00000039621	Prex1	0.942920847	4.97E-05
mNP	ENSMUSG00000023224	Serping1	0.941471572	5.68E-05
mNP	ENSMUSG00000031668	Eif2ak3	0.941360089	5.75E-05
mNP	ENSMUSG00000025572	Tmc6	0.941137124	5.28E-05
mNP	ENSMUSG00000040907	Atp1a3	0.940245262	6.34E-05
mNP	ENSMUSG00000033453	Adamts15	0.939576366	5.46E-05
mNP	ENSMUSG00000042671	Rgs8	0.939130435	6.78E-05
mNP	ENSMUSG00000058881	Zfp516	0.939130435	6.35E-05
mNP	ENSMUSG00000046546	Fam43a	0.938907469	4.74E-05
mNP	ENSMUSG00000024743	Syt7	0.938684504	7.14E-05
mNP	ENSMUSG00000055653	Gpc3	0.938461538	4.50E-05
mNP	ENSMUSG00000021559	Dapk1	0.938461538	7.26E-05
mNP	ENSMUSG00000036832	Lpar3	0.938350056	5.36E-05
mNP	ENSMUSG00000025813	Homer2	0.93690078	7.62E-05
mNP	ENSMUSG00000039682	Lap3	0.936454849	8.46E-05
mNP	ENSMUSG00000058656	Samd12	0.935785953	8.87E-05
mNP	ENSMUSG00000021118	Plek2	0.934671126	8.40E-05
mNP	ENSMUSG00000059810	Rgs3	0.934448161	9.84E-05
mNP	ENSMUSG00000030844	Rgs10	0.93400223	0.000101758
mNP	ENSMUSG00000050138	Kcnk12	0.93400223	0.000100808
mNP	ENSMUSG00000027669	Gnb4	0.933333333	0.000107031
mNP	ENSMUSG00000039037	St6galnac5	0.933221851	9.20E-05
mNP	ENSMUSG00000050965	Prkca	0.932887402	0.000110691
mNP	ENSMUSG00000055865	Fam19a3	0.932664437	7.71E-05
mNP	ENSMUSG00000002997	Prkar2b	0.932664437	0.000112544
mNP	ENSMUSG00000035095	Fam167a	0.932329989	7.64E-05
mNP	ENSMUSG00000031994	Adamts8	0.928316611	0.000120918
mNP	ENSMUSG00000035934	Pknox2	0.928205128	0.000154656
mNP	ENSMUSG00000030350	Prmt8	0.926867336	0.000149204
mNP	ENSMUSG00000046500	Fam19a4	0.92664437	0.000130838
mNP	ENSMUSG00000021998	Lcp1	0.92664437	0.000175926

mNP	ENSMUSG00000038530	Rgs4	0.926198439	0.000182093
mNP	ENSMUSG00000045932	Ifit2	0.925529543	0.000190872
mNP	ENSMUSG00000047409	Ctdspl	0.925529543	0.000185746
mNP	ENSMUSG00000042082	Arsb	0.925529543	0.000191077
mNP	ENSMUSG00000029174	Tbc1d1	0.924860647	0.000190302
mNP	ENSMUSG00000090667	Gm765	0.924860647	0.000129822
mNP	ENSMUSG00000047557	Lxn	0.923522854	0.000221815
mNP	ENSMUSG00000038665	Dgki	0.923522854	0.000221815
mNP	ENSMUSG00000050447	Lypd6	0.923299889	0.00014996
mNP	ENSMUSG00000051159	Cited1	0.923076923	0.000186703
mNP	ENSMUSG00000055737	Ghr	0.923076923	0.000228411
mNP	ENSMUSG00000022747	St3gal6	0.923076923	0.000228925
mNP	ENSMUSG00000040901	Kcnk18	0.922630992	0.000184413
mNP	ENSMUSG00000035184	Fam124a	0.920847269	0.000261672
mNP	ENSMUSG00000056158	Car10	0.920624303	0.00026684
mNP	ENSMUSG00000042215	Bag2	0.919509476	0.000297522
mNP	ENSMUSG00000024063	Lbh	0.916833891	0.000360954
mNP	ENSMUSG00000044667	Plppr4	0.91638796	0.000354741
mNP	ENSMUSG00000030769	Slc5a11	0.915942029	0.000302078
mNP	ENSMUSG00000024960	Plcb3	0.915719064	0.000392148
mNP	ENSMUSG00000036242	3632451O06Rik	0.915496098	0.000398468
mNP	ENSMUSG00000026655	Fam107b	0.915273133	0.000404693
mNP	ENSMUSG00000045095	Magi1	0.915273133	0.000404693
mNP	ENSMUSG00000020868	Xylt2	0.915050167	0.000403331
mNP	ENSMUSG00000021981	Cab39l	0.914381271	0.000431738
mNP	ENSMUSG00000037071	Scd1	0.914381271	0.000431738
mNP	ENSMUSG00000021822	Plau	0.913712375	0.000313311
mNP	ENSMUSG00000032220	Myo1e	0.913266444	0.000467117
mNP	ENSMUSG00000038332	Sesn1	0.913043478	0.000473393
mNP	ENSMUSG00000056947	Mab21l1	0.912931996	0.000316767
mNP	ENSMUSG00000062184	Hs6st2	0.912374582	0.000487847
mNP	ENSMUSG00000020961	Ston2	0.912151616	0.000496067
mNP	ENSMUSG00000024544	Ldlrad4	0.911705686	0.000434365
mNP	ENSMUSG00000032012	Pvrl1	0.911705686	0.000488169
mNP	ENSMUSG00000009731	Kcnd1	0.911259755	0.000538399
mNP	ENSMUSG00000046223	Plaur	0.911036789	0.000536143
mNP	ENSMUSG00000033767	D930015E06Rik	0.910813824	0.000553433
mNP	ENSMUSG00000048756	Foxo3	0.910144928	0.000582902
mNP	ENSMUSG00000032593	Amigo3	0.910144928	0.000582272
mNP	ENSMUSG00000029669	Tspan12	0.909921962	0.000550501
mNP	ENSMUSG00000024479	Mal2	0.909921962	0.000585562
mNP	ENSMUSG00000063142	Kcnma1	0.908807135	0.000637527
mNP	ENSMUSG00000052539	Magi3	0.908361204	0.000661933

mNP	ENSMUSG00000027375	Mal	0.907692308	0.000668491
mNP	ENSMUSG00000022377	Asap1	0.907023411	0.000727567
mNP	ENSMUSG00000047139	Cd24a	0.905685619	0.000799446
mNP	ENSMUSG00000069833	Ahnak	0.905685619	0.000796966
mNP	ENSMUSG00000021948	Prkcd	0.905462653	0.000812198
mNP	ENSMUSG00000031093	Dock11	0.904347826	0.000878129
mNP	ENSMUSG00000071604	Fam189a2	0.904347826	0.000844051
mNP	ENSMUSG00000035778	Ggta1	0.904347826	0.000866299
mNP	ENSMUSG00000041120	Nbl1	0.904124861	0.000890927
mNP	ENSMUSG00000038417	Fig4	0.904124861	0.000891829
mNP	ENSMUSG00000086040	Wipf3	0.903901895	0.000884764
mNP	ENSMUSG00000040430	Pitpnc1	0.903455964	0.00093437
mNP	ENSMUSG00000040234	Tm7sf3	0.902787068	0.000977177
mNP	ENSMUSG00000060924	Csmd1	0.902787068	0.000977291
mNP	ENSMUSG00000045312	Lhfpl2	0.901895206	0.000946437
mNP	ENSMUSG00000035268	Pkig	0.901449275	0.001074948
mNP	ENSMUSG00000020642	Rnf144a	0.90122631	0.001090232
mNP	ENSMUSG00000074111	Mrgpra9	0.901003344	0.000946449
mNP	ENSMUSG00000050335	Lgals3	0.900334448	0.001075072
mNP	ENSMUSG00000022952	Runx1	0.900111483	0.001178875
mNP	ENSMUSG00000086122	NA	0.899442586	0.001110806
mNP	ENSMUSG00000030123	Plxnd1	0.899442586	0.00123105
mNP	ENSMUSG00000104330	NA	0.899442586	0.001105524
mNP	ENSMUSG00000040549	Ckap5	0.898996656	0.00127421
mNP	ENSMUSG00000057531	Dtnbp1	0.898550725	0.001314161
mNP	ENSMUSG00000026828	Galnt5	0.898327759	0.001322679
mNP	ENSMUSG00000004270	Lpcat3	0.898104794	0.001354278
mNP	ENSMUSG00000024589	Nedd4l	0.897435897	0.001419505
mNP	ENSMUSG00000033578	Tmem35	0.897324415	0.001359662
mNP	ENSMUSG00000074238	Ap1ar	0.895429208	0.001630284
mNP	ENSMUSG00000004952	Rasa4	0.895429208	0.001561929
mNP	ENSMUSG00000010505	Myt1	0.895206243	0.001648941
mNP	ENSMUSG00000025432	Avil	0.894760312	0.001705895
mNP	ENSMUSG00000033470	Cysltr2	0.89386845	0.001536239
mNP	ENSMUSG00000020696	Rffl	0.89386845	0.001804305
mNP	ENSMUSG00000026880	Stom	0.893199554	0.001894842
mNP	ENSMUSG00000028952	Zbtb48	0.892976589	0.001897416
mNP	ENSMUSG00000096982	NA	0.892865106	0.001645656
mNP	ENSMUSG00000021217	Tshz3	0.892084727	0.002038193
mNP	ENSMUSG00000030930	Chst15	0.891861761	0.002024189
mNP	ENSMUSG00000020155	Kcnmb1	0.891861761	0.002067803
mNP	ENSMUSG00000104610	NA	0.8909699	0.001855459
mNP	ENSMUSG00000040136	Abcc8	0.8909699	0.001720801

mNP	ENSMUSG00000037188	Grhl3	0.890412486	0.001902015
mNP	ENSMUSG00000061887	Ssbp3	0.890301003	0.002312599
mNP	ENSMUSG00000041608	Entpd3	0.890301003	0.002312027
mNP	ENSMUSG00000034226	Rhov	0.890078038	0.002347303
mNP	ENSMUSG00000058589	Anks1b	0.890078038	0.002347303
mNP	ENSMUSG00000021745	Ptprg	0.888740245	0.002552691
mNP	ENSMUSG00000031673	Cdh11	0.887959866	0.002663361
mNP	ENSMUSG00000036452	Arhgap26	0.887848384	0.002727635
mNP	ENSMUSG00000020227	Irak3	0.887736901	0.00246471
mNP	ENSMUSG00000051379	Flrt3	0.887625418	0.002761044
mNP	ENSMUSG00000037661	Gpr160	0.887625418	0.002716324
mNP	ENSMUSG00000032318	Isl2	0.886956522	0.002896702
mNP	ENSMUSG00000028381	Ugcg	0.886733556	0.002938763
mNP	ENSMUSG00000030499	Kctd15	0.886510591	0.002418451
mNP	ENSMUSG00000026435	Slc45a3	0.886510591	0.002975299
mNP	ENSMUSG00000021866	Anxa11	0.886510591	0.002958473
mNP	ENSMUSG00000021306	Gpr137b	0.88606466	0.003071328
mNP	ENSMUSG00000069670	Nkain2	0.885618729	0.002918846
mNP	ENSMUSG00000020422	Tns3	0.885618729	0.003060636
mNP	ENSMUSG00000056938	Acbd4	0.885618729	0.00316427
mNP	ENSMUSG00000000958	Slc7a7	0.884503902	0.003408215
mNP	ENSMUSG00000026991	Pkp4	0.883166109	0.003645976
mNP	ENSMUSG00000032035	Ets1	0.883166109	0.003578438
mNP	ENSMUSG00000059288	Cdyl	0.882497213	0.003818999
mNP	ENSMUSG00000040016	Ptger3	0.88238573	0.003799829
mNP	ENSMUSG00000045440	Insm2	0.882051282	0.00398558
mNP	ENSMUSG00000081951	NA	0.881939799	0.003544556
mNP	ENSMUSG00000054871	Tmem158	0.88115942	0.004246836
mNP	ENSMUSG00000059852	Kcng2	0.88115942	0.003966717
mNP	ENSMUSG00000074916	Chst14	0.880490524	0.004379114
mNP	ENSMUSG00000070639	Lrrc8b	0.880267559	0.004510332
mNP	ENSMUSG00000023000	Dhh	0.879821628	0.00416997
mNP	ENSMUSG00000027849	Syt6	0.8787068	0.004817071
mNP	ENSMUSG00000099686	NA	0.877591973	0.003713071
mNP	ENSMUSG00000041362	Shtn1	0.876700111	0.005677303
mNP	ENSMUSG00000025196	Cpn1	0.873801561	0.006439377
mNP	ENSMUSG00000104233	NA	0.873690078	0.006440682
mNP	ENSMUSG00000083892	NA	0.87335563	0.007069536
mNP	ENSMUSG00000094843	NA	0.872463768	0.007334693
mNP	ENSMUSG00000020698	Cct6b	0.872017837	0.006738514
mNP	ENSMUSG00000044164	Rnf182	0.871794872	0.007444208
mNP	ENSMUSG00000029217	Tec	0.871683389	0.007321723
mNP	ENSMUSG00000027646	Src	0.871571906	0.007944279

mNP	ENSMUSG00000037833	Sh2d4b	0.871125975	0.00768241
mNP	ENSMUSG00000027954	Efna1	0.870680045	0.008436263
mNP	ENSMUSG00000086825	NA	0.870345596	0.007997458
mNP	ENSMUSG00000018906	P4ha2	0.870011148	0.007679251
mNP	ENSMUSG00000040896	Kcnd3	0.869119287	0.009237491
mNP	ENSMUSG00000000058	Cav2	0.868673356	0.009581963
mNP	ENSMUSG00000023055	Calcoco1	0.868004459	0.010010279
mNP	ENSMUSG00000029007	Agtrap	0.867558528	0.010273871
mNP	ENSMUSG00000025558	Dock9	0.867112598	0.010588573
mNP	ENSMUSG00000032366	Tpm1	0.867112598	0.010594237
mNP	ENSMUSG00000030134	Rasgef1a	0.866666667	0.010895199
mNP	ENSMUSG00000044528	Tram1l1	0.866666667	0.010659575
mNP	ENSMUSG00000052698	Tln2	0.865774805	0.011528541
mNP	ENSMUSG00000101780	NA	0.865551839	0.011479529
mNP	ENSMUSG00000030098	Grip2	0.864882943	0.011460687
mNP	ENSMUSG00000025795	Rassf3	0.864882943	0.012094266
mNP	ENSMUSG00000023143	Nagpa	0.864659978	0.012324336
mNP	ENSMUSG00000045349	Sh2d5	0.863991081	0.012848376
mNP	ENSMUSG00000015335	Zdhhc12	0.863768116	0.013068871
mNP	ENSMUSG00000015652	Steap1	0.863768116	0.01150182
mNP	ENSMUSG00000016179	Camk1g	0.86309922	0.013608806
mNP	ENSMUSG00000034275	Igsf9b	0.862430323	0.014018871
mNP	ENSMUSG00000002365	Snx9	0.862430323	0.014215775
mNP	ENSMUSG00000022269	Mar-11	0.861984392	0.014543416
mNP	ENSMUSG00000102330	NA	0.861204013	0.012685355
mNP	ENSMUSG00000042712	Wbp5	0.861092531	0.015480413
mNP	ENSMUSG00000085795	Zfp703	0.861092531	0.014944992
mNP	ENSMUSG00000020605	Hs1bp3	0.861092531	0.015460788
mNP	ENSMUSG00000019951	Uhrf1bp1l	0.860869565	0.015695057
mNP	ENSMUSG00000003541	Ier3	0.8606466	0.015863196
mNP	ENSMUSG00000021301	Hecw1	0.8606466	0.015912522
mNP	ENSMUSG00000047045	Tmem164	0.860200669	0.016354476
mNP	ENSMUSG00000040479	Dgkz	0.859754738	0.01682165
mNP	ENSMUSG00000074796	Slc4a11	0.859754738	0.016793257
mNP	ENSMUSG00000040447	Spns2	0.859531773	0.015972121
mNP	ENSMUSG00000023909	Paqr4	0.859531773	0.017060754
mNP	ENSMUSG00000003279	Dlgap1	0.859085842	0.017503191
mNP	ENSMUSG00000005413	Hmox1	0.858862876	0.017768584
mNP	ENSMUSG00000042272	Sestd1	0.858416945	0.018275155
mNP	ENSMUSG00000056880	Gadl1	0.858416945	0.014737223
mNP	ENSMUSG00000025089	Gfra1	0.85819398	0.018206041
mNP	ENSMUSG00000019823	Mical1	0.857748049	0.019032221
mNP	ENSMUSG00000021432	Slc35b3	0.857525084	0.019262035

mNP	ENSMUSG00000032261	Sh3bgrl2	0.856856187	0.020125272
mNP	ENSMUSG00000021665	Hexb	0.856856187	0.020125272
mNP	ENSMUSG00000041930	Fam222a	0.85574136	0.021279315
mNP	ENSMUSG00000038280	Ostm1	0.855295429	0.022158698
mNP	ENSMUSG00000015243	Abca1	0.854849498	0.02277616
mNP	ENSMUSG00000010476	Ebf3	0.854403567	0.022944652
mNP	ENSMUSG00000030539	Sema4b	0.85328874	0.025088597
mNP	ENSMUSG00000008305	Tle1	0.852619844	0.026143719
mNP	ENSMUSG00000005087	Cd44	0.851059086	0.02874358
mNP	ENSMUSG00000051065	Mb21d2	0.851059086	0.02627932
mNP	ENSMUSG00000097240	NA	0.851059086	0.028708803
mNP	ENSMUSG00000023009	Nckap5l	0.85083612	0.029112012
mNP	ENSMUSG00000039662	Icmt	0.850613155	0.029550534
mNP	ENSMUSG00000074802	Gas2l3	0.850167224	0.029550534
mNP	ENSMUSG00000037759	Ptger2	0.850055741	0.028053996
mNP	ENSMUSG00000015839	Nfe2l2	0.849721293	0.031166585
mNP	ENSMUSG00000035245	Eogt	0.849498328	0.031282415
mNP	ENSMUSG00000056612	Ppp1r14b	0.849498328	0.031567158
mNP	ENSMUSG00000068523	Gng5	0.849052397	0.03240774
mNP	ENSMUSG00000035805	Mlc1	0.848940914	0.030383507
mNP	ENSMUSG00000054720	Lrrc8c	0.84793757	0.034679578
mNP	ENSMUSG00000007908	Hmgcll1	0.847714604	0.035071217
mNP	ENSMUSG00000047414	Flrt2	0.847603122	0.029741144
mNP	ENSMUSG00000020990	Cdkl1	0.847380156	0.033983848
mNP	ENSMUSG00000029735	Tpk1	0.847045708	0.036368677
mNP	ENSMUSG00000033983	Coil	0.846822742	0.036999289
mNP	ENSMUSG00000040631	Dok4	0.846822742	0.037076416
mNP	ENSMUSG00000101567	NA	0.846265329	0.032896585
mNP	ENSMUSG00000096719	Mrgpra2b	0.845930881	0.038636003
mNP	ENSMUSG00000024858	Adrbk1	0.845707915	0.039683297
mNP	ENSMUSG00000051236	Msrb3	0.845596433	0.035565066
mNP	ENSMUSG00000071567	NA	0.844593088	0.041552315
mNP	ENSMUSG00000030471	Zdhhc13	0.844370123	0.043007536
mNP	ENSMUSG00000047714	Ppp1r2	0.844370123	0.043017414
mNP	ENSMUSG00000033107	Rnf125	0.844147157	0.043589231
mNP	ENSMUSG00000041762	Gpr155	0.843924192	0.044134491
mNP	ENSMUSG00000023908	Pkmyt1	0.843924192	0.043925732
mNP	ENSMUSG00000032238	Rora	0.842363434	0.048103516
mNP	ENSMUSG00000050732	Vamp8	0.842028986	0.046386567
mNP	ENSMUSG00000024781	Lipa	0.841917503	0.049808688
mNP	ENSMUSG00000044243	Bhlha9	0.841360089	0.043537908
mNP	ENSMUSG00000044921	Rassf9	0.83690078	0.047936706
mNFa	ENSMUSG00000010803	Gabra1	0.996644295	9.75E-08

mNFa	ENSMUSG00000027765	P2ry1	0.996015101	1.29E-07
mNFa	ENSMUSG00000033998	Kcnk1	0.994127517	2.08E-07
mNFa	ENSMUSG00000038859	Baiap2l1	0.992869128	2.16E-07
mNFa	ENSMUSG00000002228	Ppm1j	0.986996644	3.20E-07
mNFa	ENSMUSG00000046480	Scn4b	0.984689597	4.46E-07
mNFa	ENSMUSG00000070687	Htr1d	0.983850671	3.94E-07
mNFa	ENSMUSG00000049404	Rarres1	0.98238255	5.76E-07
mNFa	ENSMUSG00000034891	Sncb	0.981963087	5.91E-07
mNFa	ENSMUSG00000059742	Kcnh7	0.981543624	6.51E-07
mNFa	ENSMUSG00000022176	Rem2	0.975671141	7.67E-07
mNFa	ENSMUSG00000096606	Tpbgl	0.974098154	9.89E-07
mNFa	ENSMUSG00000027833	Shox2	0.973888423	7.01E-07
mNFa	ENSMUSG00000048108	Tmem72	0.972734899	1.16E-06
mNFa	ENSMUSG00000039601	Rcan2	0.970427852	1.72E-06
mNFa	ENSMUSG00000030302	Atp2b2	0.967701342	2.18E-06
mNFa	ENSMUSG00000085007	Gm11549	0.966547819	1.41E-06
mNFa	ENSMUSG00000044252	Osbpl1a	0.962877517	3.34E-06
mNFa	ENSMUSG00000030088	Aldh1l1	0.959312081	3.63E-06
mNFa	ENSMUSG00000031837	Necab2	0.958263423	3.93E-06
mNFa	ENSMUSG00000054459	Vsnl1	0.958053691	4.14E-06
mNFa	ENSMUSG00000086847	Tbx3os2	0.957005034	3.68E-06
mNFa	ENSMUSG00000039252	Lgi2	0.956690436	3.76E-06
mNFa	ENSMUSG00000024065	Ehd3	0.955746644	6.10E-06
mNFa	ENSMUSG00000039911	Spsb1	0.953229866	7.53E-06
mNFa	ENSMUSG00000070570	Slc17a7	0.953125	5.48E-06
mNFa	ENSMUSG00000047842	Diras2	0.94966443	1.00E-05
mNFa	ENSMUSG00000019528	Gyg	0.949244966	1.05E-05
mNFa	ENSMUSG00000046699	Slitrk4	0.947567114	7.69E-06
mNFa	ENSMUSG00000041112	Elmo1	0.945260067	1.36E-05
mNFa	ENSMUSG00000034648	Lrrn1	0.944001678	1.62E-05
mNFa	ENSMUSG00000034127	Tspan8	0.943896812	1.49E-05
mNFa	ENSMUSG00000063239	Grm4	0.942533557	1.33E-05
mNFa	ENSMUSG00000030246	Ldhb	0.942323826	1.85E-05
mNFa	ENSMUSG00000041115	Iqsec2	0.941484899	1.98E-05
mNFa	ENSMUSG00000017400	Stac2	0.941065436	1.91E-05
mNFa	ENSMUSG00000046442	Ppm1e	0.940645973	1.97E-05
mNFa	ENSMUSG00000056258	Kcnq3	0.938548658	2.17E-05
mNFa	ENSMUSG00000066438	Plekhd1	0.938129195	2.60E-05
mNFa	ENSMUSG00000051855	Mest	0.937080537	2.81E-05
mNFa	ENSMUSG00000029359	Tesc	0.937080537	2.78E-05
mNFa	ENSMUSG00000025090	Ccdc172	0.936451342	2.06E-05
mNFa	ENSMUSG00000021696	Elovl7	0.935192953	3.30E-05
mNFa	ENSMUSG00000057134	Ado	0.934983221	3.35E-05

mNFa	ENSMUSG00000032028	Nxpe2	0.934249161	2.69E-05
mNFa	ENSMUSG00000034765	Dusp5	0.932676174	4.01E-05
mNFa	ENSMUSG00000040420	Cdh18	0.93204698	4.22E-05
mNFa	ENSMUSG00000028773	Fabp3	0.931208054	4.53E-05
mNFa	ENSMUSG00000071648	Rom1	0.928691275	5.51E-05
mNFa	ENSMUSG00000045104	NA	0.927432886	5.95E-05
mNFa	ENSMUSG00000019966	Kitl	0.925545302	5.99E-05
mNFa	ENSMUSG00000055044	Pdlim1	0.925020973	6.78E-05
mNFa	ENSMUSG00000044749	Abca6	0.924391779	7.70E-05
mNFa	ENSMUSG00000042757	Tmem108	0.924286913	5.35E-05
mNFa	ENSMUSG00000059895	Ptp4a3	0.924077181	7.94E-05
mNFa	ENSMUSG00000059146	Ntrk3	0.922713926	6.30E-05
mNFa	ENSMUSG00000027489	Necab3	0.922189597	9.17E-05
mNFa	ENSMUSG00000026547	Tagln2	0.920302013	0.000102772
mNFa	ENSMUSG00000020553	Pctp	0.919672819	0.000111866
mNFa	ENSMUSG00000038949	Cnst	0.917365772	0.000134048
mNFa	ENSMUSG00000058966	Fam57b	0.916736577	0.000140679
mNFa	ENSMUSG00000047976	Kcna1	0.91642198	0.000137801
mNFa	ENSMUSG00000090546	Cdr1	0.915058725	0.000159862
mNFa	ENSMUSG00000073910	Mob3b	0.914953859	0.000143716
mNFa	ENSMUSG00000074505	Fat3	0.914639262	0.000133416
mNFa	ENSMUSG00000028228	Cpne3	0.914639262	0.000165108
mNFa	ENSMUSG00000033615	Cplx1	0.913800336	0.000176002
mNFa	ENSMUSG00000054763	Defb42	0.913590604	0.000171419
mNFa	ENSMUSG00000020151	Ptprr	0.912122483	0.000199653
mNFa	ENSMUSG00000026344	Lypd1	0.911912752	0.000154928
mNFa	ENSMUSG00000037706	Cd81	0.91170302	0.000204906
mNFa	ENSMUSG00000021991	Cacna2d3	0.911493289	0.000203517
mNFa	ENSMUSG00000027562	Car2	0.909920302	0.000232182
mNFa	ENSMUSG00000059412	Fxyd2	0.908557047	0.000262089
mNFa	ENSMUSG00000017978	Cadps2	0.907403523	0.000277859
mNFa	ENSMUSG00000019194	Scn1b	0.907298658	0.000288036
mNFa	ENSMUSG00000091636	A330050F15Rik	0.904572148	0.000216246
mNFa	ENSMUSG00000033377	Palmd	0.901845638	0.000430135
mNFa	ENSMUSG00000055069	Rab39	0.901426174	0.000446509
mNFa	ENSMUSG00000027827	Kcnab1	0.901426174	0.00044601
mNFa	ENSMUSG00000025511	Tspan4	0.901216443	0.000453472
mNFa	ENSMUSG00000020623	Map2k6	0.901216443	0.000413879
mNFa	ENSMUSG00000045967	Gpr158	0.900587248	0.000473874
mNFa	ENSMUSG00000018604	Tbx3	0.900272651	0.000472566
mNFa	ENSMUSG00000042388	Dlgap3	0.899748322	0.000502556
mNFa	ENSMUSG00000033595	Lgi3	0.899433725	0.000456444
mNFa	ENSMUSG00000029544	Cabp1	0.899119128	0.000527702

mNFa	ENSMUSG00000082160	NA	0.898909396	0.000487604
mNFa	ENSMUSG00000026347	Tmem163	0.897860738	0.000532103
mNFa	ENSMUSG00000023033	Scn8a	0.897860738	0.000578426
mNFa	ENSMUSG00000019853	Hebp2	0.897651007	0.000588531
mNFa	ENSMUSG00000026678	Rgs5	0.897231544	0.000496671
mNFa	ENSMUSG00000004748	Mtfp1	0.896602349	0.000630427
mNFa	ENSMUSG00000045991	Onecut2	0.895973154	0.000605577
mNFa	ENSMUSG00000038205	Prkab2	0.895763423	0.000675859
mNFa	ENSMUSG00000047786	Lix1	0.895553691	0.000686273
mNFa	ENSMUSG00000021565	Slc6a19	0.895134228	0.000678836
mNFa	ENSMUSG00000100851	NA	0.893980705	0.000550501
mNFa	ENSMUSG00000030268	Bcat1	0.893666107	0.000756357
mNFa	ENSMUSG00000055254	Ntrk2	0.893036913	0.000820179
mNFa	ENSMUSG00000042734	Ttc9	0.891778523	0.000902735
mNFa	ENSMUSG00000055022	Cntn1	0.890729866	0.000958543
mNFa	ENSMUSG00000053889	NA	0.890520134	0.000959212
mNFa	ENSMUSG00000020486	Sep-04	0.890310403	0.001000168
mNFa	ENSMUSG00000027895	Kcnc4	0.889156879	0.001076365
mNFa	ENSMUSG00000021798	Ldb3	0.888527685	0.001133129
mNFa	ENSMUSG00000045287	Rtn4rl1	0.887793624	0.001190362
mNFa	ENSMUSG00000085438	1700020I14Rik	0.887793624	0.001201115
mNFa	ENSMUSG00000032135	Mcam	0.886535235	0.001288603
mNFa	ENSMUSG00000047606	Ankrd34c	0.886220638	0.001033136
mNFa	ENSMUSG00000004633	Chn2	0.884752517	0.001429867
mNFa	ENSMUSG00000106379	Lhfpl3	0.883389262	0.001643909
mNFa	ENSMUSG00000035232	Pdk3	0.882969799	0.001693196
mNFa	ENSMUSG00000029108	Pcdh7	0.882969799	0.001490598
mNFa	ENSMUSG00000034810	Scn7a	0.882340604	0.00177075
mNFa	ENSMUSG00000022212	Cpne6	0.880662752	0.001987949
mNFa	ENSMUSG00000002980	Bcam	0.880033557	0.002081255
mNFa	ENSMUSG00000058624	Gda	0.87971896	0.001924901
mNFa	ENSMUSG00000031688	Pou4f2	0.879509228	0.002006419
mNFa	ENSMUSG00000021062	Rab15	0.877307047	0.002519892
mNFa	ENSMUSG00000045333	Zfp423	0.876782718	0.002559224
mNFa	ENSMUSG00000006589	Aprt	0.876677852	0.002614782
mNFa	ENSMUSG00000036766	Dner	0.876468121	0.002669971
mNFa	ENSMUSG00000061013	Mkx	0.876468121	0.00237891
mNFa	ENSMUSG00000001666	Ddt	0.876258389	0.00270969
mNFa	ENSMUSG00000035513	Ntng2	0.875104866	0.002374455
mNFa	ENSMUSG00000032724	Abtb2	0.874580537	0.003040533
mNFa	ENSMUSG00000038718	Pbx3	0.874370805	0.00271784
mNFa	ENSMUSG00000015944	Gatsl2	0.873427013	0.003289612
mNFa	ENSMUSG00000037747	Phyhipl	0.873112416	0.003362929

mNFa	ENSMUSG00000047766	Lrrc49	0.87227349	0.003563192
mNFa	ENSMUSG00000042195	Slc35f2	0.871434564	0.00371315
mNFa	ENSMUSG00000021701	Plk2	0.871224832	0.003825857
mNFa	ENSMUSG00000032011	Thy1	0.871015101	0.003833715
mNFa	ENSMUSG00000008822	Acyp1	0.870385906	0.00405085
mNFa	ENSMUSG00000052914	Cyp2j6	0.870176174	0.004107889
mNFa	ENSMUSG00000030616	Sytl2	0.869232383	0.004168677
mNFa	ENSMUSG00000024112	Cacna1h	0.869127517	0.004399831
mNFa	ENSMUSG00000091498	NA	0.869127517	0.004413613
mNFa	ENSMUSG00000041482	Piezo2	0.868708054	0.004425451
mNFa	ENSMUSG00000060257	Scrt2	0.867030201	0.005077562
mNFa	ENSMUSG00000053199	Arhgap20	0.866086409	0.005422652
mNFa	ENSMUSG00000044024	Rell2	0.865876678	0.005504931
mNFa	ENSMUSG00000029608	Rph3a	0.864932886	0.005844084
mNFa	ENSMUSG00000047963	Stbd1	0.864723154	0.005948455
mNFa	ENSMUSG00000028701	Lurap1	0.864408557	0.005733591
mNFa	ENSMUSG00000020684	Rasl10b	0.864198826	0.005690956
mNFa	ENSMUSG00000025810	Nrp1	0.863674497	0.006252799
mNFa	ENSMUSG00000010080	Epn3	0.863674497	0.005904846
mNFa	ENSMUSG00000057315	Arhgap24	0.863359899	0.006514212
mNFa	ENSMUSG00000042873	Lhfpl4	0.862416107	0.006951852
mNFa	ENSMUSG00000048899	Rimkla	0.86136745	0.007456235
mNFa	ENSMUSG00000038174	Fam126b	0.860213926	0.008048361
mNFa	ENSMUSG00000004558	Ndrg2	0.859479866	0.008452599
mNFa	ENSMUSG00000000552	Zfp385a	0.859479866	0.007875159
mNFa	ENSMUSG00000019478	Rab4a	0.859060403	0.008688586
mNFa	ENSMUSG00000002233	Rhoc	0.859060403	0.008688586
mNFa	ENSMUSG00000035407	Kank4	0.85864094	0.008186862
mNFa	ENSMUSG00000038264	Sema7a	0.85864094	0.00739916
mNFa	ENSMUSG00000048489	8430408G22Rik	0.858011745	0.007116327
mNFa	ENSMUSG00000079056	Kcnip3	0.857592282	0.009573502
mNFa	ENSMUSG00000028982	Slc25a33	0.857592282	0.009573502
mNFa	ENSMUSG00000035653	Lrfn5	0.857592282	0.00956255
mNFa	ENSMUSG00000035246	Pcyt1b	0.85738255	0.00970509
mNFa	ENSMUSG00000033208	S100b	0.85738255	0.009703477
mNFa	ENSMUSG00000049922	Slc35c1	0.856753356	0.010105639
mNFa	ENSMUSG00000036395	Glb1l2	0.856438758	0.008004194
mNFa	ENSMUSG00000033427	Upb1	0.856229027	0.010430084
mNFa	ENSMUSG00000079499	6530402F18Rik	0.855704698	0.008168449
mNFa	ENSMUSG00000075334	Rprm	0.854865772	0.011404904
mNFa	ENSMUSG00000047986	Palm3	0.854760906	0.011046836
mNFa	ENSMUSG00000106055	NA	0.854236577	0.011924246
mNFa	ENSMUSG00000032252	Glce	0.852768456	0.013115161

mNFa	ENSMUSG00000034224	Slc38a8	0.852558725	0.010262496
mNFa	ENSMUSG00000063694	Cycs	0.85192953	0.013855122
mNFa	ENSMUSG00000061878	Sphk1	0.851510067	0.014143476
mNFa	ENSMUSG00000021373	Cap2	0.85119547	0.011388494
mNFa	ENSMUSG00000101693	Gm19461	0.850880872	0.014798271
mNFa	ENSMUSG00000032452	Clstn2	0.849832215	0.01501389
mNFa	ENSMUSG00000043670	Diras1	0.848573826	0.017195636
mNFa	ENSMUSG00000004031	Brinp2	0.84846896	0.017220818
mNFa	ENSMUSG00000028631	Kcnq4	0.848364094	0.013928036
mNFa	ENSMUSG00000020297	Nsg2	0.847944631	0.017888164
mNFa	ENSMUSG00000033781	Asb13	0.847105705	0.018818576
mNFa	ENSMUSG00000060487	Samd5	0.84647651	0.017603035
mNFa	ENSMUSG00000049775	Tmsb4x	0.846266779	0.019914647
mNFa	ENSMUSG00000068220	Lgals1	0.846266779	0.019914647
mNFa	ENSMUSG00000024798	Htr7	0.846266779	0.015345793
mNFa	ENSMUSG00000036206	Sh3bp4	0.846266779	0.019888404
mNFa	ENSMUSG00000033676	Gabrb3	0.845427852	0.021009666
mNFa	ENSMUSG00000022844	Pdia5	0.845113255	0.021351211
mNFa	ENSMUSG00000049422	Chchd10	0.845008389	0.021575855
mNFa	ENSMUSG00000030270	Cpne9	0.844169463	0.020819469
mNFa	ENSMUSG00000028710	Atpaf1	0.843330537	0.024022186
mNFa	ENSMUSG00000064329	Scn1a	0.842806208	0.023244535
mNFa	ENSMUSG00000060402	Chst8	0.842491611	0.025298531
mNFa	ENSMUSG00000042182	Bend6	0.841862416	0.026348295
mNFa	ENSMUSG00000071719	Tmem28	0.84175755	0.024142888
mNFa	ENSMUSG00000026442	Nfasc	0.841652685	0.026680863
mNFa	ENSMUSG00000087651	1500009L16Rik	0.841442953	0.026886434
mNFa	ENSMUSG00000020836	Coro6	0.841128356	0.026369835
mNFa	ENSMUSG00000029001	Fbxo44	0.840604027	0.028524631
mNFa	ENSMUSG00000062760	1810041L15Rik	0.839555369	0.030448657
mNFa	ENSMUSG00000040164	Kcns1	0.839450503	0.025239626
mNFa	ENSMUSG00000039835	Nhsl1	0.839240772	0.030662685
mNFa	ENSMUSG00000031824	6430548M08Rik	0.83903104	0.031451932
mNFa	ENSMUSG00000043673	Kcns3	0.83829698	0.032908045
mNFa	ENSMUSG00000062054	Iah1	0.838087248	0.033342857
mNFa	ENSMUSG00000027350	Chgb	0.837667785	0.034222686
mNFa	ENSMUSG00000049556	Lingo1	0.837038591	0.035290928
mNFa	ENSMUSG00000032523	Hhatl	0.836199664	0.034142862
mNFa	ENSMUSG00000075605	2300005B03Rik	0.835360738	0.031875669
mNFa	ENSMUSG00000021396	Nxnl2	0.835360738	0.034548901
mNFa	ENSMUSG00000020396	Nefh	0.835151007	0.040018553
mNFa	ENSMUSG00000031231	Cox7b	0.835151007	0.040018553
mNFa	ENSMUSG00000000631	Myo18a	0.833473154	0.044400838

mNFa	ENSMUSG00000042659	Arrdc4	0.832319631	0.047638095
mNFa	ENSMUSG00000023169	Slc38a1	0.831795302	0.047720215
